# Meningioma animal models: a systematic review and meta-analysis

**DOI:** 10.1186/s12967-023-04620-7

**Published:** 2023-10-28

**Authors:** Mikkel Schou Andersen, Mikkel Seremet Kofoed, Asger Sand Paludan-Müller, Christian Bonde Pedersen, Tiit Mathiesen, Christian Mawrin, Martin Wirenfeldt, Bjarne Winther Kristensen, Birgitte Brinkmann Olsen, Bo Halle, Frantz Rom Poulsen

**Affiliations:** 1 Department of Neurosurgery, Odense University Hospital, Odense, Denmark; 2 BRIDGE (Brain Research - Inter Disciplinary Guided Excellence), University of Southern Denmark, Odense, Denmark; 3 Nordic Cochrane Centre, Rigshospitalet, Copenhagen University, Copenhagen, Denmark; 4 Department of Neurosurgery, Rigshospitalet, Copenhagen University, Copenhagen, Denmark; 5 Department of Neuropathology, Otto-Von-Guericke University, Magdeburg, Germany; 6 Department of Pathology and Molecular Biology, Hospital South West Jutland, Esbjerg, Denmark; 7 Department of Neuropathology, Rigshospitalet, Copenhagen University, Copenhagen, Denmark; 8 Clinical Physiology and Nuclear Medicine, Odense University Hospital, Odense, Denmark; 9 Department of Clinical Research, University of Southern Denmark, Odense, Denmark; 10 Centre for Evidence-Based Medicine Odense (CEBMO) and NHTA: Market Access & Health Economics Consultancy, Copenhagen, Denmark; 11 Department of Regional Health Research, University of Southern, Odense, Denmark; 12 Department of Surgical Pathology, Zealand University Hospital, Roskilde, Denmark

**Keywords:** Meningioma animal model, Xenograft, Genetically engineered model, Systematic review, Meta-analysis

## Abstract

**Background:**

Animal models are widely used to study pathological processes and drug (side) effects in a controlled environment. There is a wide variety of methods available for establishing animal models depending on the research question. Commonly used methods in tumor research include xenografting cells (established/commercially available or primary patient-derived) or whole tumor pieces either orthotopically or heterotopically and the more recent genetically engineered models—each type with their own advantages and disadvantages. The current systematic review aimed to investigate the meningioma model types used, perform a meta-analysis on tumor take rate (TTR), and perform critical appraisal of the included studies. The study also aimed to assess reproducibility, reliability, means of validation and verification of models, alongside pros and cons and uses of the model types.

**Methods:**

We searched Medline, Embase, and Web of Science for all in vivo meningioma models. The primary outcome was tumor take rate. Meta-analysis was performed on tumor take rate followed by subgroup analyses on the number of cells and duration of incubation. The validity of the tumor models was assessed qualitatively. We performed critical appraisal of the methodological quality and quality of reporting for all included studies.

**Results:**

We included 114 unique records (78 using established cell line models (ECLM), 21 using primary patient-derived tumor models (PTM), 10 using genetically engineered models (GEM), and 11 using uncategorized models). TTRs for ECLM were 94% (95% CI 92–96) for orthotopic and 95% (93–96) for heterotopic. PTM showed lower TTRs [orthotopic 53% (33–72) and heterotopic 82% (73–89)] and finally GEM revealed a TTR of 34% (26–43).

**Conclusion:**

This systematic review shows high consistent TTRs in established cell line models and varying TTRs in primary patient-derived models and genetically engineered models. However, we identified several issues regarding the quality of reporting and the methodological approach that reduce the validity, transparency, and reproducibility of studies and suggest a high risk of publication bias. Finally, each tumor model type has specific roles in research based on their advantages (and disadvantages).

Systematic review registration: PROSPERO-ID CRD42022308833.

**Supplementary Information:**

The online version contains supplementary material available at 10.1186/s12967-023-04620-7.

## Introduction

Meningiomas account for 40% of all primary intracranial tumors [1] and are primarily benign (90–95%). They display as a group a heterogenous epigenetic/genetic profiles [2–7]. The preferred treatment for a symptomatic and/or growing meningioma is surgery [8, 9]. Pharmaceutical therapies are primarily used pre-clinically or in protocolled trials and have so far had limited success in humans [2]. In vivo research is thus paramount.

While a perfectly designed tumor animal model is a utopian thought, we can strive towards a model that resembles the actual tumor as closely as possible. Scientific advances over the last 70 years have led to huge progress in the field of animal models, including models for meningioma. The first successes in establishing a meningioma model via xenotransplantation occurred in the 1940s and 50 s [10, 11], and studies over the next 30 years have used a variety of approaches [12–19].

The availability of immunodeficient animals and established immortalized cell-lines (i.e. IOMM-Lee, CH-157, and BEN-MEN-1) has revolutionized the field in terms of tumor take rate (TTR) (which is close to 100% in many cases) and has led to stable models for testing new treatment options [20–22]. The use of immortalized cells provides a needed weapon against senescence, but at the same time it limits conclusions as to treatment effects, and many pharmaceutical successes from animal studies have proven ineffective in human clinical trials [23]. The use of primary cell models without the use of immortalization provides a patient-specific model for more targeted therapies. However, these approaches lack immune system interaction and show varying TTRs, especially for benign primary tumors [24–26]. The first genetically engineered (meningioma) model (GEM) emerged twenty years ago using a conditional knockout *Nf2*^*Flox2/Flox2*^ via recombinant high-titer adenovirus expressing Cre recombinase (AdCre) to ensure growth [27], with a few papers (primarily from the same group) replicating the results [28–31]. Although GEM provides a solution to the significant problem of adaptive immune system involvement, its low TTRs and long tumor induction time limit the model’s use in treatment studies.

So far, a few narrative reviews [32–34] and a systematic review have been conducted on the topic [35]. However, the systematic review did not entail a search through multiple literature platforms and did not assess methodological quality/risk of bias. In the current paper, we present the first systematic review and meta-analysis of meningioma animal models that includes an assessment of the quality of reporting and the methodological quality of the included studies.

The overall aim of the review was to investigate which method of tumor development/growth—established patient-derived cell lines (orthotopic and heterotopic), primary patient-derived tumor cells/material (orthotopic and heterotopic), or genetically modified animals—provides the best tumor take rate and at what duration of incubation. The specific objectives were firstly, to search the literature for all available research regarding the different models and to assess their reproducibility and reliability, advantages, and disadvantages. Secondly, to determine how the models should be verified and which modalities are necessary to compare xenograft or genetically modified tumors to the parent tumor. Thirdly, to present possible future aspects of meningioma animal models in relation to optimal tests of future therapeutics in human meningiomas. Fourthly, to identify and analyze knowledge gaps that could help identify future research initiatives.

## Methods

### Protocol and registration

This systematic review was performed in accordance with the updated 2020 Preferred Reporting Items for Systematic Reviews and Meta-Analyses (PRISMA) guidelines [36]. The PRISMA checklist and PRISMA abstract checklists are provided in the Additional files 1 and 2). The review was prospectively registered with PROSPERO at https://www.crd.york.ac.uk/PROSPERO/ as an animal study (CRD42022308833) prior to first full-text screening on February 14, 2022. The full original protocol that was uploaded to PROSPERO is available in the Additional file 3. The minor changes to the original uploaded protocol are reported in the Additional file 4.

### Eligibility criteria

We sought to include all original records published in peer-reviewed journals with full texts that had the Population and Outcome (PO) of in vivo experiments with the intention of meningioma growth (population) and the corresponding meningioma growth rate and induction duration (outcome).

We excluded review records, systematic reviews, human studies, and conference abstracts as well as records describing spontaneous meningiomas in animals without the use of genetic modification specifically aimed at meningiomas. Models that were not meningioma models were excluded. No restrictions were applied to study design as long as the record described an in vivo experiment aiming for meningioma growth. We included records in any language if an English title or abstract was available.

### Information sources and search strategy

We searched the following electronic databases on June 18, 2021, and again on August 8, 2022, (the latter with the limitations set to 2021–2022): Medline, Embase, and Web of Science. The search strategy was reviewed by a research librarian at the University of Southern Denmark. Search terms were sourced from already published papers that we were familiar with, e.g. meningioma animal model, xenograft, and genetically engineered model. The full original search strategy is available in the Additional file 5.

### Study selection

All papers extracted via the search string were screened by title and abstract by two reviewers (MSA and MSK) in a blinded fashion. During the first round of screening (title and abstract), all papers that were deemed eligible by either of the authors were included for the second round (full-text screening). Cohen’s Kappa index [37] was performed to assess initial screening agreement. The first 200 papers were rated un-blinded to adjust the screening method. Full-text screening was performed in a blinded fashion by two reviewers (MSA and MSK), with disagreements being settled by discussion with a senior author (FRP).

Papers were divided into four categories: established cell line models (ECLM), primary patient-derived tumor models (PTM), genetically engineered models (GEM), and other models not fitting into the previous three categories (uncategorized). Papers eligible for inclusion but older than 40 years were ultimately excluded due lack of relevance. Records containing more than one animal model type were included in all relevant categories.

### Data extraction fields

A pre-defined data extraction sheet was developed for each model type. Data were extracted independently by two reviewers (MSA and MSK) in a blinded fashion. Data were extracted on record meta-data, animal data, and tumor model characteristics. The data extraction sheet (including a description of the fields) is available in the Additional file 6.

### Synthesis of results and summary measures

All analyses were conducted using the freely available software R (https://www.r-project.org/). We performed random-effect meta-analyses on the proportion of animals that developed tumors (TTR) for five model types: ECLM (orthotopic and heterotopic separately), PTM (orthotopic and heterotopic separately), and GEM. Meta-analyses used the metaprop function from the meta package.

Heterogeneity was assessed by visual inspection of forest plots and by using the I^2^ measure as recommended in the Cochrane Handbook [38]. If heterogeneity was identified, it was examined through subgroup analyses. The following characteristics were examined as potential sources of heterogeneity in xenografted models: number of cells, cell concentration, established cell line used, parent tumor grade (WHO grade 1–3), and duration of incubation. For GEMs, the following characteristics were examined in subgroup analyses: genomic lesion type, method of gaining lesion, incubation time.

Due to the risk of confounding, all subgroup analyses were considered exploratory and were interpreted cautiously. Survival studies on duration of incubation were excluded from the meta-analyses (apart from GEMs, which were mainly survival studies) and commented on narratively. The validity of different tumor models and their relation to human tumors were discussed narratively, including a discussion of the methodological quality of relevant trials. The results of the mixed category ‘uncategorized’, which contains various tumor models, were presented narratively due to significant heterogeneity.

### Critical appraisal of quality of reporting, methodological quality, and risk of bias

As no validated tool exists for the critical assessment of the types of records included in this review, we developed a critical appraisal tool (**Cri**tical appraisal of **me**thodological quality and **q**uality of reporting—CRIME-Q) [39] for use in this review and other animal reviews in the future. CRIME-Q is inspired primarily by Macleod et al. [40] and the ARRIVE 2.0 guidelines for Animal Research: Reporting of In Vivo experiments [41], but it also includes items from the recommended and validated SYRCLE’s Risk of Bias tool [42].

Two reviewers (MSA and MSK) individually used CRIME-Q to assess all included studies. Any discrepancies were solved via a third reviewer (FRP). The domains included in CRIME-Q are described in Table 1. We summarized the overall quality of included studies narratively and included this in the interpretation of our results. A full table of all assessed studies are available in the Additional file 7. A predetermined list of information needed to obtain the various grades of Yes/No/Partly/Unclear/Not applicable (NA) is available in the Additional file 8.

**Table 1:** Critical Appraisal of Methodological Quality and Quality of reporting (CRIME-Q): categories, descriptions, and potential bias/impact on studies

	Category	Type	Questions and clarification	Potential for bias
1X	Peer review	QoR	Did the paper undergo peer review prior to publication? Peer review might be useful for detecting errors or fraud. **Yes/No**	With peer review: Bias against negative studies Without: potential for errors and/or fraud
2X	Bench-top/laboratory work related to establishing model—reporting	QoR	Was the study’s bench-top protocol sufficiently described (transparent, reproducible)? If e.g., cells are involved, did the study e.g., present incubator settings, description in detail of how the cells were treated (transfection, irradiation, etc.)? or describe how the cells were handled? Or for instance, did the study describe how to obtain a certain genetic model given there is no commercially available animal model? **Yes/Partly/No**	In vivo results are highly influenced by in vitro*/*bench-top part of the study. If not transparent and reproducible this lessens the usability of the study
2Y	Bench-top/laboratory work related to establishing model—methodology	MQ	Was the bench-top protocol feasible and well performed in relation to the experiment? Was it likely that the intended aim could be obtained based on the bench-top method? **Yes/Partly/No.** Studies with poor reporting (2X) will have difficulty gaining a high 2Y because of low transparency and the ability to assess method quality	If the bench-top protocol was not feasible, TTRs may be misleading, and readers should use papers with caution
3X	Animals—reporting	QoR	Were the animals used in the experiment sufficiently described? Were all parameters: Type, breed, age, weight, and manufacturer sufficiently described? If type, age, and weight were sufficiently described OR if weight was missing, but the manufacturer was included a Yes was given. If only partly described, then Partly. and if we were not able to correctly identify the animals No was given. **Yes/Partly/No**	The importance of the description of animal type cannot be understated since the immunological profile differs from strain to strain, which influences results
3Y	Animals—Methodology	MQ	Did the study use similar baseline characteristics for the animals (age, weight, type)? **Yes/Partly/No.** Studies with poor reporting (3X) will have difficulty gaining a high 3Y because of low transparency and the ability to assess method quality	If animals were not homogenous, study results might vary, for instance, low weight might result in poorer survival, which skews results
3Z	Selection bias (baseline characteristics) **(SYRCLE Item 2)**	RoB	Was the distribution of relevant baseline characteristics balanced between groups? I.e., was the distribution of e.g., male:female ratio, species, strain, age, and weight equally distributed throughout groups? **Yes/No/Unclear/NA**. Not applicable to studies using only one group	Unequal groups in intervention studies can skew results – introduces variables that potentially affect study results
4Y	Sample size	MQ	Did the study include a calculation of sample size? Describe how it was calculated – at what power? Was it appropriate and well performed? **Yes/Partly/No**	Studies may prove under-/overpowered in terms of drug efficacy if too few/many animals were used
5X	In vivo design and performance—reporting	QoR	Description of the in vivo study part: Were the surgery, implantation/injection method, and duration (whole experiment) sufficiently described? Is the study transparent and reproducible? **Yes/Partly/No**	The results will be difficult to replicate if the study is poorly described. Meaning the study is difficult to properly be assessed as a useful base for further research
5Y	In vivo design and performance—methodology	MQ	Did the method seem feasible and well performed concerning the study’s aim and outcome and in contrast to other known literature? Is it likely that the in vivo study design influences the results—incomprehensive/insensible method? **Yes/Partly/No** Studies with poor reporting (5X) will have difficulty gaining a high 5Y because of low transparency and the ability to assess method quality	A poor methodology can skew results making conclusions in relation to aims obsolete
5Z(1)	Selection bias (Sequence generation) **(SYRCLE Item 1)**	RoB	Was there a description of allocation (the process by which experimental units are assigned to experimental groups)—And was it appropriate? Not applicable to non-intervention studies. **Yes/No/Unclear/NA**	Unequal groups in intervention studies can skew results—introduces variables that potentially affect study results
5Z(2)	Performance bias (Random housing) **(SYRCLE Item 4)**	RoB	Were the animals randomly housed during the experiment? Yes/no/unclear. Not applicable to non-intervention studies. **Yes/No/Unclear/NA**	Some types of experiments are influenced by the location of housing, hence random assignment of placement could negate these issues
5Z(3)	Detection bias (Random outcome assessment) **(SYRCLE Item 6)**	RoB	Were animals randomly selected for outcome? For instance, If human endpoints (i.e., poor conditions, weight, etc.) were met and the investigators were not blinded, then the outcome cannot be assessed randomly. Not applicable to non-intervention studies. **Yes/No/Unclear/NA**	Bias toward assessing intervention effect size
6X	Compliance with animal welfare regulations	QoR	Did the study comply with any animal welfare regulations? **Yes/Partly/No**	Assurance of proper animal care throughout the study. Also important in terms of survival studies (human endpoints vs. death)
7X	Blinding	QoR	Was the study blinded in any way? Was the outcome assessed in a blinded fashion? Were the animals randomly selected across all groups of e.g., intervention? Were the investigator or animal handlers blinded? More specific blinding is listed below in 7Z(1–3) **Yes/Partly/No**	Unblinded administrator of intervention can skew results in both a positive and negative direction
7Z(1)	Performance bias (Blinding) **(SYRCLE Item 5)**	RoB	Describe all used means, if any, to blind trial caregiver and researchers from knowing which intervention each animal received. **Yes/No/Unclear/NA**. Not applicable for non-intervention studies, however, it could be applicable for instance in xenograft studies, where multiple patient samples were used	Animal handling may be affected by unblinded study design
7Z(2)	Allocation bias (allocation concealment) **(SYRCLE Item 3)**	RoB	Could the investigator allocating the animals to intervention or control group not foresee assignment? **Yes/No/Unclear/NA.** Not applicable to non-intervention studies. Yes/no/unclear/NA. This could be applicable for instance in xenograft studies, where multiple patient samples were used	In relation to 7Z (1). Selection, handling, and treatment of animals may be affected if allocation concealment was not adequately performed
7Z(3)	Detection bias (blinding) **(SYRCLE Item 7)**	RoB	Was the outcome assessor blinded? and could the blinding have been broken? Describe all measures used, if any, to blind outcome assessors from knowing which intervention each animal received. Were the outcome assessment methods the same in each group? **Yes/No/Unclear/NA**. This could be applicable to instance in xenograft studies, where multiple patient samples where used	Measurement of the outcome can be over-/underestimated if proper blinded outcome assessment was not performed
8X	Congruency between methods and results	QoR	Did the study present all their findings based on the methods described? Is there congruency between the method and results sections? **Yes/Partly/No**	Presenting results in which methods are not described is not transparent and replicable and should be interpreted with caution
8Z(1)	Attrition bias (incomplete outcome data) **(SYRCLE Item 8)**	RoB	Describe the completeness of outcome data including attrition and exclusions from the analysis and were incomplete outcome data adequately described? Were all animals included in the analysis and if not, was it described why they were not included? **Yes/No/Unclear**	Attritions and/or exclusions should be clearly described, i.e., the number of animals used. If not, study results become difficult to assess. Poor replicability and transparency
8Z(2)	Reporting bias (Selective outcome reporting) **(SYRCLE Item 9)**	RoB	Was the study protocol available (require a description of protocol location in the record) and were all of the study’s pre-specified primary and secondary outcomes reported in the manuscript? Was the study protocol not available but was it clear that the published report included all expected outcomes (i.e., comparing methods and results sections)?The study report fails to include results for a key outcome that would be expected to have been reported for such a study, i.e. tumor-take rate in transplantation experiments. **Yes/No/Unclear**	Congruency between results and methods should be carefully described to avoid reporting bias. If key outcomes for a certain method were not described, study validity, transparency, and replicability become difficult
9X	Presentation of limitations	QoR	Did the study contain a section of limitations, or did they comment on the limitations of the study in relationship to in vitro and/or in vivo subparts? **Yes/Partly/No**	No study is without limitations, and it is paramount to present them to the reader for transparency’s sake
10X	Statement of potential conflict of interest	QoR	Did the study contain a statement of potential conflicts of interest? **Yes/No**	Potential conflicts of interest can skew results i.e. if an investigator has a method patent or is paid by a certain pharmaceutical company, hence it is important for transparency’s sake to include it in the study
10Z	Publication bias (influence) (**SYRCLE Item 10)**	RoB	Inappropriate influence of funders or biased by companies. Was the study free of inappropriate influence from funders or companies supplying drugs or equipment? Did the authors declare a direct conflict of interest in relation to the study? Yes: Conflict of interest statement with no conflicts of interest. **Yes/No/Unclear**	Publication bias – Negative results will be less likely to be published if inappropriate influence of funder or biased companies occur

QoR: Quality of Reporting; MQ: Methodological Quality; RoB: Risk of Bias; TTR: Tumor take rate; NA: Not applicable

## Results

### Study selection

**Fig. 1:**
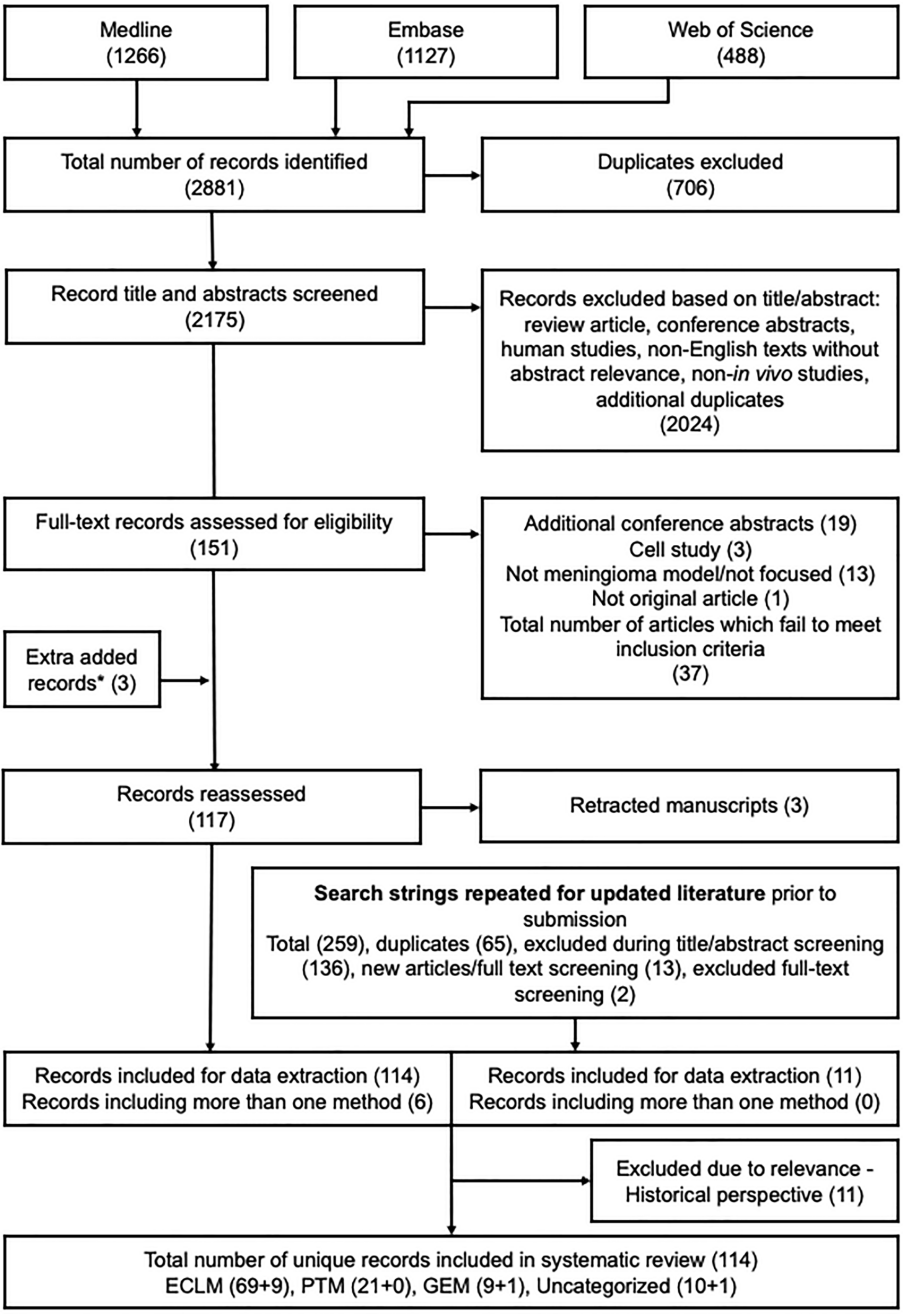
Flow chart. Established cell line models (ECLM), primary tumor models (PTM), genetically engineered models (GEM). *One record was initially screened negative on the abstract, and the other two studies were found through other literature (one as a historical perspective and the other as an ECLM)

The search strategy yielded 2175 unique studies, of which 151 were potentially eligible. The kappa index for the two reviewers at the screening stage of the first round was 0.9. One record was initially screened negative but was added through other literature alongside two other studies. Of the 117 studies that met the inclusion criteria, three were discarded due to retraction from the journal. The top-up search conducted in August 2022 led to the inclusion of 11 additional studies. We identified 78 studies on ECLM [6, 20–22, 24, 43–115], 21 studies on PTM [20, 24–26, 73, 106, 116–129], 10 studies on GEM [27–31, 130–134], and 11 studies categorized as “uncategorized” [30, 132, 135–143]. We decided to exclude studies older than 40 years due to lack of relevance. The PRISMA flow diagram for the study selection process is presented in Fig. 1. The original data extraction sheets for ECLM, PTM, GEM, and uncategorized studies are available in the Additional files 9, 10, 11, 12).

### Primary analysis

We conducted meta-analyses on the tumor take rate (TTR) for the five model groups. The overall TTR was 95% (95% CI 93–96%) for ECLM heterotopic models and 94% (92–96%) for ECLM orthotopic models, 82% (73–89%) for PTM heterotopic models and 53% (33–72%) for PTM orthotopic models, and 34% (26–43%) for GEM. While no statistical heterogeneity was found for the ECLM (I^2^ = 0%), the other meta-analyses showed substantial heterogeneity with I^2^ over 50%. Forest plots for these meta-analyses are available in the Additional file 13.

### Subgroup analyses

Table 2 shows the results of subgroup analyses of TTR against duration of incubation, number of cells, injection volume, cell line and WHO grade, where significant subgroup interactions were found for at least one of the model types. The full results of subgroup analyses, including forest plots, can be found in the Additional file 13.

**Table 2:** Subgroup analyses of tumor take rate against duration of incubation, number of cells, injection volume, cell line and WHO grade for the five model groups

Subgroups	ECLM orthotopic TTR (95% CI)	ECLM heterotopic TTR (95% CI)	PTM orthotopic TTR (95% CI)	PTM heterotopic TTR (95% CI)	GEM TTR (95% CI)
Duration of incubation					
0–14 days	97% (92–99%)	97% (93–99%)	NA	NA	NA
14–30 days	93% (85–97%)	94% (90–97%)	98% (89–100%)	94% (88–97%)	NA
31–99 days	100% (91–100%)	96% (93–98%)	87% (65–96%)	91% (80–96%)	46% (5–93%)
100–199 days	92% (70–98%)	100% (54–100%)	NA	75% (45–92%)	36% (20–56%)
200–499 days	NA	NA	8% (3%-21%)	65% (37–86%)	27% (20–35%)
Unknown	95% (92–97%)	97% (89–99%)	67% (29–91%)	34% (13–63%)	38% (20–60%)
Number of cells					
0–100	NA	NA	0% (0–46%)	NA	NA
101–1.000	NA	NA	86% (42–100%)	NA	NA
1.001–10.000	NA	NA	67% (22–96%)	50% (12–88%)	NA
10.001–100.000	97% (93–99%)	98% (91–100%)	15% (5–37%)	32% (3–88%)	NA
100.001–500.000	97% (94–99%)	97% (92–99%)	NA	NA	NA
500.001–1.000.000	96% (91–99%)	94% (85–97%)	91% (77–97%)	93% (49–99%)	NA
1.000.001–10.000.000	100% (48–100%)	97% (95–99%)	NA	94% (81–98%)	NA
> 10.000.000	NA	100% (66–100%)	NA	96% (86–99%)	NA
Unknown	89% (82–94%)	93% (83–97%)	NA	83% (73–90%)	NA
Injection volume (μl)					
0–1	91% (56–99%)	NA	NA	NA	NA
2–5	95% (93–97%)	NA	16% (5–40%)	NA	NA
6–10	98% (94–99%)	NA	86% (69–94%)	NA	NA
0–99	NA	100% (96–100%)	NA	97% (87–99%)	NA
100–250	NA	96% (93–98%)	NA	94% (69–99%)	NA
251–500	NA	95% (79–99%)	NA	88% (40–99%)	NA
501–1000	NA	NA	NA	76% (52–90%)	NA
Unknown	92% (82–97%)	96% (92–98%)	NA	83% (73–90%)	NA
Cell line					
IOMM-Lee	97% (95–98%)	94% (90–96%)	NA	NA	NA
CH-157	89% (81–94%)	97% (89–99%)	NA	NA	NA
BEN-MEN-1	97% (81–100%)	NA	NA	NA	NA
HBL52	NA	99% (93–100%)	NA	NA	NA
HKBMM	100% (86–100%)	93% (77–98%)	NA	NA	NA
SF4433	NA	98% (85–100%)	NA	NA	NA
SF3061	NA	100% (88–100%)	NA	NA	NA
F5	95% (79–99%)	100% (88–100%)	NA	NA	NA
KT21	95% (80–99%)	100% (29–100%)	NA	NA	NA
NCH93	NA	97% (80–100%)	NA	NA	NA
Me10T	100% (54–100%)	NA	NA	NA	NA
Me3TSC	100% (54–100%)	NA	NA	NA	NA
MN3	96% (79–99%)	NA	NA	NA	NA
WHO grade/grade					
Grade 1/benign	NA	NA	47% (17–79%)	88% (78–94%)	NA
Grade 2/atypical	NA	NA	36% (7–81%)	59% (9–95%)	NA
Grade 3/malignant	NA	NA	50% (18–82%)	75% (51–89%)	NA
Unknown	NA	NA	90% (68–99%)	93% (82–97%)	NA

NA: Not applicable. ECLM: Established cell line models. PTM: Primary patient-derived tumor models. GEM: Genetically engineered models

### Meningioma animal models

**Fig. 2:**
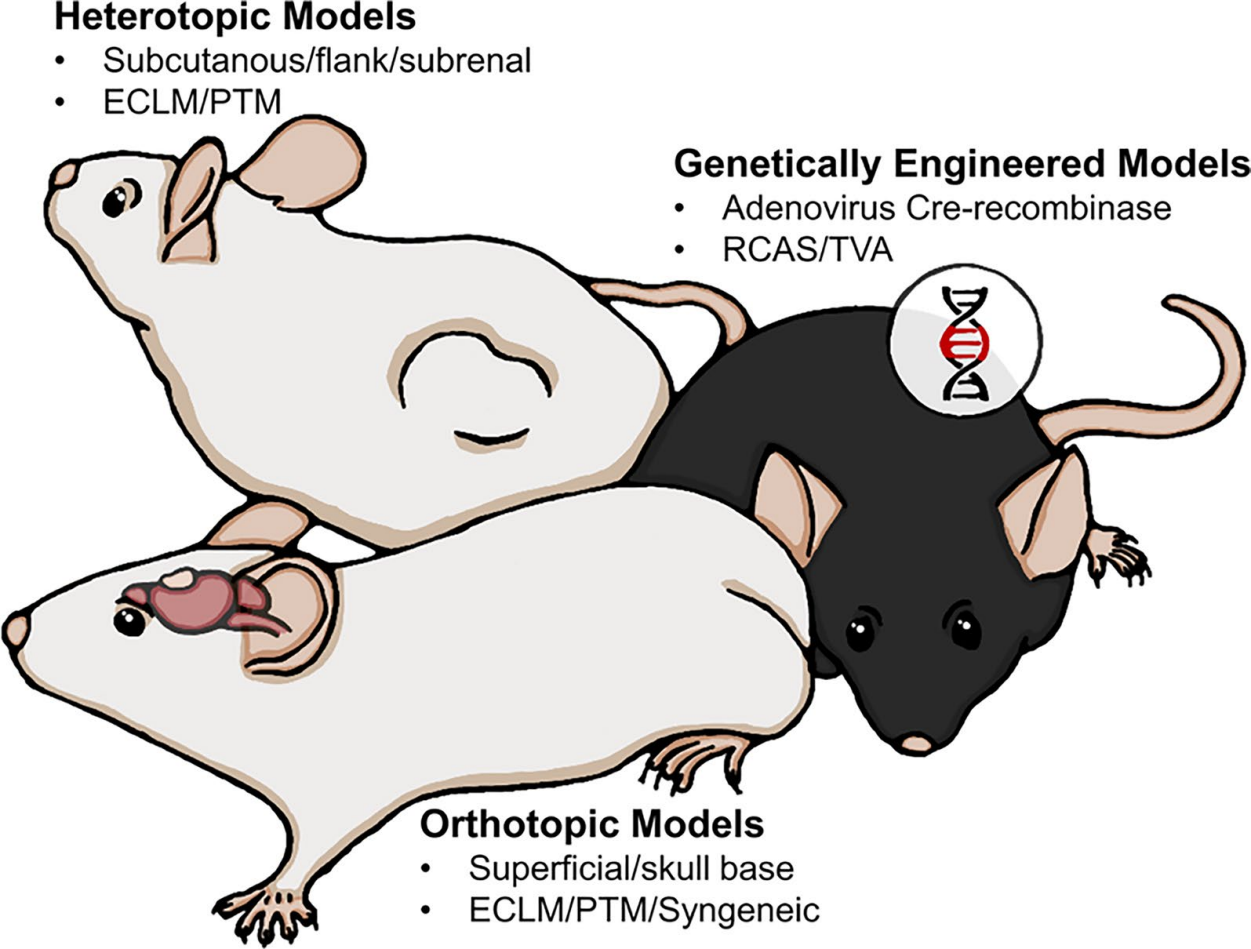
Overview of the most common model types. Both ECLM (established cell line models) and PTM (primary patient-derived tumor models) require immunocompromised hosts. PTM include cell injection models and whole tumor ‘PDX’ models. Syngeneic modelling is achievable in immunocompetent hosts. RCAS/TVA: Replication-competent avian leukosis virus splice-acceptor/tumor virus A. *Illustrator: Mikkel Schou Andersen*

There is a plethora of methods available for creating a meningioma animal model depending on the research question. Commonly used methods include xenografting cells (established/commercially available or primary patient-derived) or whole tumor pieces either orthotopically or heterotopically and the more recent genetically engineered models (Fig. 2).

### Patient-derived xenotransplantation and xenografts

Patient-derived xenotransplantation is the transplantation of tissue foreign to the host e.g., where meningioma tissue or cells derived from patients are transplanted into small animals such as immunocompromised mice. As a minimum, the animals need to have absent mature T-cells such as the BALB/c nude, Athymic nude, CD-1 nude, NMRI nude, NU/NU, and Swiss nude mice or the severe combined immune-deficient animals such as NSG, NRG and NOD SCID, and SCID mice that lack T cells and B-cells and an innate immune system [144]. Most studies use animals up to 10 weeks old, with some exceptions (12–15 [62], 10–22 [53], 7–11 [62], and 12–16 [25] weeks old).

The two main types of models used are the orthotopic models and the heterotopic models. An overview of these models is provided in Table 4 (for ECLM) and Table 5 (for PTM). Further details are available in the Additional files 9, 10, 11).

The orthotopic model requires implantation of material intracranially, most commonly through a burr hole in the frontal region of the skull (typically 1–3 mm anterior and 1–3 mm right of bregma) either superficially or at the skull base. All the reviewed orthotopic models inject between 0.5–10 μl of volume, with 2–10 μl being most common without noteworthy issues. Heterotopic models have a higher injection volume (commonly 100–500 μl), and a larger number of cells are needed to obtain a large tumor. Thus, while orthotopic models typically use 10^5^–10^6^ cells, most of the heterotopic models inject > 10^6^–10^7^ depending on the type of cells used. Since there are potential big differences in TTR between the immortalized cell line models and the primary patient-derived cell/tissue models, we have chosen to distinguish between the two in the sections below.

#### Established/commercially available cell line models (ECLM)

Meningioma established patient-derived cell lines have been used for decades for in vitro and in vivo research. Most commonly used is the *Nf2* + , malignant, IOMM-Lee, which was established from an intraosseous malignant meningioma from 1990 [22]. Other noteworthy cell lines include the *Nf2*^*−*^, benign BEN-MEN-1, which was *hTERT*- immortalized [21], the malignant *KT21* [100] with heterozygous loss of *Nf2*, and the malignant CH-157, whose origin remains a mystery [20, 145, 146]. Other cell lines have been produced for various purposes. Some of these have complex karyotypes such as KCI-MENG1 [105] and SF3061 [109], which is also *Nf2*^+^, or the MeTSC, which is *Nf2*^*−*^ [69]. Others have a simple karyotype such as SF4433 [147], and BEN-MEN-1 [20]. Table 3 shows all established/commercially available meningioma cell lines used in vivo including both the origin paper and papers in which the cell lines have been used*.*

**Table 3:** Overview of established/commercially available cell lines used in vivo

Cell line	Origin/location	Origin grade	Immortalization	Morphological characterization and traits	CHM in vivo	Genomic/cytogenetic characteristics	NoP,^a^	Origin paper
IOMM-Lee	61 yo man/Frontal, parietal	Malignant	Spontaneous—long term cultured	Intraosseous malignant meningioma Infiltrates brain tissue [20], bone and subcutaneous tissue	Vimentin + , GFAP-, S100b( +), high Ki67 (30%) [22, 44, 53, 59, 81], high MIB-1 (211.0) [20], *SSTR2A* + (in vitro) [148]	*Nf2* + [149], chromosomal abnormalities X, − Y, − 1, *add *(2)*(p11.2)*, *add *(5)*(p13)*, add (6)(p13),i(7)(p10) × 2, add(9)(q21), add(12)(q21),*-17*, *add(14)(q13)*, add(19), *add(20)*, and + 2–4 mar[p20] [20]	49 [20, 22, 24, 43–88]	Lee 1990 [22]
BEN-MEN-1	68 yo woman/Parietal, falx	Benign WHO grade 1	hTERT	Meningothelial	EMA + , Vimentin + , GFAP-, PR-, ER-, Ki67 (< 1%)	*NF2-*, (45, XX,-22) [149]	6 [21, 55, 89–92]	Püttmann et al. 2005 [21]
CH-157	41 yo woman [145], 59 yo [146], 55 yo [20]/ND	Unknown (cell line malignant)	Spontaneous	Not tissue infiltration, but central core necrosis [20]	Vimentin + , EMA + , high MIB-1 (190.6) [20]	*NF2-* [149], X, add (X)(p11.2), − X, add(1)(q21), add(1)(p13), + 2, + 3, + 5, − 1, i(8)(q10) × 2, + 8, add(11)(p11.2), + 11, + 12, add(14)(p11.2), − 15, i(15)(q10), − 16, + 16, -17, − 18, + 20, − 22, + 4-9mar[cp10] [20]	13 [6, 20, 43, 67, 72, 85, 88, 93–98]	Tsai et al. 1995 [145]
KT21MG1/ KT21	47 yo woman/Falx	Malignant	Spontaneous c-myc amplification—long term cultured	Epithelial cell like morphology	Vimentin + , GFAP-	Heterozygote loss of Chromosome 22	6 [59, 61, 90, 99–101]	Tanaka et al. 1989 [100]
HKBMM	52 yo woman/	Malignant, WHO grade 3	Spontaneous—Long term cultured	Round and spindle-shaped cells showing neoplastic and pleomorphic and abundant mitosis	Desmin + , PKK1 + , Vimentin + , EMA-, S-100-	‘Varying widely and showed aneuploidy’	4 [84, 102–104]	Ishiwata et al. 2004 [104]
F5	ND/ND	Malignant	ND	Invades skull and subcutaneous tissue	ND	ND	3 [81, 106, 107]	Yazaki et al. 1995 [106]
NCH93	64 yo man/parieto-occipital	Anaplastic, Grade 3	ND/spontaneous	Anaplastic	EMA + , 50% KI67	*NF2-*	3 [110, 111, 115]	Jungwirth et al. 2019 [110]
HBL52	47 yo woman/Optic canal	Benign grade 1	ND/spontaneous	Transitional meningioma	In vitro*:* EMA + , vimentin + , GFAP-,	*NF2* + , missense mutation *TRA7* – no broad copy number alterations[149]	1 [112]	Akat et al. 2003 [150]
MN3	ND/ND	Recurrent Malignant WHO grade 3	Spontaneous—Serial passages in vivo	fibroblastic meningioma “whorl formations” by spindle-shaped tumor cells. Cells with nuclear atypia	Vimentin + , EMA-, Nestin +	*NF2-*, missense mutation *ALK, PTCH1*	2 [113, 114]	Nigim et al. 2016 [113]
MN8	ND/Ventricle	Recurrent anaplastic WHO grade 3	Spontaneous—Serial passages in vivo	ND	Vimentin + , EMA + , high ki-67	*NF2-*	1 [114]	Nigim et al. 2019 [114]
SF4433	ND/ND	Benign (Grade 1, 2000 WHO)	E6/E7 + hTERT	ND	Vimentin +	*NF2* + *,* no chromosomal abnormalities	2 [108, 109]	Baia et al. 2006 [147]
SF3061	ND/ND	Malignant (Grade 3, 2000 WHO)	hTERT	ND	Vimentin +	*NF2* + *,* losses: 9p24-p21; 11q23-qtel; 13q12-q21;17p	1 [109]	Baia et al. 2006 [147]
KCI-MENG1-LP	46 yo woman/Olfactory	Benign grade 1	Spontaneous—Low passage (< 10. High telomerase activity)	Necrotic core, intermingled brain-tumor interface, heterogenous cell morphology, spindle and round cells	EMA + , N-Cadherin + , Vimentin + , PR( +), high Ki-67	*NF2* + 64–66 chromosomes, XX (two clones: Clone 1 complex, clone 2: t(2;13)(q37;q22) and t(4;7)(q21;p13) and 45, XX	1 [105]	Michelhaugh et al. 2015 [105]
KCI-MENG1-HP	46 yo woman/Olfactory	Benign grade 1	Spontaneous—High passage (< 72)	Heterogenous cell morphology spindle and round	EMA + , N-Cadherin + , Vimentin + , PR-, very high Ki-67	*NF2* + 64–66 chromosomes, XX (clone 1 only: see above—very complex karyotype see paper)	1 [105]	Michelhaugh et al. 2015 [105]
Me3TSC	ND/ND	Benign, Grade 1	hTERT	Spindled to epithelioid cells with monomorphic round to oval nuclei. Focal whorls and microcalcifications Nuclei displayed pleomorphism and had visible nucleoli	Vimentin + , cytokeratin + , S-100 + , EMA-, PR-(primary EMA +)	*NF2-* (complex karyotype) 45,XX,t(1;5)(p?36.1;q?13),del(9) (p13,del(11)(p14);-22	1 [69]	Cargioli et al. 2007 [69]
Me10T	ND/ND	Benign, Grade 1	hTERT	See above	Vimentin + , cytokeratin + , S-100 + , EMA-, PR- (primary EMA +)	*NF2-* (45,XX,-22)	1 [69]	Cargioli et al. 2007 [69]

ND: Not described, NoP: Number of papers using cell type for in vivo purposes, ^a^some papers use more than one cell line, CHM in vivo: Common histological markers in vivo, yo: years old, EMA: Epithelial membrane antigen, PR: Progesterone receptor, GFAP: Glial fibrillary acidic protein, ER: Estrogen receptor

Over the last 40 years of research, about 70% of meningioma studies have used ECLM (Table 4). The most commonly used, IOMM-Lee, shows a high tumor take rate in subgroup analysis for both orthotopic models 87% (95% CI 95–98%) and heterotopic models 94% (90–96%), and it shows median survival of 10–27 days in orthotopic models, depending on the number of cells. In general, 10^4^–2.5 × 10^5^ cells are needed for orthotopic and heterotopic ECLM (Table 4). CH-157 has a TTR of 89% (81–94%) in orthotopic models and 97% (90–96%) in heterotopic models and a similar median survival of 12–24 days in orthotopic models with cell concentrations of 10^4^–10^6^ cells. The most used benign cell type is the BEN-MEN-1, which has only been studied in orthotopic models (TTR of 97% (81–100%); using 0.5–1.0 × 10^6^ cells, researchers have created a long-term model (Ki67 < 1%) up to 180 days [21]. (For further details, see Additional file 9).

**Table 4:** Orthotopic and heterotopic models using established cell lines (ECLM)

Cell line/WHO-gr	ToA mice	Age (w)	NoC/IV	DoI (d)	TTR %(pooled animals)	IVoG	NIVoG	PuCL
Orthotopic models								
IOMM-Lee/malignant	Athymic nu/nu [24, 49, 54, 66, 70, 74, 77, 80], SCID [44, 55], Nude [45, 68, 74, 78, 82, 83], Swiss nude [47, 53, 59, 61, 62, 79, 81], BALB/c [50, 58, 84], NOD(shi-SCID,IL-2Rgamma(null,c)(NOG) [60], CD1 [67], NCr-Foxn1 (nu) [85]	3 [67], 4–10 [24, 44, 45, 49, 50, 54, 55, 58–61, 66, 74, 77–79, 81–85], > 9 [47], 12–15 [62], 10–22 [53], ND [68, 70, 75, 80]	(10^4^) [49], (5 × 10^4^) [60, 66, 83], (10^5^) [50, 62, 67, 78, 82], (1.5 × 10^5^) [58], (2 × 10^5^) [85] (2.5 × 10^5^) [47, 53, 54, 59, 61, 79, 83, 84], (4 × 10^5^) [45], (5 × 10^5^) [66–68, 70, 75], (10^6^) [24, 44, 74, 77, 81], (3 × 10^6^) [66]/(0.5) [66, 83], (1) [66], (2) [62, 84] (2.5) [47, 53, 79, 83], (3) [50, 58, 60, 66, 67, 77], (5) [54, 59, 61, 66, 81], (3–10) [44], (10) [24, 68, 74, 78, 82] CN-ND [55, 80], IV-ND [45, 49, 55, 68, 70, 75, 80, 85]	CD (4–12) [24], (5) [60], (9) [61], (10) [59, 60], (11) [53], (14) [58, 60, 62, 67, 74, 81], (21) [78], (28) [75] SS (10) [60, 66], (11) [85], (12) [66], (13) [77], (15) [53], (17) [47, 66], (20) [66, 79], (21) [49, 84], (23) [50], (27) [45], 15–21 [83] ND [44, 54, 55, 68, 70, 80, 82]	100% (504/504) [24, 45, 50, 53, 58–62, 66–68, 70, 75, 77–79, 81, 82, 84] ND [44, 47, 49, 54, 55, 74, 80, 83, 85]	Histology [24, 44, 45, 47, 50, 53, 54, 60–62, 66–68, 70, 74, 75, 77–79, 81, 82, 84, 85] IHC [24, 44, 45, 53, 58–60, 66, 68, 70, 77, 78, 80, 84]	BLI [45, 49, 50, 66, 67, 85] MRI [44, 47, 53, 55, 59, 61, 62, 67, 79, 81, 83] FI [58, 60]	29 [24, 44, 45, 47, 49, 50, 53, 54, 58–62, 66–68, 70, 74, 75, 77–85] ^a^[55]
BEN-MEN-1/benign	NSG [89, 92], SCID [55, 90, 91], CD1 [21]	6–12 [21, 55, 89–92]	(0.5–1.0 × 10^6^) [91], (10^6^) [21, 89, 90]/3–5 [21, 89–92] CN-ND [55]	CD (35 [90], 98 [89, 92], 107 [21], 180 [91]) ND [55]	100% (45/45) [21, 92] ND [55, 89–91]	Histology [21, 55, 90–92] IHC [21, 90–92]	BLI [89–92] MRI [55, 91]	6 [21, 55, 89–92]
CH-157/malignant	NCr-Foxn1(nu) [85], NSG [93], CD1 [67, 94], nude [43, 98]	3 [67, 94] 4–6 [43, 85, 93, 98]	(10^4^) [67], (10^5^) [43, 67, 94], (2 × 10^4^) [93], (2 × 10^5^) [85], (5 × 10^5^) [67], (10^6^) [67] (5 × 10^4^–5 × 10^5^) [98], (10^5^–10^6^) [98] /(3–8) [67, 93, 94, 98], IV-ND [43, 85]	CD 14 [43], 15 [93], 20 [94], 30 [98] SS 12–15 [85], 16–24 [67]	100% (66/66) [93, 94] 91% (20/22) [98] 90% (45/50) [67] 79% (15/19) [98] ND [43, 85]	Histology [43, 67, 85, 93, 94, 98] IHC [43, 67, 94]	BLI [43, 67, 85, 93, 94] MRI [98]	6 [43, 67, 85, 93, 94, 98]
KT21MG1/malignant	Swiss nude [59, 61], NMRI nu/nu [99], athymic [101], SCID [90]	5–6 [99, 101] 8–10 [59, 61, 90]	5 × 10^4^ [101]; 2.5 × 10^5^ [59, 61]; 5 × 10^5^ [90]; 10^6^ [99] /0.5 [101], 5 [59, 61, 90]; IV-ND [99]	CD 10 [99], 17 [61], 21 [59], 42 [90] SS 19 [101]	100% (50/50) [59, 61, 99] ND [90, 101]	Histology [90, 99] IHC [59, 90, 99, 101]	BLI [90] MRI [59, 61]	5 [59, 61, 90, 99, 101]
F5	NOD/SCID [107], Swiss nude [81], BALC/-nu/nu [106]	6–8 [81, 107]	(2.5 × 10^5^) [107], (10^6^) [81], WT [106]/(5) [81], IV-ND [107]	CD 14 [107], 29 [81] SS (29) [106]	100% (15/15) [81], 94% (15/16) [106], ND [107]	Histology [81, 106, 107] IHC [106, 107]	MRI [81, 107]	3 [81, 106, 107]
MN3	SCID [113, 114]	7–8 [113, 114]	(7.5 × 10^4^) [113, 114]/(3) [113, 114]	SS 92 [114] ND [113]	100% (25/25) [113, 114]	Histology [113, 114] IHC [113, 114]	–	2 [113, 114]
MN8	SCID [114]	7–8 [114]	(5 × 10^4^)(114)/(3) [114]	ND [114]	ND [114]	Histology [114] IHC [114]	–	1 [114]
HKBMM	BALB/C-nu/nu [84]	6–8 [84]	(2.5 × 10^5^) [84]/(2) [84]	SS 27 [84]	100% (24/24) [84]	Histology [84] IHC [84]	–	1 [84]
Me10T Me3TSC	Athymic [69]	4 [69]	(10^6^) [69]/(5) [69]	CD (112) [69]	100% (6/6) [69] Me10T 100% (6/6) [69] Me3TSC	Histology [69] IHC [69]	–	1 [69]
Heterotopic models								
IOMM-Lee/malignant	Athymic nu/nu [49, 57, 71, 75, 76], BALB/c-nu [22, 46, 48, 51, 52, 56, 84], SCID [55, 63], Swiss nude [59, 61, 62, 83], nude [45, 64, 65, 86, 88], CD1 [20, 72, 73], C57BL/6 [87]	3 [20, 72, 73], 4–6 [46, 51, 52, 56, 71, 87, 88], 6 [45, 55], 6–8 [49, 64, 65, 76, 84, 86], 8 [22, 48, 58], 8–10 [59, 61, 83], 7–11 [62], ND [57, 63, 75]	(2 × 10^4^) [46], (1.5 × 10^5^) [58], (5 × 10^5^) [20, 46, 72], (10^6^) [57, 63, 71, 73, 76], (1.5 × 10^6^) [20], (2 × 10^6^) [45, 86, 88], (3 × 10^6^) [59, 61, 62, 83], (4 × 10^6^) [64], (5 × 10^6^) [48, 49, 52, 65, 75, 84], (10^7^) [22, 55, 56, 87], (5 × 10^7^) [51]/(3) [58] (100) [46, 48, 55, 58, 59, 61, 63, 72, 73, 75, 76, 83, 84, 86, 87], (200) [22, 51, 88], (500) [20], IV-ND [45, 49, 52, 56, 57, 62, 64, 65, 71]	CD 12 [52], 14 [58, 62, 76], 16 [84], 17 [59], 21 [57, 61, 65, 83], 25 [64, 86], 26 [71], 28 [46, 48, 75, 88], 30 [56], 31 [49], 34 [51], 35 [45], 43 [72], 45 [87], 56 [20, 73] ND [22, 55]	100% (432/432) [22, 45, 46, 48, 51, 52, 57–59, 61, 62, 64, 65, 71–73, 76, 83, 86, 87] 95% (19/20) [20] 87% (13/15) [63] 77% (23/30) [56] ND [49, 55, 75, 84, 88]	Histology [20, 22, 45, 49, 51, 52, 63, 65, 72, 73, 75] IHC [20, 22, 45, 46, 48, 49, 51, 52, 58, 59, 61, 63–65, 71–73, 86–88]	Caliper [45, 49, 51, 52, 55–59, 61, 62, 64, 71, 72, 76, 83, 84, 86, 87] FI [58]	28 [20, 22, 45, 46, 48, 49, 51, 52, 55–59, 61–65, 71–73, 75, 76, 83, 86, 87] ^a^[84, 88]
CH-157/malignant	NU/NU [6, 95, 96], CD1 [20, 72], BALB/c-nu [97],	3 [20, 72], 5–6 [6] ND [95–97]	(5 × 10^5^) [72], (10^6^–1.25 × 10^6^) [20], (1.5 × 10^6^) [95–97], (3 × 10^6^) [6] /(100–500) [20, 72, 95, 97]; IV-ND [6, 96]	CD 28 [6], 30 [95], 43 [72], OC 43 [20] ND [96, 97]	100% (47/47) [20, 72, 95, 96] ND [6, 97]	Histology [20, 72, 97] IHC [6, 20, 72, 95, 97]	Caliper [20, 72] PET [95, 96] FI [97]	7 [6, 20, 72, 95–97] ^a^[88]
HKBMM	BALB/cAJcl-nu/nu [102], Nude [103], BALB/c-nu [84, 104]	4–6 [103], 6–9 [84, 102, 104]	(5 × 10^5^) [102], (10^6^) [102], (5 × 10^5^) [84], 7 × 10^6^) [103], (10^7^) [104]/(100) [84], (200) [102, 103], IV-ND [104]	CD 16 [84], 49 [103], 56 [104], 140 [102] ND [102]	100% (28/28) [102–104]	Histology [103, 104] IHC [103, 104]	Caliper [84, 102]	4 [102–104] ^a^[84]
NCH93	NMRI/nu [110, 111, 115]	5–6 [110, 111, 115]	(10^6^) [110], (4 × 10^6^) [111, 115]/(100) [110, 111], (200) [115]	SS 15 [111], 21 [115], 49 [110]	100% (44/44) [110, 111, 115]	Histology [110, 111, 115] IHC [110, 111, 115]	Caliper [110, 111, 115]	3 [110, 111, 115]
SF4433	Athymic [108, 109]	5 [108], 6 [109]	(2 × 10^5^) [108, 109]/(100) [108, 109]	CD 14 [109], 17 [108]	100% (40/40) [108, 109]	-	BLI [108, 109]	2 [108, 109]
SF3061	Athymic [109]	6 [109]	(2 × 10^6^) [109]/(100) [109]	CD 11 [109]	100% (30/30) [109]	-	Caliper [109]	1 [109]
KT21MG1/malignant	ICR nu/nu [100]	4–5 [100]	8 × 10^6^ [100]/IV-ND [100]	CD 90 [100]	100% (3/3) [100]	Histology [100] IHC [100]	–	1 [100]
F5	BALC/c [106]	6 [106]	WT [106]	SS 28 [106]	100% (40/40) [106]	IHC [106]	–	1 [106]
HBL52	BALB/c [112]	7 [112]	(10^5^) [112]/(200) [112]	CC 49 [112] SS (27) [112]	100% (96/96) [112]	IHC [112]	Caliper [112]	1 [112]
KCI-MENG1-LP/HP	SCID/NCr [105]	ND [105]	ND [105]	Serial transplantations [105]	ND [105]	Histology(105) IHC [105]	–	1 [105]

ToA: Type of animal, NoC/IV: Number of cells/injection volume (μl), DoI: Duration of incubation, TTR: Tumor take rate, w: weeks, d: days, ND: Not described to a degree of certainty/Not described at all, PuCL: Papers using the cell line, IVoG: Validation of growth, NIVoG: Non-invasive validation of growth, BLI: Bioluminescence, SS: Survival study—number of days 50% dead (control animals) or human endpoint met, CD: Chosen day of death, OC: Other causes for termination, FI: Fluorescence imaging

^a^Uncertain use of cells in model, WT (whole tumor from original paper)

#### Primary patient-derived tumor models (PTM)

The non-immortalized patient-derived models are comprised of all studies describing implantation either straight from surgery as cells or whole tumor pieces [25, 106, 122, 125, 127] or after fewer than 10 passages (usually after 3–6 passages) [24, 26, 67, 72, 118–121, 123, 124]. For orthotopic models, subgroup analyses (Table 2) showed varying TTRs ≤ 50% with a benign model TTR of 47% (95% CI 17–79%), atypical tumor model TTR of 36% (7–81%), and malignant tumor model TTR of 50% (18–82%) (see Table 5 for individual TTR). McCutcheon et al. [24] tested various durations for different WHO tumor grades and showed succesful tumor take time of 21–56 days for benign, 14–28 days for atypical, and 4–12 for malignant. Zhang et al. [25] performed survival studies on atypical tumors up to 240 days and malignant tumors up to 160 days. In general, successful benign models required 10^6^ cells, atypical models 10^5^–10^6^ cells, and malignant down to 10^3^ cells.

**Table 5:** Patient-derived primary tumor meningioma models in vivo

Grade	Type of animal	Age (w)	Number of cells/injection volume (μl)	Duration of incubation (d)	Tumor take rate % (pooled animals)	IVoG	NIVoG	Papers
Orthotopic models								
Benign/WHO-Grade 1	Nu/nu [119], Rag2SCID [25], athymic nu/nu [24],	4–5 [26, 119], 6 [24], 12–16 [25]	(10^5^) [25], (10^6^) [24, 26, 119] / (2) [25], (10) [24, 26, 119]	CC 21 [24], 42 [24], 56 [24], 90 [26, 119] NA [25]	100% (34/34) [119], 93% (27/29) [26], 69% (56/81) [24], 0% (0/30) [25]	Histology [24, 26, 119] IHC [24, 26, 119]	–	4 [24–26, 119] ^a^ [25, 26, 119]
Atypical/WHO-Grade 2	Rag2SCID [25], athymic nu/nu [24]	6 [24], 12–16 [25]	(10^5^) [25], (10^6^) [24] / (2) [25], (10) [24]	CC 14 [24], 21 [24], 28 [24], 360 [25] SS (240) [25]	100% (58/58) [24, 25], 70% (7/10) [25], 0% (0/70) [25]	Histology [24, 25], IHC [25], RNA sequence [25]	MRI [25]	2 [24, 25] ^a^ [25]
Malignant/WHO-grade 3	Rag2SCID [25], BALB/c-nu [121], athymic nu/nu [24]	6 [24], 12–16 [25], ND [121]	(10^2^) [121], (10^3^) [121], (10^4^) [121], (10^5^) [25, 121], (10^6^) [24, 121] / (2) [25], (10) [24, 121]	CC 4 [24], 8 [24], 12 [24], 360 [25] SS (160) [25] ND [121]	100% (12/12) [121], 90% (9/10) [25], 86% (6/7) [121], 67% (4/6) [121], 25% (1/4) [25] 0% (0/38) [25, 121], ND [24]	Histology [24, 25, 121], IHC [24, 25, 121], RNA sequence [25]	MRI [25]	3 [24, 25, 121] ^a^ [25]
Unknown/uncertain	Nu/nu [116]	6 [116]	(10^6^) [116]/(2) [116]	CC (90) [116]	90% (18/20) [116]	–	MRI [116]	1 [116]
Heterotopic models								
Benign/WHO-grade 1	CD1 [20, 72, 73], nude [120], C57B1/6 J-nu [122], CD1 athymic BALB/c [123], Swiss nu/nu/Ncr [124], BALB/c-nu [106, 126, 128]	2 [123], 3 [20, 72, 73], 4 [126], 6 [106], 8–10 [124], ND [120, 122, 128]	(10^5^) [20], (10^6^) [73, 123], (1.5 × 10^6^) [124], (1.7 × 10^6^) [20], (5 × 10^6^) [72], (10^7^) [120, 122], (1.1 × 10^7^) [20], (1.6 × 10^7^) [20], (5 × 10^7^) [126]/(10) [126], (100–150) [72], (500) [20, 123], (800)[124], WT[106, 122, 128], SR[106, 126], IV-ND[106, 120, 122]	CC 28 [126], 35 [20], 43 [72], 51 [20], 56 [106, 124], 84[120], 90 [128], 96 [20], 150 [73, 123], 180 [122], 270 [122], 330 [122]	100% (199/199) [72, 106, 123, 124, 126, 128], 85% (17/20) [120], 76% (35/46) [122], 75% (15/20) [20], ND [73]	Histology [20, 72, 73, 122, 123, 126] IHC [20, 72, 73, 120, 122, 123] Measured surgicals [106]	Caliper [120, 122–124, 128]	10 [20, 72, 73, 106, 120, 122–124, 126, 128]
Atypical/WHO-Grade 2	C57B1/6 J-nu [122], Swiss nu/nu/Ncr [124]	8–10 [124], ND [122]	(1.5 × 10^6^) [124], (10^7^) [122], WT [122]/IV-ND [122]	CC 56 [124], 180 [122]	100% (8/8) [124] 33% (4/12) [122]	Histology [122] IHC [122]	Caliper [122, 124]	2 [122, 124]
Malignant/WHO-Grade 3	BALB/c-nu [106, 118, 121, 129], Swiss nu/nu/Ncr [124]	4–5 [118], 6–10 [106, 124, 129], ND [121]	(10^4^) [121], (10^5^) [121], (10^6^) [118, 121], (1.5 × 10^6^) [124], (2 × 10^6^) [129]/(1000) [121], (250) [118], WT [106], SR [106, 129], IV-ND [129]	CC 30 [118], 35 [106], 42 [129], 56 [124], ND [121]	100% (43/43) [106, 118, 124], 67% (4/6) [121], 60% (3/5) [121], 50% (3/6) [121], ND [129]	Histology [118, 121] IHC [118, 121] Measured surgically [106, 129]	Caliper [118, 121, 124]	5 [106, 118, 121, 124, 129]
Unknown/uncertain	BALB/c-nu [117, 125], CD1 [127]	3 [125], 6–10 [117], ND [127]	(5 × 10^6^–10^7^) [127], WT [117, 125, 127], SR [117, 127], SG [125]	CC 28 [117], 30 [125] ND [127]	100% (78/78) [117, 125], 83% (?/?) [127], 75% (?/?) [127]	Histology [125, 127] Electron microscopy [125]	Caliper-PM [117]	3 [117, 125, 127] ^a^[127]

WT: Whole tumor pieces; SR: Subrenal capsule, SG: Subgalea, Caliper-PM: Caliper post-mortem, ND: Not fully described to a certain degree

^a^Aspects regarding take and/or duration are difficult to assess, See separate papers/Additional file 10: Table S10

For heterotopic models, subgroup analyses showed TTRs for benign tumor models of 88% (95% CI 78–94%), atypical tumor models 59% (9–95%), and malignant tumor models 75% (51–89%) (Table 2). Benign models have been more often used and show more consistent results with 10^5^–5 × 10^7^ cells and duration of incubation ranging from one month to almost one year. Duration of incubation for atypical tumors is up to 180 days [122] while that for malignant tumors is significantly shorter ranging from 30 to 56 days [106, 118, 124, 129].

Some studies in both the orthotopic and heterotopic groups do not describe WHO grade or tumor subtype, and thus valuable information is difficult to obtain [116, 117, 125, 127] (for further details, see Additional file 10).

##### Strengths and weaknesses of xenografting to immune-incompetent/compromised animals

The advantages of xenografting material or cells into immunocompromised animals are the lower cost and higher availability compared to for instance GEM. The strength of the orthotopic models is that tumors grow in the appropriate microenvironment (without taking alterations of the immune system into account), making them suitable for drug testing. The heterotopic models are performed outside of the central nervous tissue most commonly via a flank/subcutaneous injection model and are the easiest model to set up and perform. Subcutaneous injections of meningioma cells have been successful both with and without the use of Matrigel (which forms a solid gel at 37 °C to keep cells close together during tumor development [170]) or fibrin clots (for further details, see Additional file 9, 10, 11, 12). There are concerns, however, that Matrigel enhances tumorigenicity or even modulates characteristics of the original tumor. It may even increase drug resistance in vivo [171] and might transform pre-malignant to malignant cells [172]. The translatability of heterotopic models is debatable [32].

Meningioma established cell lines have been used for decades for in vitro and in vivo research. Some cell lines (such as IOMM-Lee, BEN-MEN-1, and CH-157) have the advantage of being thoroughly characterized on every level from histology to genetic profiles [20, 149]. The use of established cell lines produces consistent and homogeneous results across studies. Using established (immortalized) cell lines also negates the great issue of senescence that is often experienced in patient-derived primary cells (non-immortalized). A drawback of immortalized cell lines is that they are very far away from human meningioma conditions, e.g. IOMM-Lee’s complex karyotype probably due to long-term culturing [114]. ECLMs do not display normal meningioma pathology, disease nature, or heterogeneity, which in general makes them unsuitable for pharmaceutical studies.

In contrast to established cell lines, the primary patient-derived non-immortalized models show varying degree of tumor take within and between studies. Zhang et al*.* [25] reported TTRs ranging from 0% for most of the tumors to 90% (for a malignant tumor), and none of their xenografted benign cell lines could be detected even after a full year. Other studies also show inconsistency both inter-and intratumorally [24, 67]. Our own group has experienced similar issues with verified benign meningiomas *(unpublished data)*. Despite these obstacles, primary patient-derived non-immortalized models display inter-patient tumor variability more accurately for possible targeted personalized treatment.

The major limitation to xenotransplant models is that they must be performed in immunocompromised animals, thereby circumventing natural response by the adaptive immune system (whether this is anti- or pro-tumorigenic) [173, 174]. In addition, the heterotopic animal models do not provide the correct microenvironment for the meningioma cells and can alter the way they grow and express cell markers. Finally, the tumor development is not de novo, meaning that xenograft studies are not useful for studying tumor origins.

### Genetically engineered models (GEM)

GEM are based on mice that have undergone genome alterations through various genetic engineering techniques. There are multiple ways of achieving the desired genetic lesions. The following approaches have been used in meningioma research: The Cre-*loxP* system, which utilizes Cre-Lox recombination that can produce deletions and insertions at specific sites in the DNA. The DNA modification can be triggered by an external stimulus (e.g. recombinant adenovirus—AdCre) or be cell-type specific (i.e. is ‘conditional’). The alteration is performed by the splicing of previously inserted *LoxP* DNA sites using the enzyme Cre recombinase [151]. And the RCAS/TVA system, which utilizes retroviral infection via vectors that can only infect cells expressing the corresponding receptor TVA. The possibility of cloning the TVA receptor gene in mammalian cells has led to the creation of TVA-expressing transgenic mice [152]. RCAS is the vector and derives from the Rous sarcoma virus A [153]. The technology utilizes transfection of embryonic chicken fibroblast cell line DF-1 with the RCAS vectors, which then target ectopic TVA on pre-specified cells. The RCAS/TVA system is another example of a ‘conditional knockout’ system. In contrast to the Cre-*loxP* system, the RCAS/TVA system allows for simultaneous introduction of several genes of interest in the same cell [152].

As a group, meningiomas contain a plethora of DNA mutations depending on WHO grade and tumor location. Mutations in *Nf2* [154], *TRAF7, KLF4, AKT1,* and *SMO* are present in approximately 80% of sporadic meningiomas [3, 155]. Especially the rare genetic disorder neurofibromatosis type 2 (*Nf2*) at q22 predisposes to meningiomas of which approximately 50% display alteration of the tumor suppressor [155–157]. Table 6 displays all lesions and outcomes of studies involving GEM (for further details, see Additional file 11). Only a few genes have been studied, mostly the Nf2 gene [27, 28, 30, 31, 130].

**Table 6:** Genetically engineered models (GEM) used in meningioma research

Genetically Engineered Models								
Genetic lesion (mice)	Method of gaining lesion	Activation	Duration of incubation	Tumor Take Rate % (pooled animals)	Type of meningioma	Tumor take non-meningiomas/other pathological findings	Validation/verification	#
Nf2^(flox2/flox2)^	Conditional knockout. AdCre injection 3μl (3 × 10^8^ pfu) [27, 31, 130], (5 × 10^10^–1 × 10^11^ pfu) [30] subdural frontally [27, 30, 130] and transorbitally [27]	Injection: 2–3 day neonates (PN-2–3) [27, 28, 30, 130], PN1 [31]	CC 360 [130], 450 [28], 117 [30], 339 [30] SS 330 [27], 420 [27]	44% (24/55) [30], 30% (9/30) [27], 16% (4/25) [31], 12,5% (9/72) [28], 6% (1/18) [130]	Transitional [27, 28], meningothelial [27, 28, 30, 31], fibroblastic [27, 28, 30, 31], psammomatous [28] —benign [27, 28, 31], Grade 1 [30], ND [130]	SCH 10% (3/30) [27], osteoma 51% (80/157) [27, 28, 30], liver tumor 17% (26/157) [27, 28, 30], osteosarcoma 3% (1/30) [27], hydrocephalus 34% (61/182) [27, 28, 30, 31], ND or 0% (0/43) [31, 130]	Histology [27, 28, 30, 31], IHC (PGDS) [28], MRI [28, 30], electron microscopy [28, 30], ND [130]	5 [27, 28, 30, 31, 130]
Ptprj^(−/−)^	Ptprj(^−/−^) mice [130]	–	CC 360 [130]	0% (0/6) [130]	–	0% (0/6) [130]	–	1 [130]
Ptprj^(−/−);^ Nf2^(flox2/flox2)^	Conditional knockout AdCre injection 3 μl (3 × 10^8^ pfu) subdural frontally [130]	Injection: PN2-3 [130]	CC 360 [130]	25% (11/44) [130]	‘Typical meningioma’ whorls and psammoma bodies – Benign in appearance [130]	0% (0/44) [130]	Histology [130] IHC (Merlin, absent in Nf2 neg tumors) [130]	1 [130]
Nf2^(flox2/flox2)^; p53^(±)^	Conditional knockout AdCre injection 3 μl (3 × 10^8^ pfu) subdural frontally [27] and transorbitally [27]	Injection: PN2-3 [27]	SS 165 [27]	12% (4/33) [27]	Transitional, meningothelial, fibroblastic—benign [27]	MPNST 3% (1/33) [27], SCH 3% (1/33) [27], osteoma 64% (21/33) [27], sarcoma 85% (28/33) [27], osteosarcoma 6% (2/33) [27], liver tumor 12% (4/33) [27], pituitary adenoma 3% (1/33) [27], hydrocephalus 45% (15/33) [27]	Histology [27]	1 [27]
Nf2^(flox2/flox2)^; p16(ink4a)^(−/−)^	Conditional knockout AdCre injection 3 μl (3 × 10^8^ pfu) subdural frontally [28] and transorbitally [28]	Injection: PN2 [28]	CC 450 [28]	37% (10/27) [28]	Meningothelial [28], transitional [28], psammomatous [28] or fibroblastic [28]—Benign [28] 2/10 atypical features (prominent nucleoli, crowded cells) [28]	Osteoma 78% (21/27) [28], Liver tumor 19% (5/27) [28], hydrocephalus 56% (15/27) [28]	Histology [28], IHC (PGDS) [28], MRI [28], electron microscopy [28]	1 [28]
Nf2^(flox2/flox2)^;ink4ab^(−/−)^ (p16(ink4a)^(−/−)^; p15(ink4b)^(−/−)^; p19(arf)^(flox2/flox2)^)	Conditional knockout AdCre injection 3 μl (5 × 10^10^–1 × 10^11^ pfu) subdural [30, 132]	Injection: PN2 [30, 132]	CC 90 [132] SS 105 [30]	85% (17/20) [132] 72% (38/53) [30]	66% (25/38) Grade 1 [30] 32% (12/38) Grade 2 [30] 3% (1/38) Grade 3 [30] Fibroblastic and meningothelial [30] 11/17 meningothelial [132], 5/17 transitional [132], 1/17 fibroblastic [132]	Osteomas 23% (12/53) [30], liver tumor 79% (42/53) [30], subcutaneous sarcoma 34% (18/53) [30], hydrocephalus 32% (17/53) [30], ND [132]	Histology [30, 132], IHC [30], MRI [30], electron microscopy [30, 132], BLI [30], confocal microscopy [132]	1[30, 132]
Nf2^(flox2/flox2)^;ink4ab^(−/+)^ (p16(ink4a)^(−/+)^; p15(ink4b)^(−/+)^; p19(arf)^(flox2/+)^)	Conditional knockout AdCre injection 3 μl (5 × 10^10^–1 × 10^11^ pfu) subdural [30]	Injection: PN2 [30]	SS 234 [30]	50% (28/56) [30]	75% (21/28) Grade 1 [30] 14% (4/28) Grade 2 [30] 11% (3/28) Grade 3 [30] Fibroblastic and meningothelial [30]	Osteomas 32% (18/56) [30], liver tumor 59% (33/56) [30], subcutaneous sarcoma 9% (5/56) [30], hydrocephalus 46% (26/56) [30]	Histology [30], IHC [30], MRI [30], electron microscopy [30]	1 [30]
Nf2^(flox2/−)^; p16(ink4a)^(−/+)^	Conditional knockout Knock-in approach PDGS + leptomeninges cells. PDGS(Cre) [29]	PDGScre (meningeal PGDS + cells) E12.5-PN2 [29]	SS 16/24 survived 15 months [29]	50% (8/16) [29]	6/16 meningothelial, 6/16 fibroblastic (4 with concomitant tumors)—benign [29]	Osteoma 81% (13/16) [29], pituitary tumor 69% (11/16) [29], hydrocephalus 13% (2/16) [29]	Histology [29], IHC [29], electron microscopy [29] gene expression profile [29]	1 [29]
Nf2^(flox2/−)^; p16(ink4a)^(−/−)^	Conditional knockout Knock-in approach PDGS + leptomeninges cells. PDGS(Cre) [29]	PDGScre (meningeal PGDS + cells) E12.5-PN2 [29]	SS 16/22 survived 15 months [29]	81% (13/16) [29]	8/16 meningothelial, 8/16 fibroblastic (3 with concomitant tumors)—benign [29]	Osteoma 88% (14/16) [29], pituitary tumor 6% (1/16) [29], hydrocephalus 31% (5/16) [29]	Histology [29], IHC [29], electron microscopy [29], gene expression profile [29]	1 [29]
Nf2^(flox2/−)^; p53^(flox/−)^	Conditional knockout Knock-in approach PDGS + leptomeninges cells. PDGS(Cre) [29]	PDGScre (meningeal PGDS + cells) E12.5-PN2 [29]	SS 135 [29]	43% (6/14) [29]	Fibroblastic—benign [29]	MPNST 29% (4/14) [29], osteosarcoma 79% (11/14) [29], pituitary tumor 14% (2/14) [29], choroid plexus tumor 29% (4/14) [29]	Histology [29], IHC [29], electron microscopy [29], gene expression profile [29]	1 [29]
PDGF-B	Conditional knockout PGDS tv-a induced via 4 μl/2 × 10^5^ RCAS-producing DF-1 cells subdurally [31]	RCAS/tv-a system alone Injection: PN3 [31]	SS 240 [31]	26% (7/27) [31]	Benign meningiomas [31]	Gliomas 88% (23/26) [31], hydrocephalus 65% (17/26) [31]	Histology [31], IHC (PDGS) [31]	1 [31]
PDGF-B;Nf2^(flox/flox)^	Conditional knockout PGDStv-a (PDGF-B) (as described above) AdCre (Nf2^(flox/flox)^) (as described above) [31]	Injection: PN1: AdCre [31] PN3: RCAS [31]	SS 189 [31]	52% (15/29) [31]	60% (9/15) Grade 1 [31], 40% (6/15) Grade 2 [31]	Gliomas 48% (14/29) [31], hydrocephalus 7% (2/29) [31]	Histology [31], IHC (PDGS) [31]	1 [31]
PDGF-B; Nf2^(flox/flox)^;Cdkn2ab^(−/−)^	PGDStv-a (PDGF-B) AdCre (Nf2^(flox/flox)^; Cdkn2ab^(−/−)^) [31]	Injection: PN1: AdCre [31] PN3: RCAS [31]	SS 54 [31]	79% (15/19) [31]	33% (5/15) Grade 1 [31] 47% (7/15) Grade 2 [31] 20% (2/15) Grade 3 [31]	Gliomas 79% (15/19) [31]	Histology [31], IHC (PDGS) [31]	1 [31]
SmoM2 (Rosa26-lox-STOP-lox-SmoM2)	Conditional knockout PDGSCre;SmoM2 [133]	PDGScre (meningeal PGDS + cells) E12.5 [133]	SS 426 [133]	21% (9/42) [133]	All meningothelial, grade 1 [133]	–	Histology [133], IHC (Gli-1) [133]	1 [133]
SmoM2 (Rosa26-lox-STOP-lox-SmoM2)	Conditional knockout AdCre;SmoM2 [133]	Injection: PN2 [133]	SS 84 [133]	2% (1/53) [133]	1/1 Meningothelial, Grade 1 [133]	Medulloblastoma 8% (4/53) [133]	Histology [133], IHC [133]	1 [133]
YAP1-MAML2-V1 *Nestin/tv-a Cdkn2a null mice*	RCAS/tva-system. Injection of 1 × 10^5^ DF1 cells in 1 ul volume [134]	Deep Injection: PN1-3 [134]	ND(134)	42% (5/12) [134]	Meningioma-like tumors [134] 1/12 extra-axial, 2/12 intraventricular, 2/12 extra-cranial	ND [134]	Histology [134], IHC [134], RNA-seq [134], MRI [134]	1 [134]
YAP1-MAML2-V2 *Nestin/tv-a Cdkn2a null mice*	See above Conditional activation of lesion Double activation RCAS/tva-system [134]	Deep and superficial Injection: PN1-3 [134]	67–164 [134], 80–150 [134]	43% (3/7) (deep) [134] 68% (13/19) (superficial) [134]	Meningioma-like tumors [134] Deep: 1/7 extra-axial, 2/7 intraventricular, Superficial: 5/19 extra-axial, 6/19 intraventricular, 6/19 extra-cranial	ND [134]	Histology [134], IHC [134], RNA-seq [134], MRI [134]	1 [134]
NLS-2SA-YAP1 *Nestin/tv-a Cdkn2a null mice*	See above Conditional activation of YAP1 Single activation RCAS/tva-system [134]	Superficial Injection: PN1-3	80–123 [134]	97% (29/30) [134]	Meningioma-like tumors.[134] 17/29 extra-axial, 25/29 intraventricular, 25/29 extra-cranial	ND [134]	Histology [134], IHC [134], RNA-seq [134], MRI [134]	1 [134]
p16^(−/−)^;p19^(−/−)^	Injection ENU dose (carcinogen) 25 mg/kg body weight [131]	Injection: Gestation age 14 (E14) [131]	SS: 98–133 [131]	5% (2/43)^a^ [131]	Non-invasive benign [131]	^b^7/8 tumor bearing mice had multiple alveola-bronchiolar adenomas [131]	Histology(1^31)^, IHC [131], electron microscopy [131]	1 [131]
p16^(±)^;p19^(±)^	Injection ENU dose (carcinogen) 25 mg/kg body weight [131]	Injection: E14 [131]	SS: 210–273 [131]	33% (6/18)^a^ [131]	Non-invasive benign [131]	^b^7/8 tumor bearing mice had multiple alveola-bronchiolar adenomas [131]	Histology(1^31)^, IHC [131], electron microscopy [131]	1 [131]
p16^(+/+)^;p19^(+/+)^	Injection ENU dose (carcinogen) 25 mg/kg body weight [131]	Injection: E14 [131]	ND [131]	0% (0/24) [131]	–	–	–	1 [131]

SS: Survival study—number of days 50% dead (control animals) or human endpoint met. The detailed data extraction sheet is available in Additional file 11

NLS: N-terminal nuclear localization sequence, SCH: Schwann cell hyperplasia, MPNST: Malignant peripheral nerve sheath tumor, pfu: Plaque-forming units, ENU: N-ethyl-N-nitrosourea

^a^includes both meningiomas and meningiomatosis—overestimate of tumor take rate

^b^from same study, but not described further, PGDS: Prostaglandin D2 synthase

The TTR for *Nf2*^*(flox2/flox2)*^ was 29% (95% CI 19–41) via AdCre injection orthotopically at PN1-3 to only target the *Nf2* gene on both alleles over a duration of 117–450 days. The tumors were benign histologically, with transitional, meningothelial, and fibroblastic subtypes. However, a variety of other pathologies arose such as osteomas at the burr hole (51%), liver tumors (17%), and hydrocephalus (34%). *Nf2*^*Flox*^ transgenic mice have been crossed with various other genes to assess the interactions. Waldt et al*.* [130] tested loss of the potential meningioma tumor suppressor receptor-like density-enhanced phosphatase-1 (*DEP-1*) [99], encoded by *PTPRJ*. They showed no TTR in *PTPRJ*^*−/−*^ transgenic mice alone but raised TTRs ranging from 6% (0–27%) in their *Nf2*^*Flox2/Flox2*^ to 25% (13–40%) in *Nf2*^*(flox2/flox2)*^;*Ptprj*^*(−/−)*^, all over the same time period of one year for typical meningiomas with whorls and psammoma bodies, thus suggesting an interaction between the two genes in meningioma development.

It is well known that loss of the tumor suppressor *p53* can cause tumor development through various pathways[158], as tested in congruency with *Nf2* by Kalamarides et al.’s first GEM paper from 2002 [27]. They showed a 30% TTR in the *Nf2* lesion alone and only 13% (6–28%) in *Nf2*^*flox/flox*^*;p53*^±^ (heterozygous *p53*), however with a 91% rate of sarcomas/osteosarcomas over the course of a mere 165 days (median survival). A conditional homozygous lesion of *p53*^*flox/−*^ with *Nf2*^*flox/−*^ was also tested by the same group using the cell-specific prostaglandin D2 synthase (*PDGS;*Cre), which affects the fetus during intrauterine development. The authors found a higher TTR for *Nf2*^*(flox2/flox2)*^*;p53*^*(flox2/−)*^ of 45% (23–68%), but it was still lower than the TTR of 50% (29–71%) for the corresponding *Nf2*^*flox2/−*^ alone. There was again a high number of malignant tumors, 79% osteosarcomas, and an even shorter survival of 135 days. The authors identify PGDS + arachnoid cells as a cell of origin for meningiomas [29].

Also of great interest are the tumor suppressor genes *CDKN2A/B* (located at 9p21 in humans). In meningiomas, alterations of *CDKN2A/B* are more common in higher grade tumors and are associated with high clinical recurrence [159, 160]. *CDKN2A* encodes the p16^INK4a^ and p14^arf^ (p19^arf^ at chromosome 4 in mice [161]). p16^INK4a^ regulates G_1_/S-phase via inhibition of cyclin-dependent kinases Cdk4 and Cdk6 [162], and p14^arf^ regulates activity of *p53* [163]. Adjacent to *CDKN2A* lies *CDKN2B*, which encodes the p15^INK4b^ that also inhibits Cdk4 and 6 [164]. A TTR of 37% (95% CI 23–55%) was obtained by exploring only *CDKN2A* alteration (INK4a) using the AdCre method [28] and a TTR of 82% (95% CI 60–95%) from exploring the *PDGS;*Cre method [29]; these were primarily in benign tumors with few tumors showing atypical features and with other pathologies such as osteomas (78% and 88%, respectively) and hydrocephalus (56% and 31%, respectively). Further exploration of full *CDKN2A/B* hetero- and homozygous deletion led to creation of a *Nf2*^*(flox2/flox2)*^*;ink4ab*^(−/(−/+))^ AdCre model. This showed a higher TTR of 76% (95% CI 61–86%) in homozygous [30, 132] compared to heterozygous 50% (36–64%) [30], with a higher take in sarcomas (34% vs 9%) and liver tumors (79% vs 59%). The homozygous deletion found 66% grade 1, 32% grade 2, and 3% grade 3, whereas the heterozygous deletion found 75% grade 1, 14% grade 2, and 11% grade 3 [30]. However, all homozygous tumors would be classified as malignant in accordance with the newly implemented WHO classification 2021 [165] due to *CDKN2A/B* homozygous deletion as an independent criterion of WHO grade 3 meningiomas.

Lastly, Morrison et al*.* [131] induced tumors using transgenic mice models of *p16* and *p19* wildtype, hetero- and homozygous and the carcinogenic compound N-ethyl-N-nitrosourea (ENU) as intraperitoneal injection at E14. They showed a TTR of 6% (95% CI 2–19%) and survival of 98–133 days for homozygous vs TTR of 31% (95% CI 7–75%) and survival of 210–273 days for heterozygous. Concomitant alveola-bronchiolar adenomas were present in almost all tumor-bearing mice. Wild type showed no meningioma tumors.

It has long been suggested that platelet-derived growth factor (PDGF) exhibits tumorigenic properties in meningiomas [166, 167]. Using the RCAS/TVA system, a PDGF-B model was created that showed a TTR of 27% (95% CI 12–48), all benign. However, the model also yielded 88% gliomas and 65% with hydrocephalus with a survival median of 240 days [31]. Furthermore, PDGF in combination with AdCre;*Nf2* gave a higher TTR of 52% (33–71%) with 66% being grade 1, 40% grade 2, and 20% grade 3; however, there was still a high number of gliomas (48%) and a shorter median survival of 189 days. Lastly, they combined *PDGF-B;Nf2;CDKN2AB* lesions and found an even higher tumor take rate of 79% (54–94%) with 33% grade 1, 47% grade 2, and 20% grade 3 (but the same applies here as with the above *CDKN2A/B*^*−/−*^ in relation to the malignancy grade). Median survival was greatly decreased to 54 days, and glioma incident remained high (79%).

*SMO* is a member of the Hedgehog (Hh) signaling pathway and is present in a small percentage of meningiomas (5%), specifically the meningothelial subtype [168]. It is a suggested oncogenic driver and is frequently associated with *PI3K/AKT/mTOR* pathway in driving tumor formation in meningiomas [168]. Boetto et al*.* explored this utilizing both PDGSCre; *SMO* and AdCre; *SMO* GEMs [133]. They found a TTR of 21% (95% CI 10–37%) in PDGSCre vs 2% (0–10%) in AdCre; all were meningothelial subtype with median survival of 426 days vs 84 days. The AdCre model besides having a shorter survival also produced medulloblastomas in 8%. The results suggest that *SMO* activation is restricted to a prenatal window E12.5 as is the case with PDGSCre.

Finally, Szulzewsky et al*.* [134] recently explored Yes-associated protein 1 (*YAP1*), which is involved in functional inactivation of *Nf2* in heterozygous cases. *YAP1* is a transcriptional coactivator of cell growth that is regulated by the Hippo signaling pathway and is especially associated with pediatric *Nf2* wild-type meningiomas [169]. *YAP1-MAML2* exerts oncogenic *YAP* activity that is resistant to inhibitory Hippo pathway signaling and relies on the interaction with TEAD transcription factors. Utilizing RCAS/TVA system and a Nestin/TVA *CDKN2AB* null mouse strain, it was found that the TTR for *YAP1-MALM2*(v1 and v2)’s was 42% (95% CI 15–72%) to 60% (95% CI 36–80%) of cases over the course of 67–164 days. A nuclear localization sequence (*NLS)-2SA-YAP1* lesion—which constitutively activated *YAP1* to inactivate *Nf2*—was explored to determine whether it would suffice to produce meningioma-like tumors. The authors showed a very high TTR in 97% (95% CI 83–100%) of the animals and verified their results with RNA sequencing. The study did not describe other pathologies present in the animals.

#### Strengths and weaknesses/limitations of GEM in meningiomas

In contrast to xenotransplantation in immunodeficient animals, GEM develop de novo tumors in immunocompetent animals [175]. GEM can thus be used to investigate candidate cancer genes (e.g. driver mutations), determine cancer cells-of-origin by altering specific targeted cells, and study the contribution of tumor microenvironment due to the intact immune system. GEM could thus be helpful in validating drug targets [175].

Although GEM have been of great value in cancer research, they have several disadvantages. The major weakness of Cre-*loxP* is that it does not allow for sequential and time-specific stepwise activation or inactivation of specific genes in vivo. This means that although Cre-*loxP* can assess single gene lesions, the model does not accurately reflect all aspects of sporadic multistep carcinogenesis [176]. The major weakness of RCAS/TVA is the limited insert capacity of the virus (2.8 kb), which limits the genes that can be evaluated [177]. Furthermore, producing a germline GEM is labor-intensive, time-consuming, and expensive [175]. Despite the clear advantages of a de novo tumor in an immunocompetent environment, the tumor remains a mouse tumor and not a human tumor—and these may act/react differently, e.g. CDKN2A/B is present at chromosome 9 in humans but chromosome 4 in mice [161, 162].

### Uncategorized models

These studies were deemed too heterogenous and too few to be included in the meta-analysis and subgroup analyses. A narrative approach was chosen to describe the studies of most interest, in addition to a table (Table 7) (for further details Additional file 12).

**Table 7:** Characteristics of uncategorized models

Uncategorized models						
Method overview/paper		Specific aim	MoEMM	ToA/age (w)/NoA	DoI	Results
Syngeneic models	Peyre et al. 2012 [30]	Xenografting cells (MGS1-3) derived from genetic engineered model (Nf2^flox/flox^;Ink4ab^−/−)^	Orthotopic injection 1.5 × 10^4^–7 × 10^7^ cells/μl in 7–10 μl	FVB wild type mice/6/30	MGS1 1.3 m MGS2 0.6 m MGS3 1.3 m	TTR: MGS1 = 5/10 grade 1, 4/10 grade 2, 1/10 grade 3; MGS2 = 10/10 grade 3; MGS3 = 2/10 grade 1, 4/10 grade 2, 4/10 grade 3^a^
	Peyre et al. 2013 [132]	Utilizing implantation of genetic-engineered tumor cells MGS2 (30) in ascertaining handheld confocal microscopy to identify focal brain invasion	Orthotopic injection 1.5 × 10^4^–7 × 10^7^ cells/μl in 8 μl	FVB wild type mice/-/20	14d	TTR: 17/20 tumors in total 11 meningothelial, 5 transitional, 1 fibroblastic Confocal microscopy identifies brain invasion along Virchow-Robin spaces and identification of intratumoral vessels and nerves
	Yeung et al. 2021 [135]	Test of anti-programmed death ligand (PD-L1) and 4-1BB(CD137) anti-colony-stimulating factor 1 (CSF1)/colony-stimulating factor receptor (CSFR) in a syngeneic model using cells MGS1[30]	Orthotopic injection (2.5 × 10^5^ cells in 25 μl) and heterotopic injection (1 × 10^6^ cells in 100 μl)	FVB wild type mice/6–8/15	30–54 days depending on experiment In survival 50% dead after 44 days in control group	No upregulation of PD-L1 in vivo due to paucity of T-cell infiltration—hence, T-cell targeted therapy did not decrease tumor size CSF1 and CSF1R (mediators in monocyte recruitment, M2-differentiation) Anti-CSF1 significantly reduce tumor size
	Yamate et al. 1994 [142]	To investigate a transplantable tumor (MM-KMY) derived from a malignant meningioma (spontaneous) in an F344 Rat	Heterotopic implantation	F344 rats/3-30w/73	6-8w (various passages in paper)	Eight weeks until nodule avg diameter 5.7 cm, which formed large cysts and necrotic tissue. Frequent metastasis in lungs. Xenograft tumors similar to parent tumor Vimentin positive xenograft and parent tumor. 100% TTR They also show positive monocytes/macrophage infiltration
	Tsujino et al. 1997 [143]	To establish KMY-J from MM-KMY tumors and develop clones	Heterotopic injection of 1 × 10^6^ cells of clone KMY-1–3	F344 rats/6-14w/-	9-11w	Established clones of KMY-MM with varying cell morphology and chromosomes. Palpable tumors after 5–7 weeks TTR not described
Xenografting human-derived stem-like cells	Hueng et al. 2011 [140]	To investigate patient-derived Meningioma Stem-Like Cells (MgSCs) and adherent cells (MgACs)	MgSCs (meningioma sphere cells) or MgACs (meningioma adherent cells) 1 × 10^4^ cells injected in 5 μl orthotopically	NOD/SCID mice/6-8w/30	60d	MgACs were not tumorigenic. MgSCs were, but no mentioning of TTR. Similar immunohistochemical profile to parent tumor
	Rath et al. 2011 [141]	To isolate and characterize a population of Stem-like progenitor cells from an atypical meningioma. Identifying tumor-initiating cells	Heterotopic injection of 10^3^, 10^4^, 10^5^, 5 × 10^6^ meningioma-initiating cells (MICs)	Foxn1(nu) mice/6-7w/12	Up to 12w	EMA positive, Vimentin positive, GFAP negative. MICs self-renew, differentiate, and can recapitulate the histological characteristics of the parental tumor Model usable for studying tumorigenesis
Xenografting non-neoplastic cells	Baia et al. 2012 [139]	To investigate Yes-Associated Protein 1 as an oncogene in meningiomas	Orthotopic implantation of non-neoplastic arachnoidal cells, AC1, vector and transfected with YAP1 and luciferase (10^5^ cells)	Athymic nude mice/6w/12	Up to 90 days. Median survival in YAP1 mice 22 days	0/6 xenografts in control/vector vs. 6/6 YAP1 transfected Large well-circumscribed tumors
Corneal Angiogenesis Assay	Toktas et al. 2010 [136]	To investigate relationship between angiogenetic potential of intracranial meningiomas using rat corneal angiogenesis assay (CAA)	Implantation of various tumor grades in corneal micro pockets	Sprague–Dawley rats/-/60	20d	Directly correlated to WHO grade. Higher grade = more vessels. Glioblastomas = most vessels fastest No differences in tumors exhibiting peritumoral edema and no edema—However tendency Furthermore, recurrent tumors exhibit more vessels than non-recurring tumors
	Kilic et al. 2013 [137]	To investigate inhibitory effect of gamma knife irradiation on angiogenesis of meningiomas	Implantation of various tumor grades in corneal micro pockets	Sprague–Dawley rats/-/72 3 groups	20d	No differences in number of vessels for grade 3, only high dose 22 Gy for grade 2 and only 18 + 22 Gy for grade 1 were significant
Altered cells using virus	Brooks et al. 1988 [138]	To investigate tumor induction rate of Simian Virus 40 (SV40)-transformed human meningioma cell injection	Transformed cells (normal fetal brain and meningioma) with SV40 (Simian Virus 40—disputed oncogenic virus). Heterotopic injection 2 × 10^6^ cells	Athymic nude (nu/Cox)/6/45 3 groups	Up to 74w	TTR 0/15 of normal fetal brain injection/SV40 Meningioma/SV40 6/15 (4 lymphomas and 2 fibrosarcomas) TTR and SV40 virus alone TTR 6/15 all fibrosarcomas

MoEMM: Method of Establishing Meningioma Model. ToA: Type of animal, TTR: Tumor take rate, age(w): Age in weeks. NoA: Number of animals. DoI: Duration of Incubation, m: months, w: weeks, d: days

^a^Not based on WHO 2021 classification. Full detailed Data Extraction Sheet is available in Additional file 11

Syngeneic models were first tried in 1994 by Yamate et al. [142], who excised a highly malignant cerebellar tumor from an F344 rat; this was consecutively passaged and transplanted with great success (100% TTR) into new animals. Furthermore, the models showed similar histological traits to the parent tumor. Peyre and Kalamarides in collaboration with Yeung et al*.* [30, 132, 135] have performed 100% TTR fast-growing syngeneic malignant models (both orthotopic and heterotopic) using cell lines derived from Peyre et al*.* GEM (MGS1-3) [30]. This model type provides a stable method of assessing WHO grade 3 meningiomas and their interaction with the immune system as the animals are immunocompetent. Thus, it is a genetically modifiable alternative to the spontaneous animal-derived tumor studies from the 1990s [142, 143].

Developing meningiomas in an animal model has also been successful from patient-derived tumor stem-like cells [140, 141]. These studies show tumors reflecting the histological features and immunohistochemistry of the parent tumors. This model type could be used for assessing tumorigenesis of various progenitor cells. Baia et al. [139] transfected non-neoplastic arachnoidal cells with Yes-Associated Protein-1 and found 100% tumor in comparison to no tumor in controls. Tumorigenic studies on specific proteins or other factors could be performed in a similar fashion.

### Designing a model: type and validation of growth and verification of tumor

The preferred model depends on multiple factors such as the type of research and availability of resources (specialized equipment, scans, laboratory analyses). This section gives an overview via Table 8 of the advantages and disadvantages of the different model types, and we present a separate section on validation of growth and verification of tumor.

**Table 8:** Advantages and disadvantages of various models for meningiomas

Model	Description	Advantages	Disadvantages
Heterotopic xenograft	Implantation/xenografting of either cells or whole tumor pieces in a site other than intracranial/intraspinal		
• Flank/subcutaneous	Implantation/xenografting of either cells or whole tumor pieces subcutaneously either via scalpel or injection. Matrigel can be utilized for a fixed position	Easy to implant, easy to measure and follow growth, inexpensive	Caliper measurements are estimates due to artefacts from skin and fatty tissue
• Subrenal	Surgically implantation of cells in the subrenal capsule. For technique see [127]	More accurate measurements compared to subcutaneous technique	Implantation and measurements require surgery and strain of the animals
Orthotopic xenograft	Implantation/xenografting of either cells or whole tumor pieces at dura attached areas intracranially and intraspinally		
• Superficial	Implantation superficially most often through a burr hole in the frontal region	Easy to locate, low risk of bleeding perioperatively	Risk of cell reflux, if not careful
• Skull base	Implantation at the skull base most often through a burr hole in the frontal region	Fixed position, low risk of cell reflux	Depending on head angle of animals during implantation different placements on skull base Higher risk of bleeding
• Post-glenoid foramen	Natural cavity in rodents Located on the rostral area of the opening of the external acoustic meatus Different angles for different sites from cerebrum, cerebellum to brain stem and basal cistern [182]	Can be performed via subcutaneous injection, short procedure time, accessible to researchers without surgical skills [182]	Requires a sharp needle, increased risk of bleeding, handheld injection
Xenografted material			
• The use of patient-derived primary material	Patient-derived samples obtained through surgery without immortalization. Benign tumors in particular are prone to senescence	Recapitulates each individual tumor more accurately to be used in personal targeted therapy	Varying TTRs between and within each patient derived tumor Mix of host DNA and human xenograft
• Using cell suspension	Injection of a suspension of cells	Easy to control number of cells used. Produces similar conditions for all animals in drug trials	Has to develop from cell suspension to tumor -Morphological changes can occur Only most viable cells survive culture
• Using whole tumor pieces	Implantation of whole tumor pieces from surgical specimens	Xenograft is morphologically representable to parent tumor	Difficult to make sure representable pieces of tumor is implanted and that it is consistent throughout animals
• The use of established/commercially available material	Immortalized patient-derived cells Often purchasable through biobanks or cell companies such as ATCC (American Type Culture Collection)	Near 100% tumor-take rate—need for a small number of animals Homogeneous tumors across all studies Can perform genomic alterations to cells to study differences [79]	Growth patterns do not represent primary meningiomas due to immortalization and homogenous cell population
• Syngeneic cell implantation	Use of cells deriving from the same species—in this setting murine meningioma to murine host	High tumor-take rate in immunocompetent animals	Assessment of mice derived tumors—Possible problems in translating to humans
Genetically Engineered Models	Knock-out or knock-in of genes through genetic manipulation (i.e. Cre-recombinase or RCAS/TVA)	Valuable tools for preclinical drug testing and for studying the underlying oncogenic drivers and molecular pathways in tumors	Expensive, labor intensive, other pathologies connected to genetic lesion
Corneal implantation	Implantation of tumor material in corneal pocket	Easy assessment of vessel growth through fundoscopy	Heterotopic model, loss of microenvironment

#### Validation of growth and verification of tumor

During any experiment, it is important to follow growth at various time-points, e.g. before, during, and after treatment to assess the efficacy of the intervention. Due to the associated skin and fatty tissue, the size of a tumor xenografted subcutaneously is more difficult to estimate via caliper. A subrenal capsule approach can give more precise measurements but requires surgery for each measurement at the cost of animal strain [106, 127, 129]. A single study reports peritoneal injection [58], but this approach has the same disadvantages as orthotopic models without any benefits. It is difficult to assess tumor growth in orthotopic models without sacrificing the animals and without specialized non-invasive methods such as bioluminescence, MRI, and PET (Tables 4, 5 and 6). Bioluminescence is widely used for real-time imaging in vivo. Once cells have been transduced with luciferase (Fluc) prior to implantation, it is relatively easy and inexpensive [178]. MRI scans are also widely used but usually require special high-field MRI scanners for optimal resolution on a small scale (3.0T [67], 4.7T [28, 30, 53, 61, 62], 9.4T [25, 83]), but can be achieved to a certain degree with a clinical 1.5Tesla MRI [81].

Meningioma verification in the clinic depends primarily on HE histology and perhaps the immunohistochemical markers vimentin, epithelial membrane antigen (EMA), somatostatin receptor 2 (SSTR2), and Ki67/MIB-1. However, newer guidelines include genome sequencing on special cases [165]. Particularly when implanting primary patient-derived cells or tissue, researchers should perform a panel of HE and immunohistochemical markers and compare to the parent tumor at the very least, however it is recommended to compare at a genomic [179] or epigenomic level. During inoculation of cancer cells in mice, the stroma is replaced by mouse cells, resulting in a mix of DNA [180]. It was shown in pancreas cancer that xenografts in immunocompromised animals were contaminated with 47% (17–73%) mouse DNA [181]. To our knowledge, no such experiments have been conducted in meningioma research, but it is important to take this into account when discussing results of a treatment. In regard to verifying and classifying GEM-derived tumors, Kalamarides and colleagues have suggested a specific classification based on histological analysis, which reflects tumor composition more accurately than the human classification [35], which should be used in case of GEM.

### Critical appraisal of methodological quality and quality of reporting (CRIME-Q)

**Fig. 3:**
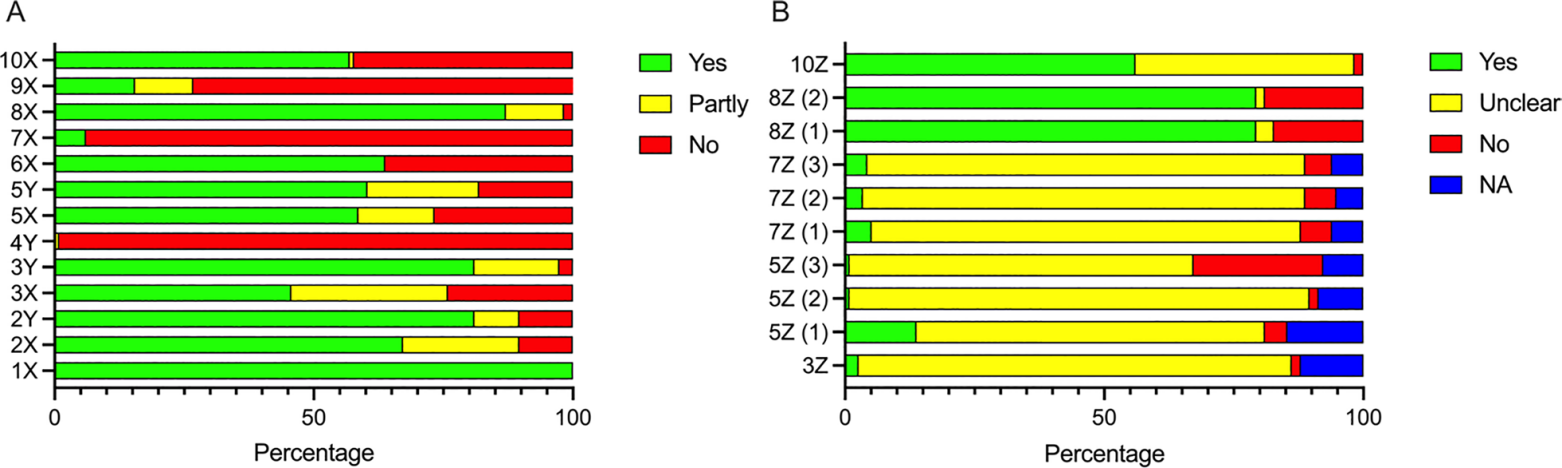
CRIME-Q—Quality of Reporting, Methodological Quality and SYRCLE’s Risk of Bias. Graphical display of all categories 1–10 including all included studies **A** Quality of Reporting items (X) and Methodological Quality (Y) Green = Yes, Yellow = Partly, Red = No. **B** Results of Items from SYRCLE’s Risk of Bias tool. Green = Yes, Yellow = Unclear, Red = No, Blue = Not applicable. **1X** Peer-review, **2X** Bench-top/laboratory work related to establishing model, **2Y** Methodological quality of 2X, **3X** Animals, **3Y** Methodological quality of 3X, **3Z:** Selection bias (baseline characteristics), **4Y** Sample size, **5X** in vivo design and performance, **5Y:** Methodological quality of 5X, **5Z**(1)**:** Selection bias (Sequence generation), 5Z(2): Performance bias (Random housing), **5Z**(3): Detection bias (Random outcome assessment), **6X:** Compliance with animal welfare regulations, **7X** Blinding, **7Z(1)** Performance bias (Blinding), **7Z(2)** Allocation bias (allocation concealment), **7Z(3)** Detection bias (blinding), **8X** Congruency between methods and results, **8Z(1)** Attrition bias (incomplete outcome data), **8Z(2)** Reporting bias (Selective outcome reporting), **9X** Presentation of limitations, **10X** Statement of potential conflict of interest, **10Z** Other bias (Publication bias). See Table 1 for further descriptions

All studies included in this meta-analysis were peer-reviewed, assuring a certain quality. We considered 67% of all studies to sufficiently describe the bench-top part of the experiments, but only 46% described the animals properly. Almost all studies did not calculate sample size, and no studies provided a full description of the sample size calculation. A full description of the in vivo experiment elements was present in 59% of the studies, and 27% had such deficient descriptions that the experiment could not be replicated. Around two-thirds of the studies described compliance with animal welfare, and almost no papers (6%) were blinded in any way. It is not standard practice in animal research to discuss model limitations, which was reflected in three-quarters of the studies not mentioning this. In general, we found a higher percentage of studies with good methodological approach (2Y, 3Y, 5Y) than with good quality of reporting (2X, 3X, 5X), see Fig. 3A**.**

SYRCLE’s risk of bias items were included in the CRIME-Q tool. Items 3Z, 5Z [1–3], and 7 [1–3] were mostly unclear in 67–89% of the included studies. A few studies were non-interventional, so some items were not applicable. We found that 79% of studies had a low risk of excluding outcome data (Attrition bias, 8Z [1]). Although no study protocols were apparently available, it was clear in 79% of studies that the published reports included all expected outcomes (i.e., comparing the Methods and Result sections) and reported key elements, which would have been expected from such studies (i.e. tumor-take rate) (Selection bias 8Z [2]). No risk of influence from third party was found in 56% of studies (Publication bias 10Z) Fig. 3B.

## Discussion

This meta-analysis showed that the tumor take rate (TTR) for established cell line models (ECLMs) was near 100% for both orthotopic models [94% (95% CI 92–96)] and heterotopic models [95% (95% CI 93–96)]. These results proved consistent regardless of subgroup category, time frame (duration of incubation), number of cells, injection volume, cell concentration, and cell line (Additional file 13). TTR was more variable for primary patient-derived tumor models (PTMs), with 53% (95% CI 33–72) for orthotopic models and 82% (95% CI 73–89) for heterotopic models. Subgroup analyses for PTM showed varying TTRs, e.g. high (98%; 89–100%) for orthotopic duration of incubation between 14 and 30 days, but low [8% (95% CI 3–21%)] for 200–499 days of incubation. We could not identify any pattern in take rate according to the number of cells, e.g. 100–1000 cells had high TTR [86% (95% CI 42–100%)], 10.000–100.000 had very low TTR [15% (95% CI 5–37%)], and 500.000–1.000.000 cells had higher again [91% (95% CI 77–97%)].

Two aspects should be considered before applying these results in models. First, we found few published papers on PTM section and secondly, only a few studies have published negative tumor takes (0%) in some samples, namely Ragel et al. [67], Malham et al. [122], Hu et al. [121], and most importantly, Zhang et al. [25] who published several individual tumors unable to obtain growth in vivo. To our knowledge there has been no investigation into why some tumors simply fail to take in animals, and no papers have focused on extensive genomic/epigenomic alterations in meningioma xenografts. One explanation could be lack of certain driver mutations in tumors that help develop tumors in the animals. Our own experiences regarding inter-tumor heterogeneity of take-rate and knowledge within the field lead us to believe, there is a high risk of publication bias in the current analysis as not all eligible studies may have been published. If unpublished studies are more likely to show negative results, this may have skewed our results. There is a larger focus on publishing negative results today [183], but it remains an issue overall as suggested in this paper. Although submission of animal study protocols is recommended and feasible through open access journals without peer-review, it is still not common practice.

Most (70%) of the studies presented here were based on immortalized cell lines, which by far mimic proper tumor qualities. The ability of these cell lines to provide a steady, fast, and homogeneous growth makes them especially suitable to quickly test experimental treatment strategies as an ‘add on’ in often times very well performed in vitro studies with (often) very few animals i.e. [57, 64, 74, 75, 78, 80, 88, 93, 102, 103]. This may explain the low quality of reporting, which is problematic for the research community. These studies sometimes use a heterotopic model and a very early treatment start day (1–2 weeks) [52], thus not mimicking normal tumor pathology or treatment in any way. Despite this, ECLMs have a role to play in exploring, for example, scan modalities, cognitive function studies, and tumor load studies, where the specific tumor characteristics are less important.

The TTR of 34% across all GEM studies indicate a problem of finding a strong oncogenic driver that can be used to model meningioma with its typical benign nature and slow growth, which presents challenges in preclinical research [134]. The only study that reported solid growth in almost 100% of the cases was based on constitutively activated *YAP1* [134], but meningiomas initiated by *YAP1* fusion are a rare subset of childhood and young adulthood meningiomas [184]. Given sufficient time, sample size, and appropriate design to avoid an underpowered study [185], the lower take rates of GEM would not be problematic in pre-clinical pharmaceutical tests. Otherwise, the syngeneic model could be used for faster growth at higher take. Translatability from GEM (mouse tumor) to human in preclinical treatment studies in meningioma still remains to be seen.

The average rate of successful translation from animal models to clinical cancer trials is generally around 8% [23], and meningiomas are no exception. As there are currently no known pharmaceuticals with sufficient clinical benefits [186–188], in vitro and in vivo research in this field is paramount. Possible issues in relation to this loss in translation are meningioma heterogeneity as a group in terms of genetics/epigenetics and histology, microenvironmental challenges and design/use of model. Gene expression, epigenetic profiling, molecular markers, and DNA-technologies have over the past decade especially helped uncover the heterogeneity. And in line with this, the continuing search for the perfect model, which has not been discovered and might not exist. However, we can strive towards model improvement and development. Furthermore, we ought to use the ‘right’ type of model, which mimics the condition researched/reflects research question as close as possible to decrease the gap in translation.

Poor study reporting leads to irreproducible and uncertain findings [189, 190]. In this study, we present a new tool (CRIME-Q) to assess all kinds of animal studies quickly and thoroughly on multiple parameters. CRIME-Q identified issues in reporting, which influences methodological quality and risk of bias greatly, further emphasizing the need for research to report their methods and findings more thoroughly to ensure transparency, replicability and ultimately usability of models. CRIME-Q unifies multiple aspects of quality of reporting, methodological quality, and risk of bias from bench-top to in vivo design and performance and related items in between including risk of bias and presents a clearer overall assessment of included studies in systematic reviews on animal research. Used alongside the ARRIVE 2.0 guidelines for animal studies [41], CRIME-Q can help improve study transparency and replicability. We found it necessary to develop our own broader method, which also includes bench-top assessment, since we found no suitable options available [191]. Our tool is not yet externally validated, but we present all the results here in a transparent manner for further inspection.

It is important to note that this review intended to assess animal models only. Many of the included papers used animal studies to underpin the in vitro findings or as proof of concept, which will have influenced the quality of reporting, methodological quality, and risk of bias. The focus of these papers simply is not animal studies and thus our judgements should not be regarded as overall judgements of quality of the studies, but rather as judgements of the reporting and methodological quality in relation to our objectives. Individual study scores can be assessed in the Additional file 13, where a short description and grade is given for each category. Furthermore, many of the studies were published over 15 years ago, when there was less focus on full reporting.

### Study strengths and limitations

A strength of this study is the systematic approach in accordance with the PRISMA guidelines, where we made individual assessments of study eligibility and data extraction. The structured critical appraisal of methodological quality and quality of reporting of all included studies allowed us to judge the overall reliability of the studied body of evidence. A further strength is the meta-analysis on TTRs for the various model types, allowing us to assess the effectiveness of different models.

The study also has several limitations. First, there is a high risk of publication bias, as noted above. If studies with high success rates are more likely to be published, meta-analyses might overestimate the overall success rate. Likewise, if studies using specific methods are more likely to be published, this might limit the ability to compare success rates for different methods. Secondly, while we critically appraised all included studies, we used a non-validated tool. We believe we have included the most important methodological aspects, but some could have been overlooked, and we welcome feedback and criticism of the tool from the scientific community. Lastly, the methodological quality and the quality of reporting of included studies varied. Our results (including from the meta-analyses and subgroup analyses) should thus be interpreted with caution.

### Future perspectives for meningioma models and knowledge gaps

We describe here some selected points of interest in our search to identify and analyze knowledge gaps in the use of meningioma animal models for optimal preclinical tests.

First, previous successful in vivo pharmaceutical trials in xenograft models in meningiomas have failed to translate to human conditions. This is probably due to microenvironmental challenges from the lack of a proper immune response in immune-incompetent animals. Use of humanized animals [192] could help this, i.e., by engrafting CD34 + human hematopoietic stem and precursor cells to encourage development of a normal immune system [193]. The use of humanized animals with co-engrafted stromal and immune components is not yet a perfect science [192], but the technology has great potential for both tumor growth and drug response studies. Its uses remain to be seen in meningiomas.

Second, 68 Ga-DOTA(TOC/NOC/TATE) PET-CT provides high-contrast images of meningiomas due to the abundance of somatostatin receptor 2 in meningiomas but not in brain (except the pituitary gland) and bone. PET-CT has shown to be more sensitive than MRI to detect meningiomas [194, 195] and is widely used in the clinic [196]. Only two preclinical studies have assessed PET in heterotopic models, both of which showed easy distinction between healthy tissue and tumor [95, 96]. These findings need to be verified in an orthotopic model that could be used in both GEM and xenograft models.

Third, previous comparisons between primary patient-derived tumor and corresponding xenograft in meningioma research have primarily used histology and immunohistochemistry. Only Zhang et al. [25] assessed two tumors (WHO grade II and III) and corresponding xenograft gene expression profiles using RNA sequence to assess DNA copy number variations and genes. The xenograft tumors retained all of the copy number variations seen in the original tumors. When comparing original tumor to xenograft and normal brain tissue, the authors found high correlation between original tumor and xenograft genes. Moreover, a large study involving several hundred xenograft cancer models of many types showed no enrichment of cancer-related genes in xenografts and concluded a lack of systematic copy number evolution driven by the PDX mouse host [197]. Despite these findings, we lack epigenetic knowledge of meningioma xenograft using DNA-methylation (especially in the benign tumors, which have not yet been tested). We should explore this approach further to identify the model type that best mimics human conditions.

Finally, we have limited knowledge of GEMs and oncogenic drivers, as well as the impact of different genes e.g., *TRAF7, AKT1*. The observed TTR of 34% for GEMs indicates challenges in finding a strong oncogenic driver that can be used to model this disease with the Cre-recombinase and RCAS/TVA systems. Conditional or ‘time- and site-specific’ DNA alteration is important in various diseases as some gene alterations are not compatible with intrauterine development [151], e.g. homozygous *Nf2*-deleted mouse embryos fail in development [198]. The CRISPR/Cas9 technology can target any genomic point through single-guide RNAs [199] and can be used to introduce defined mutations or *loxP*-sites [175]. It is especially useful in non-germline models based on direct gene editing in vivo. This could be used to quickly identify oncogenic genes without the need for extensive breeding to obtain the proper transgenic model [200].

## Conclusion

This systematic review shows high consistent tumor take rates in established cells lines and varying tumor take rates in primary-patient derived material and genetically engineered models. However, we identified various issues across the studies regarding the quality of reporting, the methodological approach, and a high risk of publication bias. Each tumor model type has specific roles. ECLMs are useful for modality testing and other tumor burden studies, while PTMs (orthotopic) mimic tumor heterogeneity and have low cost and technical skill requirements, making them useful in initial pharmaceutical testing if appropriate examinations are performed (e.g. DNA sequencing, DNA methylation). Finally, GEMs are useful in assessing and validating driver mutations and determining cells-of-origin, making them relevant in pre-clinical testing due to an intact immune system; they may also be beneficial in validating drug targets.

### Supplementary Information

**Additional file 1: **The PRISMA checklist.

**Additional file 2: **The PRISMA abstract checklist.

**Additional file 3: **Uploaded PROSPERO protocol.

**Additional file 4: **Protocol breaches.

**Additional file 5: **Full original search strategy.

**Additional file 6: **Data extraction fields.

**Additional file 7: **CRIME-Q: Full spread sheet.

**Additional file 8: **CRIME-Q: Predetermined list of information.

**Additional file 9: **ECLM full data extraction sheet.

**Additional file 10: **PTM full data extraction sheet.

**Additional file 11: **GEM full data extraction sheet.

**Additional file 12: **Uncategorized full data extraction sheet.

**Additional file 13: **Meta- and subgroup analyses.

## Data Availability

All data generated or analyzed during this study are included in this published record [and its Additional files].
