# Supplementary material for: Meningioma animal models: a systematic review and meta-analysis
Source: J Transl Med. 2023 Oct 28;21:764. doi: 10.1186/s12967-023-04620-7 (PMC10612271; doi:10.1186/s12967-023-04620-7)
Supplement: Supplementary file 3 — Additional file 3: Uploaded PROSPERO protocol. [file 12967_2023_4620_MOESM3_ESM.docx]

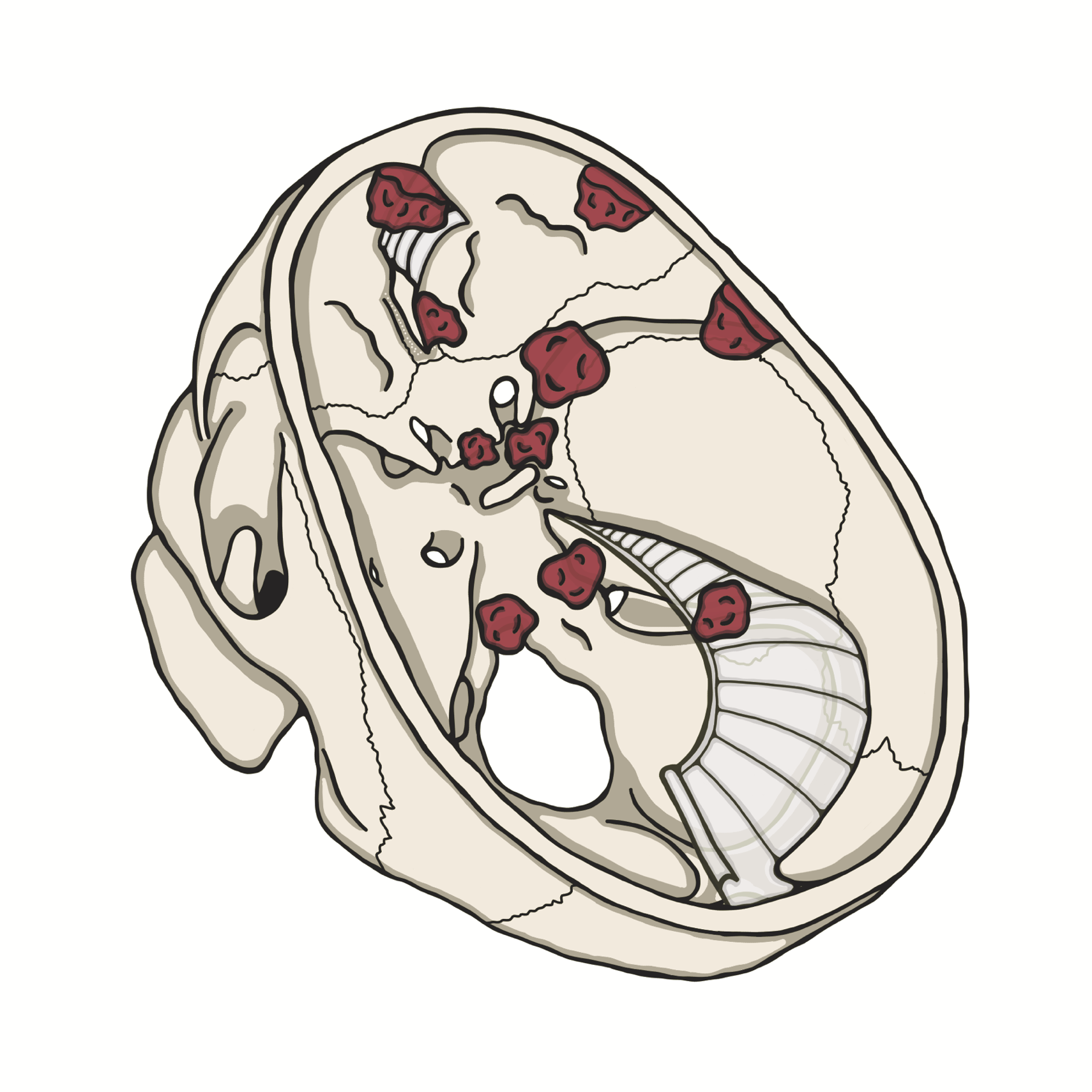
Past, Present and Future of Meningioma Animal Models – Systematic Review Protocol

Mikkel Schou Andersen, resident, PhD-student, MD^1,2^; Mikkel Seremet Kofoed, resident, PhD-student, MD^1,2^; Associate Prof. Christian Bonde Pedersen, consultant, MD, PhD^1,2^; Associate Prof. Bo Halle, consultant, MD, PhD^1,2^; Prof. Tiit Mathiesen, consultant MD, PhD, DMSc.^3^; Prof. Christian Mawrin, MD, Dr. Med, Dr. h.c.^4^; Martin Wirenfeldt Nielsen, MD, PhD^5^; Prof. Bjarne Winther Kristensen, MD, PhD^6^; Asger Sand Paludan-Müller, cand. Scient., PhD^7^; Associate Prof. Birgitte Brinkmann Olsen, MSc, PhD^8^ and Prof. Frantz Rom Poulsen, consultant, MD, PhD^1,2^

*^1^Department of Neurosurgery, Odense University Hospital, Odense, Denmark, Clinical Institute, University of Southern Denmark, Odense, Denmark.^2^BRIDGE (Brain Research ‑ Inter Disciplinary Guided Excellence), University of Southern Denmark, Odense, Denmark.^3^Department of Neurosurgery, Rigshospitalet, Copenhagen, Denmark, Copenhagen University.^4^Department of Neuropathology, Otto-von-Guericke University, Magdeburg, Germany.^5^Department of Pathology and Molecular Biology, Hospital South West Jutland, Esbjerg Denmark.^6^Department of Neuropathology, Rigshospitalet, Copenhagen, Denmark.^7^Nordic Cochrane Centre, Rigshospitalet, Copenhagen, Denmark, and Centre for Evidence-Based Medicine Odense (CEBMO).^8^Clinical Physiology and Nuclear Medicine, and Clinical Institute, Odense University Hospital, Odense, Denmark*

Table of Contents

[Abstract 3](#_Toc95766461)

[Introduction 4](#_Toc95766462)

[Rationale for review 4](#_Toc95766463)

[Scope and aim 4](#_Toc95766464)

[Review questions/objectives 4](#_Toc95766465)

[Background of meningiomas and animal models 4](#_Toc95766466)

[Epidemiology and a short overview of tumor characteristics 4](#_Toc95766467)

[Animal models 5](#_Toc95766468)

[**Genetically engineered models** 5](#_Toc95766469)

[**Patient-derived xenograft models** 6](#_Toc95766470)

[**Heterotopic vs. orthotopic** 6](#_Toc95766471)

[**Xenografted cell lines and models** 6](#_Toc95766472)

[Methods 7](#_Toc95766473)

[Current protocol development 7](#_Toc95766474)

[Criteria for considering studies for this review 7](#_Toc95766475)

[Information sources and search strategy 8](#_Toc95766476)

[Outcomes 8](#_Toc95766477)

[Data collection and management 8](#_Toc95766478)

[**Screening method** 8](#_Toc95766479)

[**Data collection process** 9](#_Toc95766480)

[**Data items and management** 9](#_Toc95766481)

[Analyses 9](#_Toc95766482)

[**Primary and secondary analyses** 9](#_Toc95766483)

[**Effect modifiers/study characteristics** 9](#_Toc95766484)

[**Effect measures - quantitative data** 9](#_Toc95766485)

[**Assessment of methodological quality included studies** 9](#_Toc95766486)

[**Synthesis of results** 11](#_Toc95766487)

[**Sensitivity analysis** 11](#_Toc95766488)

[Possible biases 11](#_Toc95766489)

[Dissemination plan 11](#_Toc95766490)

[Funding 11](#_Toc95766491)

[Systematic review protocol registration 11](#_Toc95766492)

[Contributions 11](#_Toc95766493)

[References 12](#_Toc95766494)

Past, Present and Future of Meningioma Animal Models – A Systematic Review

# **Abstract**

**Background:** Animal models are widely used to study pathological processes and drug effects/side effects in a controlled environment. For benign tumors, sustainable growth in animal models is notoriously difficult to obtain, with meningiomas being no exception. A variety of models, including xenografts and genetically engineered models (GEM), exist. Most widely used are the xenografted models from established cell lines. However, these are immortalized, which skews direct comparisons to human tumors. Some research groups have xenografted patient tumors into an animal model without using immortalization. However, this has proven difficult due to a lack of tumor take, and only a few have successfully implanted cells that grow into tumors. Currently, no systematic review exists on animal meningioma models which searches literature across multiple platforms.

**Objectives:** 1. to assess tumor take rate in the available model types: orthotopic and heterotopic xenografts using both established and patient-derived cell lines and GEM, 2. to search the literature for all available research regarding the different models and determine reproducibility and reliability alongside pros and cons, 3. to determine how models should be verified and which modalities that are necessary to compare xenograft to the parent tumor and how to assess GEM 4. to present possible future aspects of meningioma animal models in relation to optimal tests of future therapeutics in human meningiomas.

**Methods:** We will conduct a systematic review of in vivo studies examining meningioma models in animals. We will search Embase, Medline, and Web of Science to obtain literature. We aim to find unpublished data as well to include. We will include studies of xenografted models both based on established cell lines and xenografts straight from human tumors without immortalization, as well as GEM. Our primary outcomes are tumor induction rate and the validity of the different models. We will perform meta-analysis on tumor induction and subgroup analyses if deemed appropriate. The validity of the tumor models will be assessed qualitatively. Furthermore, chosen effect modifiers/study characteristics will be extracted and presented. We will assess the methodological quality on all included studies by evaluating cell and animal protocol transparency and replicability.

**Discussion:** By conducting a comprehensive systematic review including unpublished data (if possible), we expect to provide valuable information regarding all meningioma animal models – their induction rates, validity, uses, and applicability in a research setting. We expect this review to also assess pitfalls and successes in relation to establishing a xenograft model.

**Systematic review registration:** This protocol will be sent to PROSPERO (<https://www.crd.york.ac.uk/prospero/>) prior to full-text screening. This protocol will also be published on <https://www.medrxiv.org/> prior to full-text screening.

**Funding:** No funding related to the current systematic review protocol apply

# **Introduction**

## **Rationale for review**

A reproduceable and feasible meningioma animal model with close resemblance to the human meningioma to test new treatment modalities remains elusive. So far, few non-systematic reviews on the topic have been published(1-3). These well-written narrative reviews are prone to subjectivism due to their substantial reliance on the author’s pre-existing knowledge and experience. They do not typically present an unbiased, exhaustive, and systematic summary of a topic. Recently, a systematic review was conducted on mouse models in meningiomas(4). However, this study did not entail a search through multiple literature but used PubMed Central only. Additionally, the review did not include an assessment of methodological quality/risk of bias evaluation. Therefore, we intend to conduct a systematic review searching multiple databases, assess the methodological quality of included studies, and summarize results quantitatively or qualitatively, as appropriate.

In conclusion, we aim to provide the research community with a systematic review of tumor induction rates of all models and narratively describe the below-mentioned secondary outcomes. An additional goal of the systematic review is to assess which models can be used for what and why. And lastly, how the models compare to human tumors pathologically and in relation to test of treatments.

## **Scope and aim**

The aim of this systematic review is to search the literature on meningioma animal model modalities – to determine their uses, pros and cons, efficiency, reproducibility and their validity in relation to human tumors.

Additionally, we aim to identify and analyze knowledge gaps, which will lead to the identification of future research initiatives.

## **Review questions/objectives**

1. To assess tumor take rate in the available model types: orthotopic and heterotopic xenografts using both established and patient-derived cell lines.
2. To search the literature for all available research regarding the different models and assess reproducibility and reliability alongside pros and cons.
3. To determine how models should be verified and which modalities are necessary to compare xenograft or genetically modified tumors to the parent tumor.
4. To present possible future aspects of meningioma animal models in relation to optimal tests of future therapeutics in human meningiomas.

# **Background of meningiomas and animal models**

## **Epidemiology and a short overview of tumor characteristics**

Meningiomas are the second most frequent intracranial tumors after gliomas accounting for 13-26% of all intracranial neoplasms(5) and originate from arachnoidal cap cells. The estimated prevalence in the US population is 50.4-70.7/100.000 (6, 7)The incidence is approximately 1.9 for men and 4.5 for women per 100.000/year in a combined epidemiological study from Scandinavia (8, 9), similar to incidence rates in the US population of 3,7/100.000/year (6). The 2021 World Health Organization (WHO) classification categorizes meningiomas into three grades (benign, atypical and anaplastic) and 15 histopathological subtypes, where approximately 80% of meningiomas are benign (WHO grade 1) (10). The preferred treatment of symptomatic meningiomas is surgical resection (11). The extent of resection correlates strongly with the risk of tumor recurrence (11, 12), however there are multiple facets to recurrence rate (13-18). The Simpson Grade is adopted worldwide as a method to quantify the extent of resection (grade 1-5) where 1-3 are gross total resection with varying degrees of dural or/and bone resection with a symptomatic recurrence after 10 years between 9% (grade 1) and 29% (grade 3). Grades 4 and 5 represent subtotal resections with an estimated recurrence rate between 44-100% (11).

Meningiomas are complex and versatile in several aspects, from the smallest string of DNA to the macroscopic view in the operating theater. The molecular landscape alone has undergone vast changes in the past decade, where several molecular alterations have been discovered in meningiomas beside NF2 (encoding neurofibromin 2)(19-23). On an epigenetic level, DNA-methylation has shown much promise in prognostication, which differs from initial histology. Sahm et al. (24) showed six different meningioma classes based on their mutational, cytogenetic, and gene expression profiles (epigenetic factors). This new classification shows a better correlation with recurrence after surgery than the 2016 morphology-based WHO classification (25). Based on this work, the 2021 guidelines have adapted epigenetics for certain types of meningiomas. Histologically, there are 15 different subtypes of meningiomas(25). One of the most common is the meningothelial meningiomas WHO grade 1 tumor, which have spindly-looking cells with pink cytoplasm run in short fascicles, forming syncytial structures and whorls. The tumors can also express vascular structures, fibrous structures, psammoma bodies, microcysts in benign cases, and even cords of epithelioid cells and clear cells in atypical and malignant cases depending on the subtype(26). Immunohistochemically, almost 100% of meningiomas express somatostatin receptor 2A (SSTR2A) and epithelial membrane antigen (EMA)(27), but also other proteins and receptors, however not as specific, such as progesterone receptors in 56-83%(28-30).

## **Animal models**

Patient-derived orthotopic xenograft models in meningioma without immortalization remain an elusive tumor, especially from benign WHO grade 1 tumors. Reliable and reproducible tumor models are critical for understanding the pathophysiology and biology of meningiomas.

### **Genetically engineered models**

Developing in vivo models which truthfully represent human diseases is essential for validating new therapeutic approaches. Genetically engineered models (GEM) provide elaborate temporally and genetically controlled systems to investigate the cellular origins of tumors and gene function in tumorigenesis. Furthermore, they can prove to be valuable tools for testing targeted therapies. The models, however, contain innate weaknesses that may profoundly affect the use of these animals for predicting a patient’s response to a therapy (31) since only the gene of interest is investigated. The GEM have also shown disadvantages in a low tumor induction rate and a slow tumor development. The first GEM in meningiomas was based on loss of heterozygosity of the NF2 gene, which is found in 30-50% of sporadic meningiomas. Such a model was first described in 2002, where an NF2 knockout mouse model was accomplished by Cre-recombinase injection to induce meningiomas(32). However, the model is restricted in use since Cre (via adenoviral vectors) should be injected 2-3 days postnatally. They showed a limited tumor take rate of 19-29% with a long induction time 11-14 months. Even the addition of p53 mutation did not increase the rate of meningioma development; in addition to meningiomas and instead of, the mice developed multiple osteo- and fibrosarcomas. GEMs with loss of cyclin-dependent kinase inhibitor 2A/B (CDKN2A/B) alongside NF2 resulted in increased development of grade 2 and 3 meningiomas in mice(33, 34). A benign model expressing meningothelial and fibrous histological traits was developed using prostaglandin D2 synthase (PGDS) gene promotors (a specific marker of arachnoidal cells) to inactivate NF2, which resulted in a tumor take rate of 38% in mice(35). Furthermore, platelet-derived growth factor (PDGF) overexpression in arachnoidal cells induced meningiomas independent from NF2 inactivation(36).

### **Patient-derived xenograft models**

The GEMs are immunocompetent compared to the immunocompromised NMRI nu/nu type or ‘nude’ rodent model, a well-established method used to evaluate tumorigenicity and potential treatment efficacy(37). Researchers exploit the lack of T-cells in the athymic mice to create xenografts, e.g., with human cells, but this weakens the model due to loss of immune system involvement(37). The loss of T-cells and B-cells in SCID (severe compromised immunodeficiency) mice can be countered by humanization, which reduces many of the drawbacks, but HLA-1 and 2 restoration remain a challenge(38). Despite these issues, nude mouse models remain the preferred model for studies on whether a tumor will respond to a therapeutic agent(31).

### **Heterotopic vs. orthotopic**

Several studies have used the flank of nude mice for subcutaneous heterotopic transplantation of meningioma cells(39-45). However, it is still debated whether this can serve as a viable model due to lack of the natural environment. Gupta et al.(39) produced flank tumors using the established IOMM-LEE cell line, which is malignant. However, primary cell cultures from in-house patients all failed despite the use of Matrigel. Schrell et al. (40) implanted whole tumor pieces (0.5 ml) under the scalp – however, no induction rate was noted in the paper. Ragel et al. performed studies that showed growth in a flank model with benign cell lines (41, 42). Several groups have successfully reported orthotopic animal models especially with the use of established and malignant cell lines(43, 46-53). The induction rates are very high after transplantation of aggressive and manipulated meningioma cell lines(90-100%) in heterotopic and orthotopic mouse models(42, 52). Induction rates of benign tumors are slightly lower, ranging between 50% with cell suspensions(54) and 83% in heterotopic mouse models (55). Higher induction rates were observed when the tumors were placed subdurally (85%) compared to intraparenchymal placement (54%) (51). Aggressive meningioma types transplanted intracranially in an orthotopic mouse model reliably resulted in tumor formation (90-100%) in comparison to benign tumor cells transplanted into the brain parenchyma (56%) (51).

### **Xenografted cell lines and models**

Meningiomas are slow-growing tumors, and the cells are prone to cellular senescence hence the use of the cells is limited/restricted to early passages. Assurance of sustainable growth can be achieved using established cell lines, which have been thoroughly characterized using genomic profiles(56). It has been shown that immortalization by hTERT (telomerase catalytic subunit) expression and expression of human papillomavirus E6 and E7 oncogenes could overcome senescence (57). Furthermore, widely used cell lines have been established. Most of these are malignant, which in nature are more prone to sustainable and aggressive growth with a high induction rate in animal models. The IOMM-Lee cell line, which is the preferred malignant type (Anaplastic intraosseous meningioma WHO grade 3) (43, 50-52, 58-63) *(Gogineni 2011 (62) and Gupta 2011 (63) have since been retracted by the journals as late as 2021)*, was established by Lee in 1990(64) from a highly malignant and aggressive tumor, contains no loss of NF2. Other anaplastic cell lines such as KT21(65), F5(66), and CH-157 - the latter with an induction rate of >90% - have been established, all with a loss of 22q (location for NF2 gene)(50, 61, 67). The majority of meningiomas are benign, which is why a benign model is crucial. BenMen1 and HBL-52 (68, 69) from meningothelial meningioma WHO grade 1 tumor, have the advantage of containing both loss of 22q and hTERT expression, with the latter ensuring immortalization(70). Immortalization ensures growth by countering senescence. This however does not represent natural tumor pathogenesis in benign meningiomas. Xenografting patient-derived tumor material, including stroma without immortalization, optimally mimics the native environment and is, therefore, more suitable for assessing various drug therapies.

Few studies have successfully xenografted meningioma cells without the use of established or immortalized cell lines. As mentioned previously, benign meningioma cells are prone to senescence, which makes a patient-derived xenograft model without immortalization challenging. McCutcheon et al. implanted cells from malignant, atypical, and benign meningiomas. The atypical and malignant cells (including IOMM-Lee) had excellent tumor take. The benign tumors exhibited lower induction rates, ,i.e., subdural placement (at the skull base) showed an 85% induction rate vs. 54% in an intraparenchymal nude mouse model (51) – the xenografted tumors expressed similar histology and immunohistochemistry compared to the parent tumor. Friedrich et al. performed a study on cell cultures from 11 benign human meningiomas transplanted orthotopically the prefrontal cortex of nude mice without the use of immortalization. They used a similar cell protocol as McCutcheon et al.(51). They found 93% tumor take and histological similarities with the original tumor after three months (48), although EMA, which is present in almost 100% of meningiomas, was reduced in the meningioma cells after transplantation to 72% (48). This suggests that the absence of the immune system and the different microenvironment in rodents can change the expression profile of tumor cells. Furthermore, validated the model while testing the effect of systemic celecoxib on human meningiomas after intracranial transplantation into nude mice (71). A recent study from Zhang et al. (72) inoculated mice with cell cultures based on primary and recurrent meningiomas containing benign, atypical, and anaplastic (respectively WHO grade 1, 2 and 3). Only two of the tumors (primary and recurrent from the same patient, grade II and III respectively) were tumorigenic and tumor take rate of the two tumors were 76 % for the atypical and 71% for the anaplastic. Cells from five anaplastic and atypical meningiomas gave rise to zero tumors in the animals and the same applied to four WHO-grade 1 tumors. A major limitation in this study is the numbers of cells implanted, where only 100,000 cells were implanted compared to previous successful studies with 1,000,000 cells(48, 51).

In conclusion, the grade of the tumor is important for the induction rate. The malignant tumors are more prone to take in contrast to the benign models – unless they have been immortalized.

# **Methods**

## **Current protocol development**

This protocol and protocol abstract were developed using the PRISMA 2020 guidelines(73).

## **Criteria for considering studies for this review**

We will include original articles published in peer-reviewed journals with full texts, describing in vivo experiments with the intent to attain meningioma growth, either heterotopic and/or orthotopic, regardless of method.

We will exclude review articles, systematic reviews, humane studies, and conference abstracts. Furthermore, articles describing spontaneous meningiomas in animals are excluded without the use of genetic modification specifically aimed at meningiomas.

We will include articles of all languages, provided an English title or abstract is available. If an article in a language other than English is potentially eligible, we will have the full text translated if we are able.

## **Information sources and search strategy**

Search keywords and phrases for the search strategy were extracted through the cited literature in the background. Embase, Web of Science, and Medline were chosen after consultation with a librarian from the University of Southern Denmark. The first search in all databases was performed on June 18^th^, 2021. The search string has been evaluated by a librarian from the University of Southern Denmark. The literature search strings are available in **Appendix 1 – Search string.** Prior to submitting the systematic review, a second round of the search string will be performed spanning from June 18^th^, 2021, to submission.

Furthermore, we will seek to obtain non-published data if available from researchers working with meningioma animal models. We will also look at core literature and guidelines to assess missed literature by the search string and title/abstract screening.

## **Outcomes**

**Primary outcomes:**

- Induction rates of xenografted and GEM
- Validity of model (i.e. similarity to the human/parent tumor).
  - For xenografts and GEMs we will assess the methods used (histology, immunohistochemistry and other such as proteomics, next-generation sequencing and DNA-methylation)

**Secondary outcome for xenografted models:**

- Time until sacrifice/Tumor induction time

**Secondary outcome for GEM**

- Time until sacrifice/tumor induction time

## **Data collection and management**

### **Screening method**

All papers extracted via the search string will be screened by title and abstract by two authors (MSA+MK) in a blinded fashion. All disputes will be settled with senior author professor FRP. During the first round of screening (title and abstract), all papers which have been deemed eligible by either one of the authors (or both) will be included for the second round (full-text screening). Cohen’s Kappa statistics(74) will be used to assess inter-rater agreement between the two reviewers after the initial (title and abstract) screening. The first 200 papers will be rated un-blinded to adjust the screening method. Full-text screening will also be performed in a blinded fashion by two authors (MSA+MK) – Here both must agree to include. All disputes will also be settled via senior author professor FRP.

If the inter-rater agreement is below 0.8 (Kappa index) after the initial screening, we will include a third screening author to assess all excluded papers to increase sensitivity.

Exclusion priority list for both title/abstract and full-text screening: 1) Not animal study, 2) Not regarding meningiomas, 3) Spontaneous meningioma growth without the use of xenografts or genetically engineered models.

### **Data collection process**

Two individual reviewers (MSA+MK) will extract information in a blinded fashion. All disagreements will be resolved via the senior author (FRP).

### **Data items and management**

All papers are extracted to an MS Excel data collection form; the data collection form will be pilot-tested on randomly selected included studies before the full data extraction begins. Full datasets will be made available as supplementary to the final manuscript via the journal or researchgate.com. All screening will be made in MS Excel and marked relevant or not based on the abstract.

We will extract the following effect modifiers/study characteristics for xenografted model studies: Cell protocol, injection protocol, the concentration of cells, injection site, choice and number of animals for experiments, xenograft incubation time, interventions – What are the models used for?, methods used to compare parent tumor to xenografted tumor, similarities to parent tumor and tumor induction time

We will extract the following effect modifiers/study characteristics for GEM studies: Choice of model (which genetic lesion – Model protocol), time until sacrifice/tumor induction time, interventions – What are the models used for?, similarities to human meningiomas.

Extraction fields can be found in **Appendix 2 – Extraction fields**.

## **Analyses**

### **Primary and secondary analyses**

All analyses will be conducted in either the freely available software R and RStudio, The R Foundation for Statistical and Mathematics, Vienna, Austria, or STATA17, StataCorp LLC, Texas, USA.

**Primary outcomes**

- If studies are sufficiently similar to make meta-analysis appropriate, we will conduct a meta-analysis of induction rates for the different models. We will conduct a random-effects meta-analysis of single proportions using the metaprop function from the r-package meta. We will use the generalized linear mixed model for pooling proportions. If appropriate, we will examine potential heterogeneity with subgroup analyses (see synthesis of results). Given the risk of type 1 errors due to multiple testing issues, we will conservatively interpret findings from subgroup analyses.
- Validity of models/Similarities to human tumors are most likely heterogeneous and are therefore likely irrelevant for quantitative analyses. Hence, a narrative approach with overview tables will be performed. We expect the models (orthotopic, heterotopic xenografts, and GEM) to differ significantly in methods, so we will describe the pros and cons of the various types in the result section.

### **Effect modifiers/study characteristics**

We will present important potential effect modifiers and study characteristics in tables.

### **Effect measures - quantitative data**

- Tumor take rate [continuous data, percentage, mean difference]
- Duration of incubation/time until sacrifice [continuous data, time, mean difference]

### **Assessment of methodological quality included studies**

We will determine the methodological quality of all included studies – both reporting and methodological approach. As no validated tool exists for the purpose, we have developed our own inspired by Macleod et al.(75). Data included will contain the following – see list in this section: Study cell protocol – is it transparent, feasible and replicable? The same applies to cell concentration at inoculation, time of incubation, number of animals, and implantation method. This will be followed by a narrative description of the different aspects. All cell and animal protocols will be assessed by experts in their fields for transparency, feasibility and replicability/reproducible. See **Appendix 3** **– Assessment of methodological quality** for table and examples.

Investigators MSA+MK will separately assess methodological quality based on the points all discrepancies will be solved via third investigator senior author FRP: (1) Peer-reviewed, (2) Study cell protocol, (3) Animals, (4) Sample size calculation, (5) Surgical and implantation protocol^1^, (6) Compliance with animal welfare regulations, (7) Blinded assessment of outcome^2^, (8) All laboratory methods presented in results described, (9) Presentation of limitations, (10) Statement of potential conflict of interests

- ^1^GEM papers will be assessed based on how the genetic lesion was performed – Sufficient description or not.
- ^2^May not be applicable to non-intervention studies

All the above marked with a simple ‘X’, ‘XY’ or nothing. If no mark (or only X in XY columns), then a written description will be provided in supplementary material. Studies without sufficient quality of reporting in field 2, 3 and 5 will not be eligible for assessment of methodological approach (Y parts). Fields not applicable to study will be marked ‘NA’.

Points elaborated

1. Did the paper god through peer-review process?
2. Was the study’s cell protocol sufficiently described (transparent, reproducible) (X). And was the protocol feasible in relation to the experiment (Y)
3. Were the animals’ ‘demographic’ sufficiently described regarding type, age, numbers, weight (X). And were the animals chosen appropriate for the experiments (Y).
4. Did the authors perform a sample size calculation?
5. Were the surgical and implantation procedures sufficiently described – are they reproducible and transparent (X). And did the method seem feasible and well performed (Y)? (If applicable)
   1. GEM papers will not be applicable and will therefore not be assessed in this regard. Instead, GEM will be assessed based on how the genetic lesion was performed – Sufficient description or not.
6. Did the study comply with local, national, or international animal welfare regulations?
7. Did the study assess outcome blinded? This may not be applicable in non-intervention studies.
8. Were all the laboratory methods presented in results described? For instance, description of histological approach or next-generation sequencing protocol and alike
9. Did the study present limitations?
10. Did the study state potential conflict of interests?

If we come across studies with insufficient information, authors will be contacted – if possible. If we do not get a reply within a reasonable timeframe, this will be noted in the review.

- Points 1, 2 (X part), 3 (X part), 4, 5 (X part) 6, 7, 8, 9, 10 are all related to quality of reporting.
- Points 2 (Y part), 3 (Y part), 5 (Y part) are related to methodological approach.

We will summarize the overall quality of included studies narratively and include this in the interpretation of our results.

### **Synthesis of results**

As described above, we will perform a meta-analysis on tumor induction rates if appropriate. We will present forest plots and assess heterogeneity using the I^2^ measure. Additionally, we will assess potential heterogeneity by visually inspecting forest plots. Other data will be presented in tables and described narratively.

If heterogeneity is identified, we will examine this by conducting sub-group analyses. Individual factors which may affect induction rate in xenografted models: number of cells, cell concentration, the passage in which the cells were implanted, implantation time during the operation of the animals, parent tumor grade (WHO grade I-III), animal type, and incubation time. Individual factors that may affect the induction rate in GEM: Genomic lesion type and incubation time.

We will describe the validity of the different tumor models and their relation to human tumors narratively, including the methodological quality of included studies in the narrative. Furthermore, tables will be presented of methodological approach according to **Appendix 3** **– Assessment of methodological quality**.

### **Sensitivity analysis**

None have been pre-specified due to issues suitable for sensitivity analysis have not been identified. Eventual issues of the studies will be identified during the review process.

## **Possible biases**

Publication bias: We will create funnel plots to try and determine whether there is any indication of publication bias.

Selective outcome reporting/reporting bias assessment: Studies might not report all outcome data or all analyses performed. It is only possible to assess this if we have access to the original protocols. We expect this to only rarely be the case. Therefore, it is likely impossible to determine the risk of selective outcome reporting, and we will interpret our results with caution.

## **Dissemination plan**

A paper will be submitted to a leading journal of the field. Furthermore, abstracts will be sent to appropriate international conferences.

Furthermore, author MSA will update the review every 3-5 years with the latest studies on the topic.

# **Funding**

No funding related to current systematic review apply.

# **Systematic review protocol registration**

This protocol will be sent to PROSPERO (<https://www.crd.york.ac.uk/prospero/>) prior to full-text screening. This protocol will also be published on <https://www.medrxiv.org/>.

# **Contributions**

- *Mikkel Schou Andersen: Conceptualization, methodology, writing, screening, full-text data extraction, analyses.*
- *Frantz Rom Poulsen: Conceptualization, methodology, review and editing, supervision*
- *Bo Halle: Writing, animal protocol assessment – Review and editing*
- *Christian Bonde Pedersen: Writing – review and editing*
- *Tiit Mathiesen: Writing – review and editing*
- *Christian Mawrin: Writing – review and editing*
- *Martin Wirenfeldt Nielsen: Writing – review and editing*
- *Bjarne W Kristensen: Writing – review and editing*
- *Birgitte Brinkmann Olsen: Writing, cell-protocol assessment - review and editing*
- *Mikkel S Kofoed: Writing, screening, full-text data extraction – review and editing*
- *Asger S Paludan-Müller: Methodology, writing – review and editing*

# **References**

1. Mawrin C. Animal models of meningiomas. Chin Clin Oncol. 2017.

2. Kalamarides M, Peyre M, Giovannini M. Meningioma mouse models. J Neurooncol. 2010;99(3):325-31.

3. Cimino PJ. Malignant progression to anaplastic meningioma: Neuropathology, molecular pathology, and experimental models. Exp Mol Pathol. 2015;99(2):354-9.

4. Boetto J, Peyre M, Kalamarides M. Mouse Models in Meningioma Research: A Systematic Review. Cancers (Basel). 2021;13(15).

5. Marosi C, Hassler M, Roessler K, Reni M, Sant M, Mazza E, et al. Meningioma. Crit Rev Oncol Hematol. 2008;67(2):153-71.

6. Davis FG, Kupelian V, Freels S, McCarthy B, Surawicz T. Prevalence estimates for primary brain tumors in the United States by behavior and major histology groups. Neuro Oncol. 2001;3(3):152-8.

7. Porter KR, McCarthy BJ, Freels S, Kim Y, Davis FG. Prevalence estimates for primary brain tumors in the United States by age, gender, behavior, and histology. Neuro Oncol. 2010;12(6):520-7.

8. Klaeboe L, Lonn S, Scheie D, Auvinen A, Christensen HC, Feychting M, et al. Incidence of intracranial meningiomas in Denmark, Finland, Norway and Sweden, 1968-1997. Int J Cancer. 2005;117(6):996-1001.

9. Deltour I, Johansen C, Auvinen A, Feychting M, Klaeboe L, Schuz J. Time trends in brain tumor incidence rates in Denmark, Finland, Norway, and Sweden, 1974-2003. J Natl Cancer Inst. 2009;101(24):1721-4.

10. Louis DN, Perry A, Wesseling P, Brat DJ, Cree IA, Figarella-Branger D, et al. The 2021 WHO Classification of Tumors of the Central Nervous System: a summary. Neuro Oncol. 2021;23(8):1231-51.

11. Simpson D. The recurrence of intracranial meningiomas after surgical treatment. J Neurol Neurosurg Psychiatry. 1957;20(1):22-39.

12. Gousias K, Schramm J, Simon M. The Simpson grading revisited: aggressive surgery and its place in modern meningioma management. J Neurosurg. 2016;125(3):551-60.

13. Chiba K, Sugawara T, Kobayashi D, Sato A, Murota Y, Maehara T. Atypical Histological Features as Risk Factors for Recurrence in Newly Diagnosed WHO Grade I Meningioma. Neurol Med Chir (Tokyo). 2021;61(11):647-51.

14. Nanda A, Bir SC, Konar S, Maiti T, Kalakoti P, Jacobsohn JA, et al. Outcome of resection of WHO Grade II meningioma and correlation of pathological and radiological predictive factors for recurrence. J Clin Neurosci. 2016;31:112-21.

15. Biczok A, Karschnia P, Vitalini R, Lenski M, Greve T, Thorsteinsdottir J, et al. Past medical history of tumors other than meningioma is a negative prognostic factor for tumor recurrence in meningiomas WHO grade I. Acta Neurochir (Wien). 2021;163(10):2853-9.

16. Gallagher MJ, Jenkinson MD, Brodbelt AR, Mills SJ, Chavredakis E. WHO grade 1 meningioma recurrence: Are location and Simpson grade still relevant? Clin Neurol Neurosurg. 2016;141:117-21.

17. Hwang WL, Marciscano AE, Niemierko A, Kim DW, Stemmer-Rachamimov AO, Curry WT, et al. Imaging and extent of surgical resection predict risk of meningioma recurrence better than WHO histopathological grade. Neuro Oncol. 2016;18(6):863-72.

18. Haddad AF, Young JS, Kanungo I, Sudhir S, Chen JS, Raleigh DR, et al. WHO Grade I Meningioma Recurrence: Identifying High Risk Patients Using Histopathological Features and the MIB-1 Index. Front Oncol. 2020;10:1522.

19. Clark VE, Harmanci AS, Bai H, Youngblood MW, Lee TI, Baranoski JF, et al. Recurrent somatic mutations in POLR2A define a distinct subset of meningiomas. Nat Genet. 2016;48(10):1253-9.

20. Clark VE, Erson-Omay EZ, Serin A, Yin J, Cotney J, Ozduman K, et al. Genomic analysis of non-NF2 meningiomas reveals mutations in TRAF7, KLF4, AKT1, and SMO. Science. 2013;339(6123):1077-80.

21. Brastianos PK, Horowitz PM, Santagata S, Jones RT, McKenna A, Getz G, et al. Genomic sequencing of meningiomas identifies oncogenic SMO and AKT1 mutations. Nat Genet. 2013;45(3):285-9.

22. Yuzawa S, Nishihara H, Tanaka S. Genetic landscape of meningioma. Brain Tumor Pathol. 2016;33(4):237-47.

23. Bi WL, Greenwald NF, Abedalthagafi M, Wala J, Gibson WJ, Agarwalla PK, et al. Erratum: Genomic landscape of high-grade meningiomas. NPJ Genom Med. 2017;2:26.

24. Sahm F, Schrimpf D, Stichel D, Jones DTW, Hielscher T, Schefzyk S, et al. DNA methylation-based classification and grading system for meningioma: a multicentre, retrospective analysis. Lancet Oncol. 2017;18(5):682-94.

25. Louis DN, Perry A, Reifenberger G, von Deimling A, Figarella-Branger D, Cavenee WK, et al. The 2016 World Health Organization Classification of Tumors of the Central Nervous System: a summary. Acta Neuropathol. 2016;131(6):803-20.

26. Backer-Grondahl T, Moen BH, Torp SH. The histopathological spectrum of human meningiomas. Int J Clin Exp Pathol. 2012;5(3):231-42.

27. Boulagnon-Rombi C, Fleury C, Fichel C, Lefour S, Marchal Bressenot A, Gauchotte G. Immunohistochemical Approach to the Differential Diagnosis of Meningiomas and Their Mimics. J Neuropathol Exp Neurol. 2017;76(4):289-98.

28. Carroll RS, Glowacka D, Dashner K, Black PM. Progesterone receptor expression in meningiomas. Cancer Res. 1993;53(6):1312-6.

29. Hsu DW, Efird JT, Hedley-Whyte ET. Progesterone and estrogen receptors in meningiomas: prognostic considerations. J Neurosurg. 1997;86(1):113-20.

30. Iplikcioglu AC, Hatiboglu MA, Ozek E, Ozcan D. Is progesteron receptor status really a prognostic factor for intracranial meningiomas? Clin Neurol Neurosurg. 2014;124:119-22.

31. Richmond A, Su Y. Mouse xenograft models vs GEM models for human cancer therapeutics. Dis Model Mech. 2008;1(2-3):78-82.

32. Kalamarides M, Niwa-Kawakita M, Leblois H, Abramowski V, Perricaudet M, Janin A, et al. Nf2 gene inactivation in arachnoidal cells is rate-limiting for meningioma development in the mouse. Genes Dev. 2002;16(9):1060-5.

33. Peyre M, Stemmer-Rachamimov A, Clermont-Taranchon E, Quentin S, El-Taraya N, Walczak C, et al. Meningioma progression in mice triggered by Nf2 and Cdkn2ab inactivation. Oncogene. 2012;32:4264.

34. Kalamarides M, Stemmer-Rachamimov AO, Takahashi M, Han ZY, Chareyre F, Niwa-Kawakita M, et al. Natural history of meningioma development in mice reveals: a synergy of Nf2 and p16(Ink4a) mutations. Brain Pathol. 2008;18(1):62-70.

35. Kalamarides M, Stemmer-Rachamimov AO, Niwa-Kawakita M, Chareyre F, Taranchon E, Han ZY, et al. Identification of a progenitor cell of origin capable of generating diverse meningioma histological subtypes. Oncogene. 2011;30(20):2333-44.

36. Peyre M, Salaud C, Clermont-Taranchon E, Niwa-Kawakita M, Goutagny S, Mawrin C, et al. PDGF activation in PGDS-positive arachnoid cells induces meningioma formation in mice promoting tumor progression in combination with Nf2 and Cdkn2ab loss. Oncotarget. 2015;6(32):32713-22.

37. Shultz LD, Goodwin N, Ishikawa F, Hosur V, Lyons BL, Greiner DL. Human cancer growth and therapy in immunodeficient mouse models. Cold Spring Harb Protoc. 2014;2014(7):694-708.

38. Bernard D, Peakman M, Hayday AC. Establishing humanized mice using stem cells: maximizing the potential. Clin Exp Immunol. 2008;152(3):406-14.

39. Gupta V, Su YS, Samuelson CG, Liebes LF, Chamberlain MC, Hofman FM, et al. Irinotecan: a potential new chemotherapeutic agent for atypical or malignant meningiomas. J Neurosurg. 2007;106(3):455-62.

40. Schrell UM, Rittig MG, Anders M, Kiesewetter F, Marschalek R, Koch UH, et al. Hydroxyurea for treatment of unresectable and recurrent meningiomas. I. Inhibition of primary human meningioma cells in culture and in meningioma transplants by induction of the apoptotic pathway. J Neurosurg. 1997;86(5):845-52.

41. Ragel BT, Gillespie DL, Kushnir V, Polevaya N, Kelly D, Jensen RL. Calcium channel antagonists augment hydroxyurea- and ru486-induced inhibition of meningioma growth in vivo and in vitro. Neurosurgery. 2006;59(5):1109-20; discussion 20-1.

42. Ragel BT, Jensen RL, Gillespie DL, Prescott SM, Couldwell WT. Celecoxib inhibits meningioma tumor growth in a mouse xenograft model. Cancer. 2007;109(3):588-97.

43. Das A, Alshareef M, Martinez Santos JL, Porto GBF, McDonald DG, Infinger LK, et al. Evaluating anti-tumor activity of palbociclib plus radiation in anaplastic and radiation-induced meningiomas: pre-clinical investigations. Clin Transl Oncol. 2020;22(11):2017-25.

44. Takeda H, Okada M, Kuramoto K, Suzuki S, Sakaki H, Sanomachi T, et al. Antitumor activity of gemcitabine against high-grade meningioma in vitro and in vivo. Oncotarget. 2017;8(53):90996-1008.

45. Jiang C, Song T, Li J, Ao F, Gong X, Lu Y, et al. RAS Promotes Proliferation and Resistances to Apoptosis in Meningioma. Mol Neurobiol. 2017;54(1):779-87.

46. Cargioli TG, Ugur HC, Ramakrishna N, Chan J, Black PM, Carroll RS. Establishment of an in vivo meningioma model with human telomerase reverse transcriptase. Neurosurgery. 2007;60(4):750-9; discussion 9-60.

47. Michelhaugh SK, Guastella AR, Varadarajan K, Klinger NV, Parajuli P, Ahmad A, et al. Development of patient-derived xenograft models from a spontaneously immortal low-grade meningioma cell line, KCI-MENG1. J Transl Med. 2015;13:227.

48. Friedrich S, Schwabe K, Klein R, Krusche CA, Krauss JK, Nakamura M. Comparative morphological and immunohistochemical study of human meningioma after intracranial transplantation into nude mice. J Neurosci Methods. 2012;205(1):1-9.

49. Nigim F, Esaki S, Hood M, Lelic N, James MF, Ramesh V, et al. A new patient-derived orthotopic malignant meningioma model treated with oncolytic herpes simplex virus. Neuro Oncol. 2016;18(9):1278-87.

50. Ragel BT, Elam IL, Gillespie DL, Flynn JR, Kelly DA, Mabey D, et al. A novel model of intracranial meningioma in mice using luciferase-expressing meningioma cells. Laboratory investigation. J Neurosurg. 2008;108(2):304-10.

51. McCutcheon IE, Friend KE, Gerdes TM, Zhang BM, Wildrick DM, Fuller GN. Intracranial injection of human meningioma cells in athymic mice: an orthotopic model for meningioma growth. J Neurosurg. 2000;92(2):306-14.

52. Baia GS, Dinca EB, Ozawa T, Kimura ET, McDermott MW, James CD, et al. An orthotopic skull base model of malignant meningioma. Brain Pathol. 2008;18(2):172-9.

53. Nigim F, Kiyokawa J, Gurtner A, Kawamura Y, Hua L, Kasper EM, et al. A Monoclonal Antibody Against beta1 Integrin Inhibits Proliferation and Increases Survival in an Orthotopic Model of High-Grade Meningioma. Target Oncol. 2019;14(4):479-89.

54. Malham GM, Thomsen RJ, Synek BJ, Baguley BC. Establishment of primary human meningiomas as subcutaneous xenografts in mice. Br J Neurosurg. 2001;15(4):328-34.

55. Medhkour A, Van Roey M, Sobel RA, Fingert HJ, Lee J, Martuza RL. Implantation of human meningiomas into the subrenal capsule of the nude mouse. A model for studies of tumor growth. J Neurosurg. 1989;71(4):545-50.

56. Mei Y, Bi WL, Greenwald NF, Agar NY, Beroukhim R, Dunn GP, et al. Genomic profile of human meningioma cell lines. PLoS One. 2017;12(5):e0178322.

57. Baia GS, Slocum AL, Hyer JD, Misra A, Sehati N, VandenBerg SR, et al. A genetic strategy to overcome the senescence of primary meningioma cell cultures. J Neurooncol. 2006;78(2):113-21.

58. Wilisch-Neumann A, Kliese N, Pachow D, Schneider T, Warnke JP, Braunsdorf WE, et al. The integrin inhibitor cilengitide affects meningioma cell motility and invasion. Clin Cancer Res. 2013;19(19):5402-12.

59. Tuchen M, Wilisch-Neumann A, Daniel EA, Baldauf L, Pachow D, Scholz J, et al. Receptor tyrosine kinase inhibition by regorafenib/sorafenib inhibits growth and invasion of meningioma cells. Eur J Cancer. 2017;73:9-21.

60. Pachow D, Andrae N, Kliese N, Angenstein F, Stork O, Wilisch-Neumann A, et al. mTORC1 inhibitors suppress meningioma growth in mouse models. Clin Cancer Res. 2013;19(5):1180-9.

61. Gogineni VR, Nalla AK, Gupta R, Dinh DH, Klopfenstein JD, Rao JS. Chk2-mediated G2/M cell cycle arrest maintains radiation resistance in malignant meningioma cells. Cancer Lett. 2011;313(1):64-75.

62. Gogineni VR, Nalla AK, Gupta R, Gujrati M, Klopfenstein JD, Mohanam S, et al. alpha3beta1 integrin promotes radiation-induced migration of meningioma cells. Int J Oncol. 2011;38(6):1615-24.

63. Gupta R, Nalla AK, Gogineni VR, Chetty C, Bhoopathi P, Klopfenstein JD, et al. uPAR/cathepsin B overexpression reverse angiogenesis by rescuing FAK phosphorylation in uPAR/cathepsin B down regulated meningioma. PLoS One. 2011;6(2):e17123.

64. Lee WH. Characterization of a newly established malignant meningioma cell line of the human brain: IOMM-Lee. Neurosurgery. 1990;27(3):389-95; discussion 96.

65. Tanaka K, Sato C, Maeda Y, Koike M, Matsutani M, Yamada K, et al. Establishment of a human malignant meningioma cell line with amplified c-myc oncogene. Cancer. 1989;64(11):2243-9.

66. Yazaki T, Takamiya Y, Costello PC, Mineta T, Menon AG, Rabkin SD, et al. Inhibition of angiogenesis and growth of human non-malignant and malignant meningiomas by TNP-470. J Neurooncol. 1995;23(1):23-9.

67. La Cava F, Fringuello Mingo A, Irrera P, Di Vito A, Cordaro A, Brioschi C, et al. Orthotopic induction of CH157MN convexity and skull base meningiomas into nude mice using stereotactic surgery and MRI characterization. Animal Model Exp Med. 2019;2(1):58-63.

68. Akat K, Mennel HD, Kremer P, Gassler N, Bleck CK, Kartenbeck J. Molecular characterization of desmosomes in meningiomas and arachnoidal tissue. Acta Neuropathol. 2003;106(4):337-47.

69. Akat K, Bleck CK, Lee YM, Haselmann-Weiss U, Kartenbeck J. Characterization of a novel type of adherens junction in meningiomas and the derived cell line HBL-52. Cell Tissue Res. 2008;331(2):401-12.

70. Puttmann S, Senner V, Braune S, Hillmann B, Exeler R, Rickert CH, et al. Establishment of a benign meningioma cell line by hTERT-mediated immortalization. Lab Invest. 2005;85(9):1163-71.

71. Friedrich S, Schwabe K, Grote M, Krauss JK, Nakamura M. Effect of systemic celecoxib on human meningioma after intracranial transplantation into nude mice. Acta Neurochir (Wien). 2013;155(1):173-82.

72. Zhang H, Qi L, Du Y, Huang LF, Braun FK, Kogiso M, et al. Patient-Derived Orthotopic Xenograft (PDOX) Mouse Models of Primary and Recurrent Meningioma. Cancers (Basel). 2020;12(6).

73. Page MJ, McKenzie JE, Bossuyt PM, Boutron I, Hoffmann TC, Mulrow CD, et al. The PRISMA 2020 statement: an updated guideline for reporting systematic reviews. BMJ. 2021;372:n71.

74. McHugh ML. Interrater reliability: the kappa statistic. Biochem Med (Zagreb). 2012;22(3):276-82.

75. Macleod MR, O'Collins T, Howells DW, Donnan GA. Pooling of animal experimental data reveals influence of study design and publication bias. Stroke. 2004;35(5):1203-8.
