# Supplementary material for: Meningioma animal models: a systematic review and meta-analysis
Source: J Transl Med. 2023 Oct 28;21:764. doi: 10.1186/s12967-023-04620-7 (PMC10612271; doi:10.1186/s12967-023-04620-7)
Supplement: Supplementary file 4 — Additional file 4: Protocol breaches. [file 12967_2023_4620_MOESM4_ESM.docx]

Protocol breaches

Minor

- Assessment of methodological approach:
  - (X) and/or (Y) can be given in situations where only minor data is missing, i.e. weight of animals or unclear duration prior to treatment, however tumor take is verified via non-invasive modality.
  - Item five now include the experiment as a whole (with animals as a separate Item 3 as before). This means the surgical procedure; implantation method and duration all must be described sufficiently (transparent and replicable) for a full X.
- Study inclusions - Added two categories:
  - Uncategorized: Articles not eligible for either of the groups ECLM, PTM or GEM in an analyses sense
  - Historical perspective: Older literature (>40 years old) with unclear methods and results were excluded from final analyses
- Added additional inspiration source for data extraction and assessment of methodological approach (ARRIVE 2.0)
- Data extraction:
  - Added WHO grade as an extraction item for Primary Tumor Models (patient-derived)
  - Added Tumor take rate for other tumors than meningiomas as an extraction item for Genetically Engineered Models
- Funnel plot regarding publication bias not created for manuscript.

Major

- Critical appraisal
  - Added items from SYRCLE’s Risk of Bias in CRIME-Q tool.
