# Supplementary material for: Meningioma animal models: a systematic review and meta-analysis
Source: J Transl Med. 2023 Oct 28;21:764. doi: 10.1186/s12967-023-04620-7 (PMC10612271; doi:10.1186/s12967-023-04620-7)

**Supplemental Material 5 -**

**Literature search string**

**Database:**
Embase Classic+Embase <1947 to 2021 June 18>

| **#** | **Query** | **Results from 21 Jun 2021** |
| --- | --- | --- |
| 1 | exp animal model/ | 1,495,232 |
| 2 | exp animal experiment/ | 2,718,328 |
| 3 | exp tumor xenograft/ | 62,562 |
| 4 | exp cell transplantation/ | 185,831 |
| 5 | exp orthotopic transplantation/ | 2,738 |
| 6 | exp heterotopic transplantation/ | 1,377 |
| 7 | exp genetic engineering/ | 93,538 |
| 8 | exp mouse/ | 2,056,433 |
| 9 | animal model*.mp. | 1,571,184 |
| 10 | animal experiment*.mp. | 2,713,095 |
| 11 | tumor xenograft.mp. | 62,921 |
| 12 | tumour xenograft.mp. | 473 |
| 13 | nf2 model*.mp. | 1 |
| 14 | cell transplantation.mp. | 198,048 |
| 15 | orthotopic transplantation.mp. | 3,576 |
| 16 | heterotopic transplantation.mp. | 1,736 |
| 17 | genetically modified.mp. | 21,504 |
| 18 | genetically engineered.mp. | 19,167 |
| 19 | athymic.mp. | 14,421 |
| 20 | orthotopic.mp. | 45,681 |
| 21 | heterotopic.mp. | 23,083 |
| 22 | transplantation.mp. | 932,135 |
| 23 | exp meningioma/ | 35,700 |
| 24 | mening?oma*.mp. | 41,412 |
| 25 | 1 or 2 or 3 or 4 or 5 or 6 or 7 or 8 or 9 or 10 or 11 or 12 or 13 or 14 or 15 or 16 or 17 or 18 or 19 or 20 or 21 or 22 | 4,912,581 |
| 26 | 23 or 24 | 41,412 |
| 27 | 25 and 26 | 1,127 |

exp animal model/
exp animal experiment/
exp tumor xenograft/
exp cell transplantation/
exp orthotopic transplantation/
exp heterotopic transplantation/
exp genetic engineering/
exp mouse/
animal model*.mp.
animal experiment*.mp.
tumor xenograft.mp.
tumour xenograft.mp.
nf2 model*.mp.
cell transplantation.mp.
orthotopic transplantation.mp.
heterotopic transplantation.mp.
genetically modified.mp.
genetically engineered.mp.
athymic.mp.
orthotopic.mp.
heterotopic.mp.
transplantation.mp.
exp meningioma/
mening?oma*.mp.
1 or 2 or 3 or 4 or 5 or 6 or 7 or 8 or 9 or 10 or 11 or 12 or 13 or 14 or 15 or 16 or 17 or 18 or 19 or 20 or 21 or 22
23 or 24
25 and 26


https://proxy1-bib.sdu.dk:2048/login?url=https://ovidsp.ovid.com/ovidweb.cgi?T=JS&NEWS=N&PAGE=main&SHAREDSEARCHID=1dN8K0miQcRWQ5w2Vd2yTEa4Wm2HHvMDNmJtLP9zBxsc6Y8TmRIoOBav7PeMxn6Dw

**Database:**
Ovid MEDLINE(R) ALL <1946 to June 18, 2021>

| **#** | **Query** | **Results from 21 Jun 2021** |
| --- | --- | --- |
| 1 | exp animal model/ | 596,233 |
| 2 | exp animal experiment/ | 9,712 |
| 3 | exp tumor xenograft/ | 40,220 |
| 4 | exp cell transplantation/ | 102,907 |
| 5 | Animals/ or orthotopic transplantation.mp. or Mice/ | 6,851,146 |
| 6 | orthotopic.mp. | 28,943 |
| 7 | heterotopic transplantation.mp. or exp Transplantation, Heterotopic/ | 3,678 |
| 8 | exp genetic engineering/ | 213,960 |
| 9 | exp mouse/ | 1,630,395 |
| 10 | animal model.mp. | 83,337 |
| 11 | animal experiment.mp. | 3,489 |
| 12 | tumor xenograft.mp. | 3,765 |
| 13 | tumour xenograft.mp. | 324 |
| 14 | nf2 model.mp. | 1 |
| 15 | cell transplantation.mp. | 115,489 |
| 16 | genetically modified.mp. | 76,159 |
| 17 | Animals/ or Disease Models, Animal/ or genetically engineered.mp. or Genetic Engineering/ or Mice/ | 6,871,222 |
| 18 | athymic.mp. | 11,124 |
| 19 | transplantation.mp. | 729,262 |
| 20 | exp meningioma/ | 19,824 |
| 21 | mening?oma*.mp. | 27,063 |
| 22 | 1 or 2 or 3 or 4 or 5 or 6 or 7 or 8 or 9 or 10 or 11 or 12 or 13 or 14 or 15 or 16 or 17 or 18 or 19 | 7,527,142 |
| 23 | 20 or 21 | 27,063 |
| 24 | 22 and 23 | 1,266 |

exp animal model/
exp animal experiment/
exp tumor xenograft/
exp cell transplantation/
Animals/ or orthotopic transplantation.mp. or Mice/
orthotopic.mp.
heterotopic transplantation.mp. or exp Transplantation, Heterotopic/
exp genetic engineering/
exp mouse/
animal model.mp.
animal experiment.mp.
tumor xenograft.mp.
tumour xenograft.mp.
nf2 model.mp.
cell transplantation.mp.
genetically modified.mp.
Animals/ or Disease Models, Animal/ or genetically engineered.mp. or Genetic Engineering/ or Mice/
athymic.mp.
transplantation.mp.
exp meningioma/
mening?oma*.mp.
1 or 2 or 3 or 4 or 5 or 6 or 7 or 8 or 9 or 10 or 11 or 12 or 13 or 14 or 15 or 16 or 17 or 18 or 19
20 or 21
22 and 23


<https://proxy1-bib.sdu.dk:2048/login?url=https://ovidsp.ovid.com/ovidweb.cgi?T=JS&NEWS=N&PAGE=main&SHAREDSEARCHID=nTFQBXJz75Se8MDXE3vobPZwAfxKTnYzDVAvbsSOS6w4UXWbqdkqiyyLOnqMIdv1>

Web of Science 21-06-21

Search History

                                                                                                                                                                                                                                                                    Web of Science Core Collection

| Set | Results |  |
| --- | --- | --- |
| # 22 | [**488**](http://apps.webofknowledge.com.proxy1-bib.sdu.dk:2048/summary.do?product=WOS&doc=1&qid=24&SID=C64CBWXwzZ82V6xabFJ&search_mode=CombineSearches&update_back2search_link_param=yes) | #21 AND #17  *Indexes=SCI-EXPANDED, SSCI, A&HCI, CPCI-S, CPCI-SSH, ESCI Timespan=All years* |
| # 21 | [**23,100**](http://apps.webofknowledge.com.proxy1-bib.sdu.dk:2048/summary.do?product=WOS&doc=1&qid=23&SID=C64CBWXwzZ82V6xabFJ&search_mode=CombineSearches&update_back2search_link_param=yes) | #20 OR #19 OR #18  *Indexes=SCI-EXPANDED, SSCI, A&HCI, CPCI-S, CPCI-SSH, ESCI Timespan=All years* |
| # 20 | [**23,086**](http://apps.webofknowledge.com.proxy1-bib.sdu.dk:2048/summary.do?product=WOS&doc=1&qid=22&SID=C64CBWXwzZ82V6xabFJ&search_mode=AdvancedSearch&update_back2search_link_param=yes) | ALL=Meningiomas  *Indexes=SCI-EXPANDED, SSCI, A&HCI, CPCI-S, CPCI-SSH, ESCI Timespan=All years* |
| # 19 | [**7**](http://apps.webofknowledge.com.proxy1-bib.sdu.dk:2048/summary.do?product=WOS&doc=1&qid=21&SID=C64CBWXwzZ82V6xabFJ&search_mode=AdvancedSearch&update_back2search_link_param=yes) | ALL=meningeom  *Indexes=SCI-EXPANDED, SSCI, A&HCI, CPCI-S, CPCI-SSH, ESCI Timespan=All years* |
| # 18 | [**23,080**](http://apps.webofknowledge.com.proxy1-bib.sdu.dk:2048/summary.do?product=WOS&doc=1&qid=20&SID=C64CBWXwzZ82V6xabFJ&search_mode=AdvancedSearch&update_back2search_link_param=yes) | ALL=Meningioma  *Indexes=SCI-EXPANDED, SSCI, A&HCI, CPCI-S, CPCI-SSH, ESCI Timespan=All years* |
| # 17 | [**2,885,564**](http://apps.webofknowledge.com.proxy1-bib.sdu.dk:2048/summary.do?product=WOS&doc=1&qid=19&SID=C64CBWXwzZ82V6xabFJ&search_mode=CombineSearches&update_back2search_link_param=yes) | #16 OR #15 OR #14 OR #13 OR #12 OR #11 OR #10 OR #9 OR #8 OR #7 OR #6 OR #5 OR #4 OR #3 OR #2 OR #1  *Indexes=SCI-EXPANDED, SSCI, A&HCI, CPCI-S, CPCI-SSH, ESCI Timespan=All years* |
| # 16 | [**1,687,178**](http://apps.webofknowledge.com.proxy1-bib.sdu.dk:2048/summary.do?product=WOS&doc=1&qid=18&SID=C64CBWXwzZ82V6xabFJ&search_mode=AdvancedSearch&update_back2search_link_param=yes) | ALL=mice  *Indexes=SCI-EXPANDED, SSCI, A&HCI, CPCI-S, CPCI-SSH, ESCI Timespan=All years* |
| # 15 | [**1,686,918**](http://apps.webofknowledge.com.proxy1-bib.sdu.dk:2048/summary.do?product=WOS&doc=1&qid=17&SID=C64CBWXwzZ82V6xabFJ&search_mode=AdvancedSearch&update_back2search_link_param=yes) | ALL=mouse  *Indexes=SCI-EXPANDED, SSCI, A&HCI, CPCI-S, CPCI-SSH, ESCI Timespan=All years* |
| # 14 | [**684,091**](http://apps.webofknowledge.com.proxy1-bib.sdu.dk:2048/summary.do?product=WOS&doc=1&qid=15&SID=C64CBWXwzZ82V6xabFJ&search_mode=AdvancedSearch&update_back2search_link_param=yes) | ALL=transplantation  *Indexes=SCI-EXPANDED, SSCI, A&HCI, CPCI-S, CPCI-SSH, ESCI Timespan=All years* |
| # 13 | [**21,887**](http://apps.webofknowledge.com.proxy1-bib.sdu.dk:2048/summary.do?product=WOS&doc=1&qid=14&SID=C64CBWXwzZ82V6xabFJ&search_mode=AdvancedSearch&update_back2search_link_param=yes) | ALL=genetically engineered  *Indexes=SCI-EXPANDED, SSCI, A&HCI, CPCI-S, CPCI-SSH, ESCI Timespan=All years* |
| # 12 | [**30,750**](http://apps.webofknowledge.com.proxy1-bib.sdu.dk:2048/summary.do?product=WOS&doc=1&qid=13&SID=C64CBWXwzZ82V6xabFJ&search_mode=AdvancedSearch&update_back2search_link_param=yes) | ALL=genetically modified  *Indexes=SCI-EXPANDED, SSCI, A&HCI, CPCI-S, CPCI-SSH, ESCI Timespan=All years* |
| # 11 | [**405**](http://apps.webofknowledge.com.proxy1-bib.sdu.dk:2048/summary.do?product=WOS&doc=1&qid=12&SID=C64CBWXwzZ82V6xabFJ&search_mode=AdvancedSearch&update_back2search_link_param=yes) | ALL=NF2 model  *Indexes=SCI-EXPANDED, SSCI, A&HCI, CPCI-S, CPCI-SSH, ESCI Timespan=All years* |
| # 10 | [**10,513**](http://apps.webofknowledge.com.proxy1-bib.sdu.dk:2048/summary.do?product=WOS&doc=1&qid=11&SID=C64CBWXwzZ82V6xabFJ&search_mode=AdvancedSearch&update_back2search_link_param=yes) | ALL=Athymic  *Indexes=SCI-EXPANDED, SSCI, A&HCI, CPCI-S, CPCI-SSH, ESCI Timespan=All years* |
| # 9 | [**3,252**](http://apps.webofknowledge.com.proxy1-bib.sdu.dk:2048/summary.do?product=WOS&doc=1&qid=10&SID=C64CBWXwzZ82V6xabFJ&search_mode=AdvancedSearch&update_back2search_link_param=yes) | ALL=heterotopic transplantation  *Indexes=SCI-EXPANDED, SSCI, A&HCI, CPCI-S, CPCI-SSH, ESCI Timespan=All years* |
| # 8 | [**13,838**](http://apps.webofknowledge.com.proxy1-bib.sdu.dk:2048/summary.do?product=WOS&doc=1&qid=9&SID=C64CBWXwzZ82V6xabFJ&search_mode=AdvancedSearch&update_back2search_link_param=yes) | ALL=heterotopic  *Indexes=SCI-EXPANDED, SSCI, A&HCI, CPCI-S, CPCI-SSH, ESCI Timespan=All years* |
| # 7 | [**17,973**](http://apps.webofknowledge.com.proxy1-bib.sdu.dk:2048/summary.do?product=WOS&doc=1&qid=8&SID=C64CBWXwzZ82V6xabFJ&search_mode=AdvancedSearch&update_back2search_link_param=yes) | ALL=orthotopic transplantation  *Indexes=SCI-EXPANDED, SSCI, A&HCI, CPCI-S, CPCI-SSH, ESCI Timespan=All years* |
| # 6 | [**33,411**](http://apps.webofknowledge.com.proxy1-bib.sdu.dk:2048/summary.do?product=WOS&doc=1&qid=7&SID=C64CBWXwzZ82V6xabFJ&search_mode=AdvancedSearch&update_back2search_link_param=yes) | ALL=orthotopic  *Indexes=SCI-EXPANDED, SSCI, A&HCI, CPCI-S, CPCI-SSH, ESCI Timespan=All years* |
| # 5 | [**274,599**](http://apps.webofknowledge.com.proxy1-bib.sdu.dk:2048/summary.do?product=WOS&doc=1&qid=6&SID=C64CBWXwzZ82V6xabFJ&search_mode=AdvancedSearch&update_back2search_link_param=yes) | ALL=cell transplantation  *Indexes=SCI-EXPANDED, SSCI, A&HCI, CPCI-S, CPCI-SSH, ESCI Timespan=All years* |
| # 4 | [**39,845**](http://apps.webofknowledge.com.proxy1-bib.sdu.dk:2048/summary.do?product=WOS&doc=1&qid=5&SID=C64CBWXwzZ82V6xabFJ&search_mode=AdvancedSearch&update_back2search_link_param=yes) | ALL=tumour xenograft  *Indexes=SCI-EXPANDED, SSCI, A&HCI, CPCI-S, CPCI-SSH, ESCI Timespan=All years* |
| # 3 | [**40,073**](http://apps.webofknowledge.com.proxy1-bib.sdu.dk:2048/summary.do?product=WOS&doc=1&qid=4&SID=C64CBWXwzZ82V6xabFJ&search_mode=AdvancedSearch&update_back2search_link_param=yes) | ALL=tumor xenograft  *Indexes=SCI-EXPANDED, SSCI, A&HCI, CPCI-S, CPCI-SSH, ESCI Timespan=All years* |
| # 2 | [**225,085**](http://apps.webofknowledge.com.proxy1-bib.sdu.dk:2048/summary.do?product=WOS&doc=1&qid=3&SID=C64CBWXwzZ82V6xabFJ&search_mode=AdvancedSearch&update_back2search_link_param=yes) | ALL=Animal experiment  *Indexes=SCI-EXPANDED, SSCI, A&HCI, CPCI-S, CPCI-SSH, ESCI Timespan=All years* |
| # 1 | [**519,836**](http://apps.webofknowledge.com.proxy1-bib.sdu.dk:2048/summary.do?product=WOS&doc=1&qid=2&SID=C64CBWXwzZ82V6xabFJ&search_mode=AdvancedSearch&update_back2search_link_param=yes) | ALL=Animal model  *Indexes=SCI-EXPANDED, SSCI, A&HCI, CPCI-S, CPCI-SSH, ESCI Timespan=All years* |

**Literature search string, Repeated**

**Database:**
 **Embase Classic+Embase <1947 to 2022 August 09>**

| **#** | **Query** | **Results from 10 Aug 2022** |
| --- | --- | --- |
| 1 | exp animal model/ | 1,618,360 |
| 2 | exp animal experiment/ | 2,880,087 |
| 3 | exp tumor xenograft/ | 71,494 |
| 4 | exp cell transplantation/ | 200,766 |
| 5 | exp orthotopic transplantation/ | 2,942 |
| 6 | exp heterotopic transplantation/ | 1,417 |
| 7 | exp genetic engineering/ | 100,382 |
| 8 | exp mouse/ | 2,172,278 |
| 9 | animal model*.mp. | 1,699,886 |
| 10 | animal experiment*.mp. | 2,870,834 |
| 11 | tumor xenograft.mp. | 71,871 |
| 12 | tumour xenograft.mp. | 513 |
| 13 | nf2 model*.mp. | 1 |
| 14 | cell transplantation.mp. | 214,293 |
| 15 | orthotopic transplantation.mp. | 3,803 |
| 16 | heterotopic transplantation.mp. | 1,777 |
| 17 | genetically modified.mp. | 22,828 |
| 18 | genetically engineered.mp. | 20,524 |
| 19 | athymic.mp. | 14,647 |
| 20 | orthotopic.mp. | 48,554 |
| 21 | heterotopic.mp. | 23,989 |
| 22 | transplantation.mp. | 985,340 |
| 23 | exp meningioma/ | 37,663 |
| 24 | mening?oma*.mp. | 43,632 |
| 25 | 1 or 2 or 3 or 4 or 5 or 6 or 7 or 8 or 9 or 10 or 11 or 12 or 13 or 14 or 15 or 16 or 17 or 18 or 19 or 20 or 21 or 22 | 5,162,094 |
| 26 | 23 or 24 | 43,632 |
| 27 | 25 and 26 | 1,249 |
| 28 | limit 27 to yr="2021 -Current" | 159 |

exp animal model/
 exp animal experiment/
 exp tumor xenograft/
 exp cell transplantation/
 exp orthotopic transplantation/
 exp heterotopic transplantation/
 exp genetic engineering/
 exp mouse/
 animal model*.mp.
 animal experiment*.mp.
 tumor xenograft.mp.
 tumour xenograft.mp.
 nf2 model*.mp.
 cell transplantation.mp.
 orthotopic transplantation.mp.
 heterotopic transplantation.mp.
 genetically modified.mp.
 genetically engineered.mp.
 athymic.mp.
 orthotopic.mp.
 heterotopic.mp.
 transplantation.mp.
 exp meningioma/
 mening?oma*.mp.
 1 or 2 or 3 or 4 or 5 or 6 or 7 or 8 or 9 or 10 or 11 or 12 or 13 or 14 or 15 or 16 or 17 or 18 or 19 or 20 or 21 or 22
 23 or 24
 25 and 26
 limit 27 to yr="2021 -Current"


<https://proxy1-bib.sdu.dk:2048/login?url=https://ovidsp.ovid.com/ovidweb.cgi?T=JS&NEWS=N&PAGE=main&SHAREDSEARCHID=7Lp0bxGM8N17HkXJsVM32l7KQNliqWwjdzOkBLbw8xlthbp5JtQlOemmkmYHiC0s8>

**Database:**
 **Ovid MEDLINE(R) ALL <1946 to August 09, 2022>**

| **#** | **Query** | **Results from 10 Aug 2022** |
| --- | --- | --- |
| 1 | exp animal model/ | 631,797 |
| 2 | exp animal experiment/ | 10,212 |
| 3 | exp cell transplantation/ | 111,683 |
| 4 | Animals/ or orthotopic transplantation.mp. or Mice/ | 7,156,691 |
| 5 | orthotopic.mp. | 30,845 |
| 6 | heterotopic transplantation.mp. or exp Transplantation, Heterotopic/ | 3,707 |
| 7 | exp genetic engineering/ | 223,970 |
| 8 | exp mouse/ | 1,739,331 |
| 9 | animal model.mp. | 88,648 |
| 10 | animal experiment.mp. | 3,713 |
| 11 | tumor xenograft.mp. | 4,188 |
| 12 | tumour xenograft.mp. | 361 |
| 13 | nf2 model.mp. | 1 |
| 14 | cell transplantation.mp. | 124,345 |
| 15 | genetically modified.mp. | 80,601 |
| 16 | Animals/ or Disease Models, Animal/ or genetically engineered.mp. or Genetic Engineering/ or Mice/ | 7,177,499 |
| 17 | athymic.mp. | 11,278 |
| 18 | transplantation.mp. | 762,649 |
| 19 | exp meningioma/ | 20,805 |
| 20 | mening?oma*.mp. | 28,441 |
| 21 | 1 or 2 or 3 or 4 or 5 or 6 or 7 or 8 or 9 or 10 or 11 or 12 or 13 or 14 or 15 or 16 or 17 or 18 | 7,865,888 |
| 22 | 19 or 20 | 28,441 |
| 23 | 21 and 22 | 1,324 |
| 24 | limit 23 to yr="2021 -Current" | 63 |

exp animal model/
 exp animal experiment/
 exp cell transplantation/
 Animals/ or orthotopic transplantation.mp. or Mice/
 orthotopic.mp.
 heterotopic transplantation.mp. or exp Transplantation, Heterotopic/
 exp genetic engineering/
 exp mouse/
 animal model.mp.
 animal experiment.mp.
 tumor xenograft.mp.
 tumour xenograft.mp.
 nf2 model.mp.
 cell transplantation.mp.
 genetically modified.mp.
 Animals/ or Disease Models, Animal/ or genetically engineered.mp. or Genetic Engineering/ or Mice/
 athymic.mp.
 transplantation.mp.
 exp meningioma/
 mening?oma*.mp.
 1 or 2 or 3 or 4 or 5 or 6 or 7 or 8 or 9 or 10 or 11 or 12 or 13 or 14 or 15 or 16 or 17 or 18
 19 or 20
 21 and 22
 limit 23 to yr="2021 -Current"


<https://proxy1-bib.sdu.dk:2048/login?url=https://ovidsp.ovid.com/ovidweb.cgi?T=JS&NEWS=N&PAGE=main&SHAREDSEARCHID=3e2gpmJybA4hPkn1qLeqBcPdM8UuVjXHzPf5Pk02qGH11cdcNOOjhBs8fJAWtzQHB>

Web of Science 10-8-22


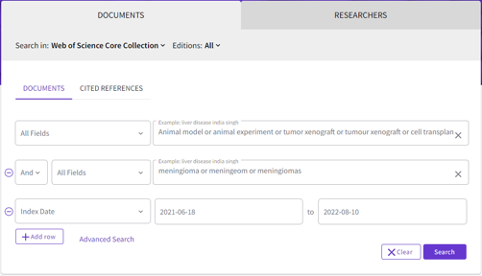

Supplement: Supplementary file 5 — Additional file 5: Full original search strategy. [file 12967_2023_4620_MOESM5_ESM.docx]
