# Supplementary material for: Meningioma animal models: a systematic review and meta-analysis
Source: J Transl Med. 2023 Oct 28;21:764. doi: 10.1186/s12967-023-04620-7 (PMC10612271; doi:10.1186/s12967-023-04620-7)
Supplement: Supplementary file 6 — Additional file 6: Data extraction fields. [file 12967_2023_4620_MOESM6_ESM.docx]

**Supplemental Material 6 – Extraction fields**

**Xenografts using patient-derived established/commercially available cell lines**

| Author | Main author last name (et al. if applicable) |
| --- | --- |
| Year of publication | Year of publication |
| Source of origin/country of origin | Journal paper is published in and description of country or countries of origin(s) – Multiple countries can occur in international collaborations |
| Aims/purposes | Main aim of the paper |
| Established cell type | Type of established cell(s) used. All established cell types injected *in vivo* are to be described in full. |
| Injection volume | Volume injected in microliter |
| Number of cells | Number of cells injected |
| Type of animals | Thorough description of animal type, breed and other traits important to identify animals e.g., manufacturer |
| Sex | Sex of animals |
| Age | Age of animals in weeks |
| Weight | Weight of animals in grams |
| Number | Number of animals |
| Methodology | Orthotopic or heterotopic injection – if described in more detail, this is to be included as well: coordinates, technique etc. |
| Intervention | (if applicable) – e.g., dose, timing of administration, frequency of administration, route of administration, vehicle, scan type |
| Duration of incubation | Duration of incubation in days – If clearly described then extract 50% median survival as well |
| Tumor-take rate | Tumor-take rate in percentages |
| Comparison/verification modalities | Describe which modalities were used to verify growth and to compare xenograft to established cell line e.g., histology, immunohistochemistry, epigenetics/genetics |

**Primary xenografts using patient-derived cells/spheroids/whole tumor pieces**

| Author | Main author last name (et al. if applicable) |
| --- | --- |
| Year of publication | Year of publication |
| Source of origin/country of origin | Journal paper is published in and description of country or countries of origin(s) – Multiple countries can occur in international collaborations |
| Aims/purposes | Main aim of the paper |
| Patient tumor location | Intracranial tumor location |
| WHO grade | Described grade of tumor samples used |
| Injection volume | Volume injected in microliter. (if applicable) |
| Number of cells/spheroids/size tumor | Number of cells injected or size in mm^3^ if whole tumor pieces were used |
| Implanted cell passage | Implanted cell passage (if applicable) |
| Type of animals | Thorough description of animal type, breed and other traits important to identify animals e.g., manufacturer |
| Sex | Sex of animals |
| Age | Age of animals in weeks |
| Weight | Weight of animals in grams |
| Number | Number of animals |
| Methodology | Orthotopic or heterotopic injection – if described in more detail, this is to be included as well: coordinates, technique etc. |
| Intervention | (if applicable) – e.g., dose, timing of administration, frequency of administration, route of administration, vehicle, scan type |
| Duration of incubation | Duration of incubation in days – If clearly described then extract 50% median survival as well |
| Tumor-take rate | Tumor-take rate in percentages |
| Comparison/verification modalities | Describe which modalities were used to verify growth and to compare xenograft to established cell line e.g., histology, immunohistochemistry, epigenetics/genetics |

**Genetically-engineered models**

| Author | Main author last name (et al. if applicable) |
| --- | --- |
| Year of publication | Year of publication |
| Source of origin/country of origin | Journal paper is published in and description of country or countries of origin(s) – Multiple countries can occur in international collaborations |
| Aims/purposes | Main aim of the paper |
| Genetic lesion | Genetic lesion described as detailed as possible |
| Method of gaining lesion | Description of specific method to obtain lesion |
| Type of animals | Thorough description of animal type, breed and other traits important to identify animals e.g., manufacturer |
| Sex | Sex of animals |
| Age | Age of animals in weeks |
| Weight | Weight of animals in grams |
| Number | Number of animals |
| Intervention | (if applicable) – e.g., dose, timing of administration, frequency of administration, route of administration, vehicle, scan type |
| Duration of incubation | Duration of incubation in days – If clearly described then extract 50% median survival as well |
| Tumor-take rate | Tumor-take rate in percentages |
| Tumor-take rate of non-meningiomas | Tumor-take rate in percentages and description of which other tumors were present |
| Comparison/verification modalities | Describe which modalities were used to verify growth and to compare GEM tumor to general terms e.g., histology, immunohistochemistry, epigenetics/genetics |

**Uncategorized models**

| Author | Main author last name (et al. if applicable) |
| --- | --- |
| Year of publication | Year of publication |
| Source of origin/country of origin | Journal paper is published in and description of country or countries of origin(s) – Multiple countries can occur in international collaborations |
| Aims/purposes | Main aim of the paper |
| Method of establishing meningioma growth | Description of specific method used to establish meningioma growth |
| Type of animals | Thorough description of animal type, breed and other traits important to identify animals e.g., manufacturer |
| Sex | Sex of animals |
| Age | Age of animals in weeks |
| Weight | Weight of animals in grams |
| Number | Number of animals |
| Intervention | (if applicable) – e.g., dose, timing of administration, frequency of administration, route of administration, vehicle, scan type |
| Duration of incubation | Duration of incubation in days – If clearly described then extract 50% median survival as well |
| Results | Short description of results |
