# Supplementary material for: Meningioma animal models: a systematic review and meta-analysis
Source: J Transl Med. 2023 Oct 28;21:764. doi: 10.1186/s12967-023-04620-7 (PMC10612271; doi:10.1186/s12967-023-04620-7)
Supplement: Supplementary file 8 — Additional file 8: CRIME-Q: Predetermined list of information. [file 12967_2023_4620_MOESM8_ESM.docx]

Supplemental Material 8 – Predetermined list of information

Example of items/information, which have to be present in each category in order to obtain the various grades: Yes/No/Partly/Unclear/NA

|  | **Category** | **Type** | **Questions and clarification** |
| --- | --- | --- | --- |
| 1X | Peer-review | QoR | X: Peer-reviewed journal  -: Not peer-reviewed i.e., preprint: |
| 2X | Bench-top/laboratory work related to establishing model – reporting | QoR | X: Full description of origin of cells, incubator settings, cell medium including addons (FBS%, P/S etc.), cell handling described  (X): Lack of some information i.e., incubator settings, but still somewhat replicable.  -: Poor description, not replicable or transparent |
| 2Y | Bench-top/laboratory work related to establishing model – methodology | MQ | Based on standard practice and majority of successful *in vitro*  X: Incubator 37 degrees Celsius and 5% CO2, Use of DMEM, 7,5-15% FBS and P/S 1%, proper handling of cells (medium changes)  (X): Few items not performed properly or problematic quality of reporting can decrease value.  -: i.e. high CO2, not appropriate medium (i.e., neurobasal for meningioma cells), other handling which could affect results.  Studies with poor reporting (2X) will have difficulty gaining high 2Y because of low transparency and ability to assess method quality |
| 3X | Animals – Reporting | QoR | X: Full description of all the parameters: Type, breed, age, weight, manufacturer. If type, age, and weight were sufficiently described OR if weight was missing, did they include manufacturer – if yes then full X.  (X): Some lacking information, but not entirely.  -: If animals used cannot be identified properly. Complete or near complete lack of information |
| 3Y | Animals – Methodology | MQ | X: Study used similar baseline characteristics for the animals (age, weight, type). i.e., Narrow age gaps Age  (X): I.e., large age gap e.g., 4-15 weeks. Incongruency in weight, which were not addressed as to why.  -: Innapropriate type of animals used for the experiments. Incongruency in weight between intervention groups or very large incongruency in observational studies (varying weight, age and even different types of animals used)  Studies with poor reporting (3X) will have difficulty gaining high 3Y because of low transparency and ability to assess method quality |
| 3Z | Selection bias (baseline characteristics) **(SYRCLE Item 2)** | RoB | See SYRCLE item 2 for more elaborate description and signal questions:  Was the distribution of relevant baseline characteristics balanced between groups? I.e., was the distribution of i.e., male:female ratios, species, strain, age and weight equally distributed throughout groups? **Yes/No/Unclear/NA**. Not applicable for studies using only one group. |
| 4X | Sample size calculation | QoR | X: Sample size calculation performed and shown  (X): Description of sample size calculation and possibly power, but no certain number given.  -: Not performed and not described |
| 5X | *in vivo* design and performance – Reporting | QoR | X: Description of number of animals, full description of design (duration of experiment, intervention (if applicable), procedure to produce animal model, i.e. surgical procedure in reproducible details, how genetically engineered model was performed etc. Randomization if applicable. How outcome measures were preformed, i.e., how to assess difference in size of tumors.  (X): Some information lacking for complete transparency but can still be performed in some capacity.  -: Poorly described in such a degree experiment cannot be replicated. |
| 5Y | *in vivo* design and performance -Methodology | MQ | Y: If the method seemed feasible and well performed in relation to the study’s aim and outcome and in contrast to other known literature. i.e. cell injection performed slowly, proper coordinates (i.e., lateral, frontal of bregma), intervention feasible in relation to i.e., drug dose. (Were items from 5X properly performed and feasible)  (Y): Aspects not well performed, but not in a degree poor methodology can be assessed, i.e., few animals in groups 1-3, free hand cell injection in a fixed area using coordinates, which would otherwise require stereotactic frame.  -: Cannot be assessed or poorly performed i.e., outcome cannot be achieved using the method described. Studies with poor reporting (5X) will have difficulty gaining high 5Y because of low transparency and ability to assess method quality |
| 5Z (1) | Selection bias (Sequence generation) **(SYRCLE Item 1)** | RoB | See SYRCLE item 1 for more elaborate description and signal questions:  Was there a description of allocation (the process by which experimental units are assigned to experimental groups). Yes/no/unclear. Not applicable for non-intervention studies.  *Item is made more lenient to now include a YES in all cases of described randomization.* **Yes/No/Unclear/NA** |
| 5Z (2) | Performance bias (Random housing) **(SYRCLE Item 4)** | RoB | See SYRCLE item 4 for more elaborate description and signal questions:  Were the animals randomly housed during the experiment? Yes/no/unclear. Not applicable for non-intervention studies. **Yes/No/Unclear/NA** |
| 5Z (3) | Detection bias (Random outcome assessment) **(SYRCLE Item 6)** | RoB | See SYRCLE item 6 for more elaborate description and signal questions:  Were animals randomly selected for outcome. For instance, if human endpoints (i.e., poor conditions, weight etc.) were met and the investigators were not blinded, then outcome cannot be assessed randomly. Not applicable for non-intervention studies. **Yes/No/Unclear/NA** |
| 6X | Compliance with animal welfare regulations | QoR | X: Study describes compliance with a specific animal welfare regulation.  -: No description of compliance with animal welfare regulations |
| 7X | Blinding | QoR | X: Described blinding of any type (subtype of blinding described in 7Z (1-3))  -: No blinding described |
| 7Z (1) | Performance bias (Blinding) **(SYRCLE Item 5)** | RoB | See SYRCLE item 5 for more elaborate description and signal questions:  Describe all used, if any, to blind trial caregiver and researchers from knowing which intervention each animal received. Not applicable for non-intervention studies, however could be applicable for instance in xenograft studies, where multiple patient samples where used. **Yes/No/Unclear/NA** |
| 7Z (2) | Allocation bias (allocation concealment) **(SYRCLE Item 3)** | RoB | See SYRCLE item 3 for more elaborate description and signal questions:  Could the investigator allocating the animals to intervention or control group not foresee assignment. **Yes/No/Unclear/NA.** Not applicable for non-intervention studies. Yes/no/unclear/NA. Could be applicable for instance in xenograft studies, where multiple patient samples where used. |
| 7Z (3) | Detection bias (blinding) **(SYRCLE Item 7)** | RoB | See SYRCLE item 7 for more elaborate description and signal questions:  Was the outcome assessor blinded? and could the blinding have been broken? Describe all measure used, if any, to blind outcome assessors from knowing which intervention each animal received. The outcome assessment methods were the same in both groups?  **Yes/No/Unclear/NA**. Could be applicable for instance in xenograft studies, where multiple patient samples where used. |
| 8X | Congruency between methods and results | QoR | X: Congruency between methods described and result section  (X): Less important methods not described, i.e., how to perform HE sections.  -: Important details and information not described in either result or method section |
| 8Z (1) | Attrition bias (incomplete outcome data) **(SYRCLE Item 8)** | RoB | See SYRCLE item 8 for more elaborate description and signal questions:  Describe completeness of outcome data including attrition and exclusions from the analysis and were incomplete outcome data adequately described? Were all animals included in the analysis and if no, was it described why they were not included? **Yes/No/Unclear** |
| 8Z (2) | Reporting bias (Selective outcome reporting) **(SYRCLE Item 9) Yes/No/Unclear** | RoB | See SYRCLE item 9 for more elaborate description and signal questions:  Was the study protocol available (require description of protocol location in paper) and were all of the study’s pre-specified primary and secondary outcomes reported in the manuscript. Was the study protocol not available but was it clear that the published report included all expected outcomes (i.e., comparing methods and result section).The study report fails to include results for a key outcome that would be expected to haven been reported for such a study, i.e. tumor-take rate in transplantation experiments. **Yes/No/Unclear** |
| 9X | Presentation of limitations | QoR | X: Separate limitations section or thoroughly described limitations in discussion  (X): Some comment but not direct limitations  -: No limitations described  All in relation to *in vivo and/or in vitro* subparts |
| 10X | Statement of potential conflict of interest | QoR | X: Statement of potential conflict of interest  (X): Disclaimers, but no statement of potential conflict of interest  -: No statement of potential conflict of interest |
| 10Z | Other bias (Publication bias) (**SYRCLE Item 10)** | RoB | See SYRCLE item 10 for more elaborate description and signal questions – This item is specifically narrowed to the following:  Inappropriate influence of funders or biased by companies. Was the study free of inappropriate influence of funders or companies supplying drugs or equipment? Did the authors declare direct conflict of interest in relation to study? (Lastly if yes then no). Yes: Conflict of interest statement with no conflict of interests. **Yes/No/Unclear** |
