# Supplementary material for: Meningioma animal models: a systematic review and meta-analysis
Source: J Transl Med. 2023 Oct 28;21:764. doi: 10.1186/s12967-023-04620-7 (PMC10612271; doi:10.1186/s12967-023-04620-7)
Supplement: Supplementary file 13 — Additional file 13: Meta- and subgroup analyses. [file 12967_2023_4620_MOESM13_ESM.pdf]

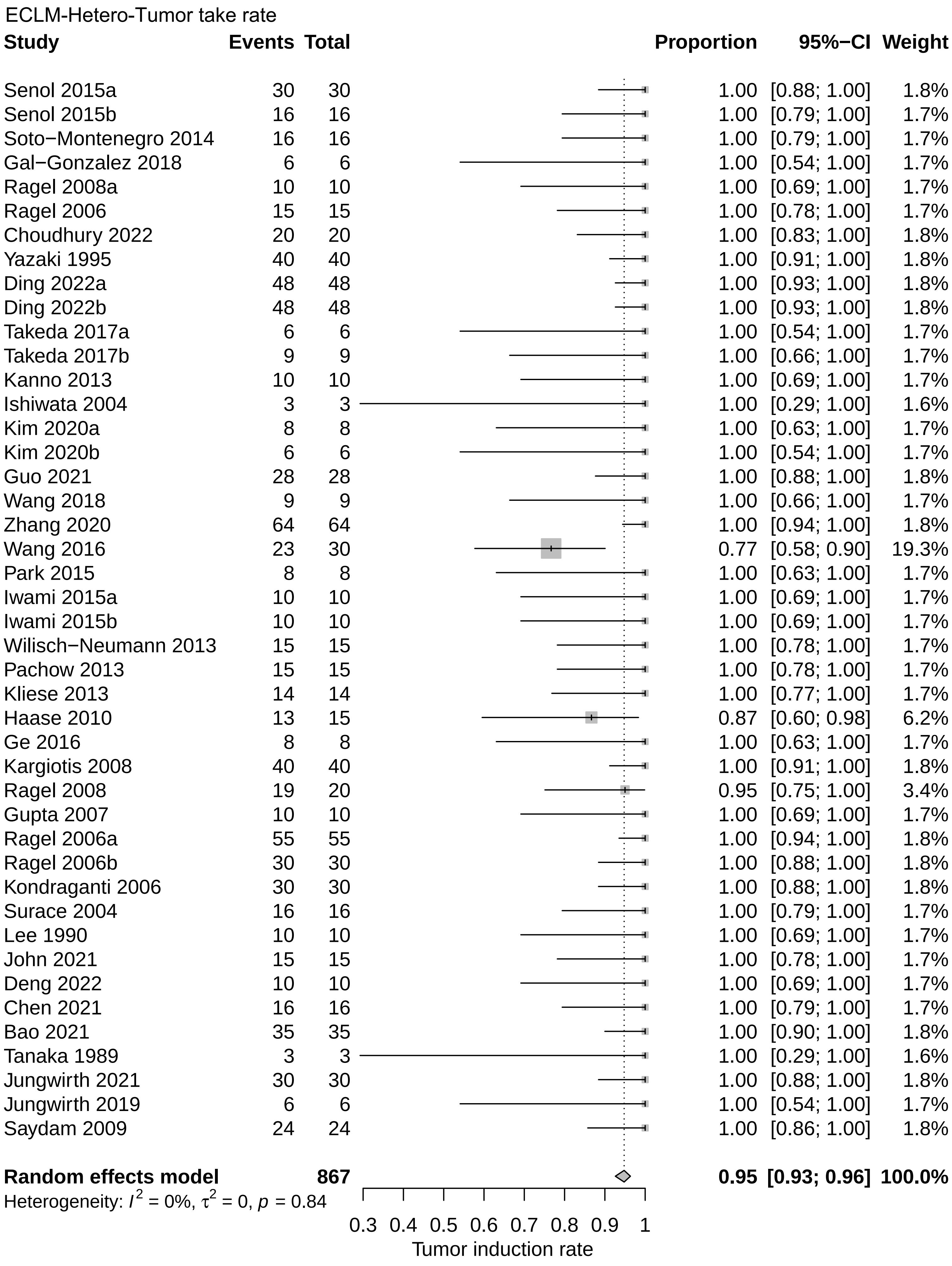
ECLM-Hetero-Tumor take rate
Study Events Total Proportion 95%−CI Weight
Senol 2015a 30 30 1.00 [0.88; 1.00] 1.8%
Senol 2015b 16 16 1.00 [0.79; 1.00] 1.7%
Soto−Montenegro 2014 16 16 1.00 [0.79; 1.00] 1.7%
Gal−Gonzalez 2018 6 6 1.00 [0.54; 1.00] 1.7%
Ragel 2008a 10 10 1.00 [0.69; 1.00] 1.7%
Ragel 2006 15 15 1.00 [0.78; 1.00] 1.7%
Choudhury 2022 20 20 1.00 [0.83; 1.00] 1.8%
Yazaki 1995 40 40 1.00 [0.91; 1.00] 1.8%
Ding 2022a 48 48 1.00 [0.93; 1.00] 1.8%
Ding 2022b 48 48 1.00 [0.93; 1.00] 1.8%
Takeda 2017a 6 6 1.00 [0.54; 1.00] 1.7%
Takeda 2017b 9 9 1.00 [0.66; 1.00] 1.7%
Kanno 2013 10 10 1.00 [0.69; 1.00] 1.7%
Ishiwata 2004 3 3 1.00 [0.29; 1.00] 1.6%
Kim 2020a 8 8 1.00 [0.63; 1.00] 1.7%
Kim 2020b 6 6 1.00 [0.54; 1.00] 1.7%
Guo 2021 28 28 1.00 [0.88; 1.00] 1.8%
Wang 2018 9 9 1.00 [0.66; 1.00] 1.7%
Zhang 2020 64 64 1.00 [0.94; 1.00] 1.8%
Wang 2016 23 30 0.77 [0.58; 0.90] 19.3%
Park 2015 8 8 1.00 [0.63; 1.00] 1.7%
Iwami 2015a 10 10 1.00 [0.69; 1.00] 1.7%
Iwami 2015b 10 10 1.00 [0.69; 1.00] 1.7%
Wilisch−Neumann 2013 15 15 1.00 [0.78; 1.00] 1.7%
Pachow 2013 15 15 1.00 [0.78; 1.00] 1.7%
Kliese 2013 14 14 1.00 [0.77; 1.00] 1.7%
Haase 2010 13 15 0.87 [0.60; 0.98] 6.2%
Ge 2016 8 8 1.00 [0.63; 1.00] 1.7%
Kargiotis 2008 40 40 1.00 [0.91; 1.00] 1.8%
Ragel 2008 19 20 0.95 [0.75; 1.00] 3.4%
Gupta 2007 10 10 1.00 [0.69; 1.00] 1.7%
Ragel 2006a 55 55 1.00 [0.94; 1.00] 1.8%
Ragel 2006b 30 30 1.00 [0.88; 1.00] 1.8%
Kondraganti 2006 30 30 1.00 [0.88; 1.00] 1.8%
Surace 2004 16 16 1.00 [0.79; 1.00] 1.7%
Lee 1990 10 10 1.00 [0.69; 1.00] 1.7%
John 2021 15 15 1.00 [0.78; 1.00] 1.7%
Deng 2022 10 10 1.00 [0.69; 1.00] 1.7%
Chen 2021 16 16 1.00 [0.79; 1.00] 1.7%
Bao 2021 35 35 1.00 [0.90; 1.00] 1.8%
Tanaka 1989 3 3 1.00 [0.29; 1.00] 1.6%
Jungwirth 2021 30 30 1.00 [0.88; 1.00] 1.8%
Jungwirth 2019 6 6 1.00 [0.54; 1.00] 1.7%
Saydam 2009 24 24 1.00 [0.86; 1.00] 1.8%
Random effects model 867 0.95 [0.93; 0.96] 100.0%
Heterogeneity: I^2 = 0%, τ^2 = 0, p = 0.84
0.3 0.4 0.5 0.6 0.7 0.8 0.9 1
Tumor induction rate

ECLM-Ortho-Tumor take rate

Study	Events	Total	Proportion	95%−CI	Weight
McCutcheon 2000a	15	15	1.00	[0.78; 1.00]	1.7%
McCutcheon 2000b	15	15	1.00	[0.78; 1.00]	1.7%
Püttmann 2005	5	5	1.00	[0.48; 1.00]	1.6%
Chang 2021	40	40	1.00	[0.91; 1.00]	1.7%
Chen 2021	12	12	1.00	[0.74; 1.00]	1.6%
Karsy 2016	54	54	1.00	[0.93; 1.00]	1.7%
Ragel 2008a	40	45	0.89	[0.76; 0.96]	15.2%
Ragel 2008b	9	10	0.90	[0.55; 1.00]	3.1%
La Cava 2018a	22	24	0.92	[0.73; 0.99]	6.3%
La Cava 2018b	18	23	0.78	[0.56; 0.93]	13.3%
Yazaki 1995	15	16	0.94	[0.70; 1.00]	3.2%
van Furth 2003	15	15	1.00	[0.78; 1.00]	1.7%
Nakano 2022	24	24	1.00	[0.86; 1.00]	1.7%
Bao 2021	35	35	1.00	[0.90; 1.00]	1.7%
Ho 2018	40	40	1.00	[0.91; 1.00]	1.7%
Tuchen 2016	35	35	1.00	[0.90; 1.00]	1.7%
Iwami 2015	5	5	1.00	[0.48; 1.00]	1.6%
Wilisch−Neumann 2013	30	30	1.00	[0.88; 1.00]	1.7%
Iwami 2013	24	24	1.00	[0.86; 1.00]	1.7%
Pachow 2013	16	16	1.00	[0.79; 1.00]	1.7%
Kliese 2013	8	8	1.00	[0.63; 1.00]	1.6%
Ragel 2008c	15	15	1.00	[0.78; 1.00]	1.7%
Baia 2007a	5	5	1.00	[0.48; 1.00]	1.6%
Baia 2007b	5	5	1.00	[0.48; 1.00]	1.6%
Baia 2007c	4	4	1.00	[0.40; 1.00]	1.5%
Baia 2007d	5	5	1.00	[0.48; 1.00]	1.6%
Salhia 2002	75	75	1.00	[0.95; 1.00]	1.7%
Gogineni 2010	20	20	1.00	[0.83; 1.00]	1.7%
Waldt 2021	12	12	1.00	[0.74; 1.00]	1.6%
Tummalapalli 2007	30	30	1.00	[0.88; 1.00]	1.7%
van Furth 2003	15	15	1.00	[0.78; 1.00]	1.7%
Nalla 2011	20	20	1.00	[0.83; 1.00]	1.7%
Nakano 2021	24	24	1.00	[0.86; 1.00]	1.7%
Wilisch−Neumann 2013	10	10	1.00	[0.69; 1.00]	1.6%
Pachow 2013	10	10	1.00	[0.69; 1.00]	1.6%
P2011	10	10	1.00	[0.69; 1.00]	1.6%
Cargioli 2007a	6	6	1.00	[0.54; 1.00]	1.6%
Cargioli 2007b	6	6	1.00	[0.54; 1.00]	1.6%
Nigim 2019a	14	14	1.00	[0.77; 1.00]	1.6%
Nigim 2016	12	12	1.00	[0.74; 1.00]	1.6%
Tummalapalli 2007	30	30	1.00	[0.88; 1.00]	1.7%
**Random effects model**		**819**	**0.94**	**[0.92; 0.96]**	**100.0%**

Heterogeneity: $I^2 = 0\%$, $\tau^2 = 0$, $p = 0.92$

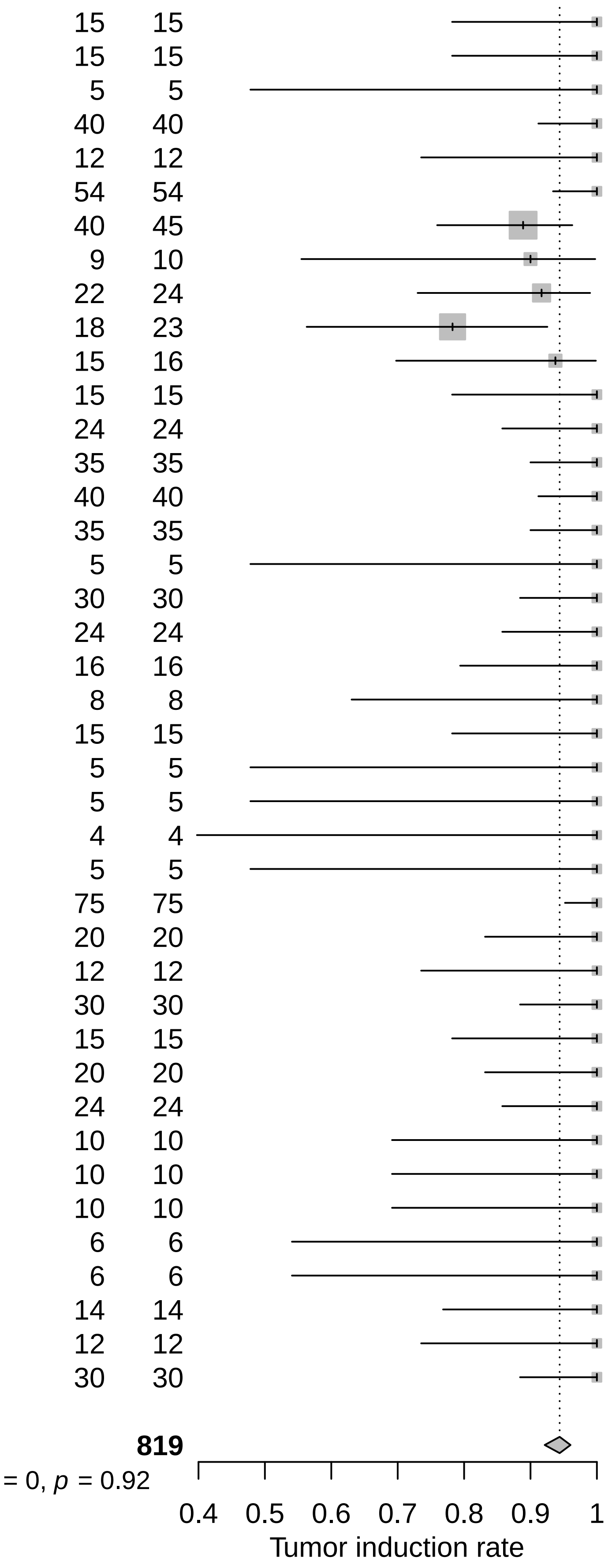

PTM-Hetero-Tumor take rate

Study	Events	Total	Proportion	95%−CI	Weight
Jiang 2017a	4	4	1.00	[0.40; 1.00]	2.2%
Jiang 2017b	4	4	1.00	[0.40; 1.00]	2.2%
Jiang 2017c	4	4	1.00	[0.40; 1.00]	2.2%
Jiang 2017d	4	4	1.00	[0.40; 1.00]	2.2%
Jiang 2017e	4	4	1.00	[0.40; 1.00]	2.2%
Jiang 2017f	4	4	1.00	[0.40; 1.00]	2.2%
Jiang 2017g	4	4	1.00	[0.40; 1.00]	2.2%
Ding 2015	6	6	1.00	[0.54; 1.00]	2.2%
Ragel 2008a	5	5	1.00	[0.48; 1.00]	2.2%
Ragel 2008b	5	5	1.00	[0.48; 1.00]	2.2%
Ragel 2008c	0	5	0.00	[0.00; 0.52]	2.2%
Ragel 2008d	5	5	1.00	[0.48; 1.00]	2.2%
Chen 2007	17	20	0.85	[0.62; 0.97]	5.3%
Ragel 2006a	10	10	1.00	[0.69; 1.00]	2.2%
Hu 2012a	3	6	0.50	[0.12; 0.88]	4.3%
Hu 2012b	3	5	0.60	[0.15; 0.95]	3.9%
Hu 2012c	4	6	0.67	[0.22; 0.96]	4.1%
Malham 2001a	2	4	0.50	[0.07; 0.93]	3.6%
Malham 2001b	3	3	1.00	[0.29; 1.00]	2.1%
Malham 2001c	4	6	0.67	[0.22; 0.96]	4.1%
Malham 2001d	5	5	1.00	[0.48; 1.00]	2.2%
Malham 2001e	4	6	0.67	[0.22; 0.96]	4.1%
Malham 2001f	0	6	0.00	[0.00; 0.46]	2.2%
Malham 2001g	7	10	0.70	[0.35; 0.93]	5.0%
Malham 2001h	14	20	0.70	[0.46; 0.88]	6.0%
Jensen 2001	76	76	1.00	[0.95; 1.00]	2.3%
McCutcheon 2001a	20	20	1.00	[0.83; 1.00]	2.3%
McCutcheon 2001b	8	8	1.00	[0.63; 1.00]	2.2%
McCutcheon 2001c	2	2	1.00	[0.16; 1.00]	2.0%
Schrell 1997	50	50	1.00	[0.93; 1.00]	2.3%
Yazaki 1995a	36	36	1.00	[0.90; 1.00]	2.3%
Yazaki 1995b	20	20	1.00	[0.83; 1.00]	2.3%
Matsuda 1994a	19	19	1.00	[0.82; 1.00]	2.3%
Matsuda 1994b	12	12	1.00	[0.74; 1.00]	2.3%
Matsuda 1994c	20	20	1.00	[0.83; 1.00]	2.3%
Olson 1987	6	6	1.00	[0.54; 1.00]	2.2%
**Random effects model**		**430**	**0.85**	**[0.78; 0.91]**	**100.0%**

Heterogeneity: $I^2 = 41\%$, $\tau^2 = 0.8685$, $p < 0.01$

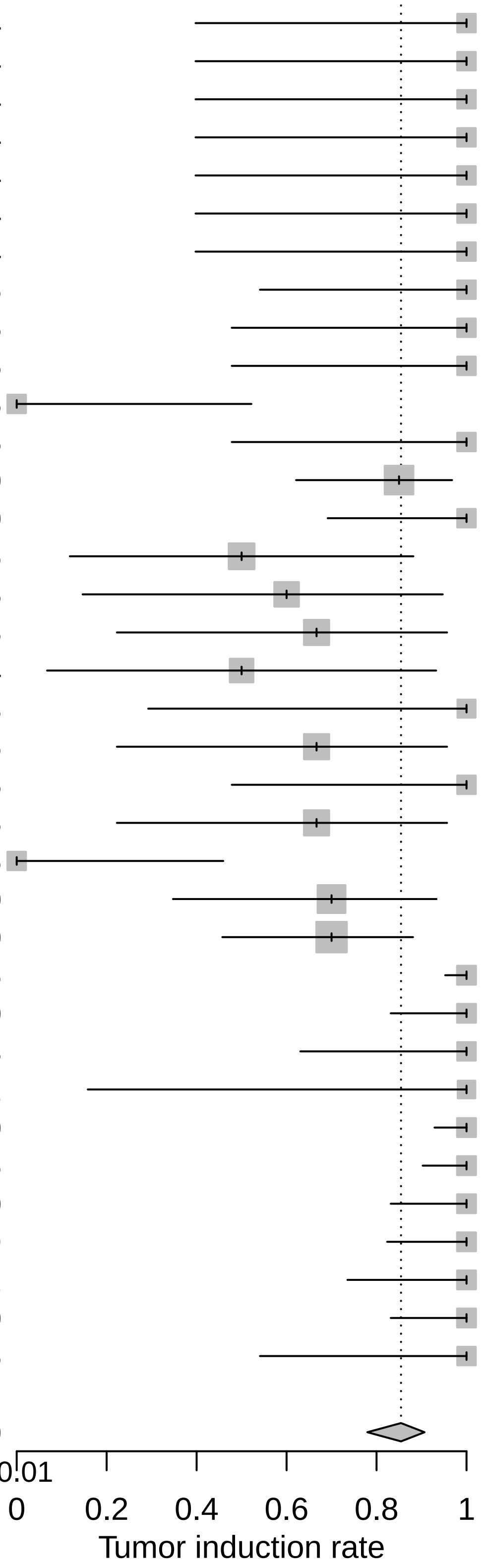

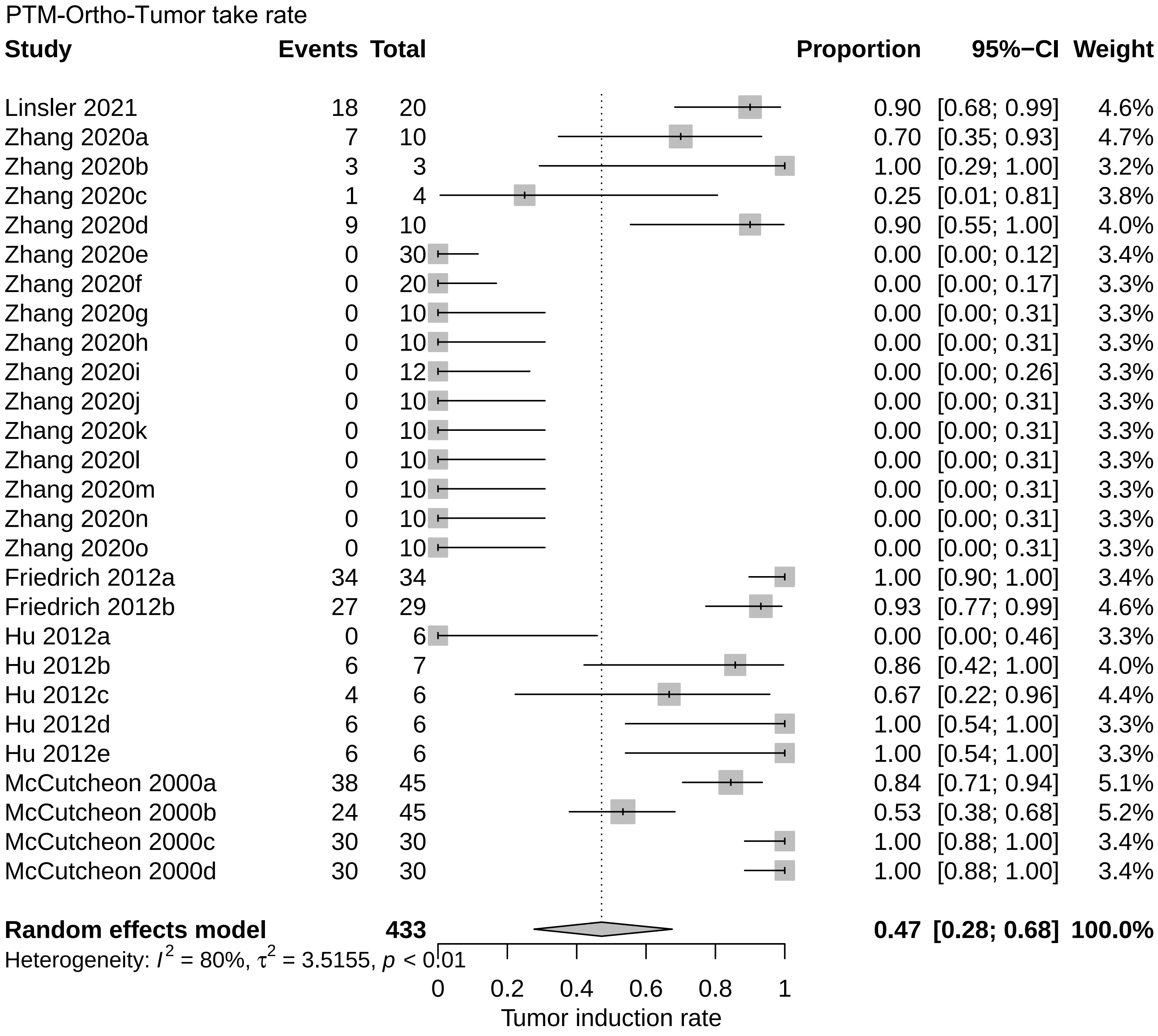
PTM-Ortho-Tumor take rate
Study Events Total Proportion 95%−CI Weight
Linsler 2021 18 20 0.90 [0.68; 0.99] 4.6%
Zhang 2020a 7 10 0.70 [0.35; 0.93] 4.7%
Zhang 2020b 3 3 1.00 [0.29; 1.00] 3.2%
Zhang 2020c 1 4 0.25 [0.01; 0.81] 3.8%
Zhang 2020d 9 10 0.90 [0.55; 1.00] 4.0%
Zhang 2020e 0 30 0.00 [0.00; 0.12] 3.4%
Zhang 2020f 0 20 0.00 [0.00; 0.17] 3.3%
Zhang 2020g 0 10 0.00 [0.00; 0.31] 3.3%
Zhang 2020h 0 10 0.00 [0.00; 0.31] 3.3%
Zhang 2020i 0 12 0.00 [0.00; 0.26] 3.3%
Zhang 2020j 0 10 0.00 [0.00; 0.31] 3.3%
Zhang 2020k 0 10 0.00 [0.00; 0.31] 3.3%
Zhang 2020l 0 10 0.00 [0.00; 0.31] 3.3%
Zhang 2020m 0 10 0.00 [0.00; 0.31] 3.3%
Zhang 2020n 0 10 0.00 [0.00; 0.31] 3.3%
Zhang 2020o 0 10 0.00 [0.00; 0.31] 3.3%
Friedrich 2012a 34 34 1.00 [0.90; 1.00] 3.4%
Friedrich 2012b 27 29 0.93 [0.77; 0.99] 4.6%
Hu 2012a 0 6 0.00 [0.00; 0.46] 3.3%
Hu 2012b 6 7 0.86 [0.42; 1.00] 4.0%
Hu 2012c 4 6 0.67 [0.22; 0.96] 4.4%
Hu 2012d 6 6 1.00 [0.54; 1.00] 3.3%
Hu 2012e 6 6 1.00 [0.54; 1.00] 3.3%
McCutcheon 2000a 38 45 0.84 [0.71; 0.94] 5.1%
McCutcheon 2000b 24 45 0.53 [0.38; 0.68] 5.2%
McCutcheon 2000c 30 30 1.00 [0.88; 1.00] 3.4%
McCutcheon 2000d 30 30 1.00 [0.88; 1.00] 3.4%
Random effects model 433 0.47 [0.28; 0.68] 100.0%
Heterogeneity: I^2 = 80%, τ^2 = 3.5155, p < 0.01
0 0.2 0.4 0.6 0.8 1
Tumor induction rate

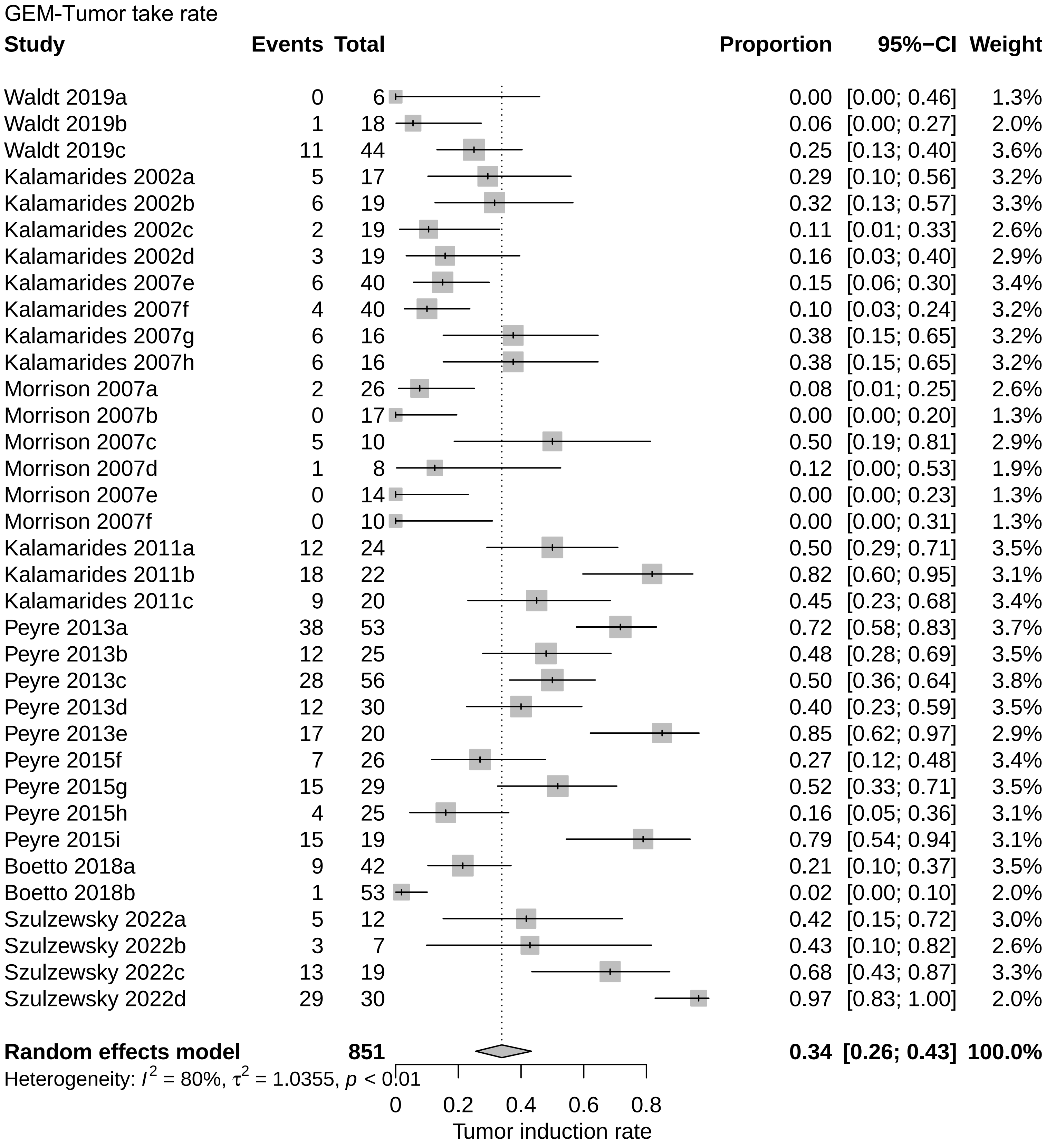
GEM-Tumor take rate
Study Events Total Proportion 95%−CI Weight
Waldt 2019a 0 6 0.00 [0.00; 0.46] 1.3%
Waldt 2019b 1 18 0.06 [0.00; 0.27] 2.0%
Waldt 2019c 11 44 0.25 [0.13; 0.40] 3.6%
Kalamarides 2002a 5 17 0.29 [0.10; 0.56] 3.2%
Kalamarides 2002b 6 19 0.32 [0.13; 0.57] 3.3%
Kalamarides 2002c 2 19 0.11 [0.01; 0.33] 2.6%
Kalamarides 2002d 3 19 0.16 [0.03; 0.40] 2.9%
Kalamarides 2007e 6 40 0.15 [0.06; 0.30] 3.4%
Kalamarides 2007f 4 40 0.10 [0.03; 0.24] 3.2%
Kalamarides 2007g 6 16 0.38 [0.15; 0.65] 3.2%
Kalamarides 2007h 6 16 0.38 [0.15; 0.65] 3.2%
Morrison 2007a 2 26 0.08 [0.01; 0.25] 2.6%
Morrison 2007b 0 17 0.00 [0.00; 0.20] 1.3%
Morrison 2007c 5 10 0.50 [0.19; 0.81] 2.9%
Morrison 2007d 1 8 0.12 [0.00; 0.53] 1.9%
Morrison 2007e 0 14 0.00 [0.00; 0.23] 1.3%
Morrison 2007f 0 10 0.00 [0.00; 0.31] 1.3%
Kalamarides 2011a 12 24 0.50 [0.29; 0.71] 3.5%
Kalamarides 2011b 18 22 0.82 [0.60; 0.95] 3.1%
Kalamarides 2011c 9 20 0.45 [0.23; 0.68] 3.4%
Peyre 2013a 38 53 0.72 [0.58; 0.83] 3.7%
Peyre 2013b 12 25 0.48 [0.28; 0.69] 3.5%
Peyre 2013c 28 56 0.50 [0.36; 0.64] 3.8%
Peyre 2013d 12 30 0.40 [0.23; 0.59] 3.5%
Peyre 2013e 17 20 0.85 [0.62; 0.97] 2.9%
Peyre 2015f 7 26 0.27 [0.12; 0.48] 3.4%
Peyre 2015g 15 29 0.52 [0.33; 0.71] 3.5%
Peyre 2015h 4 25 0.16 [0.05; 0.36] 3.1%
Peyre 2015i 15 19 0.79 [0.54; 0.94] 3.1%
Boetto 2018a 9 42 0.21 [0.10; 0.37] 3.5%
Boetto 2018b 1 53 0.02 [0.00; 0.10] 2.0%
Szulzewsky 2022a 5 12 0.42 [0.15; 0.72] 3.0%
Szulzewsky 2022b 3 7 0.43 [0.10; 0.82] 2.6%
Szulzewsky 2022c 13 19 0.68 [0.43; 0.87] 3.3%
Szulzewsky 2022d 29 30 0.97 [0.83; 1.00] 2.0%
Random effects model 851 0.34 [0.26; 0.43] 100.0%
Heterogeneity: I^2 = 80%, τ^2 = 1.0355, p < 0.01
0 0.2 0.4 0.6 0.8
Tumor induction rate

ECLM-Hetero-Duration of incubation

Study	Events	Total	Proportion	95%−CI	Weight
**incubation_category = 0−14 days**					
Senol 2015a	30	30	1.00	[0.88; 1.00]	1.8%
Senol 2015b	16	16	1.00	[0.79; 1.00]	1.7%
Zhang 2020	64	64	1.00	[0.94; 1.00]	1.8%
Iwami 2015a	10	10	1.00	[0.69; 1.00]	1.7%
Iwami 2015b	10	10	1.00	[0.69; 1.00]	1.7%
Kliese 2013	14	14	1.00	[0.77; 1.00]	1.7%
Surace 2004	16	16	1.00	[0.79; 1.00]	1.7%
**Random effects model**		**160**	**0.97**	**[0.93; 0.99]**	**12.2%**
Heterogeneity: $I^2 = 0\%$, $\tau^2 = 0$, $p = 0.97$					
**incubation_category = 14−30 days**					
Soto−Montenegro 2014	16	16	1.00	[0.79; 1.00]	1.7%
Choudhury 2022	20	20	1.00	[0.83; 1.00]	1.8%
Yazaki 1995	40	40	1.00	[0.91; 1.00]	1.8%
Kim 2020a	8	8	1.00	[0.63; 1.00]	1.7%
Kim 2020b	6	6	1.00	[0.54; 1.00]	1.7%
Guo 2021	28	28	1.00	[0.88; 1.00]	1.8%
Wang 2016	23	30	0.77	[0.58; 0.90]	19.3%
Park 2015	8	8	1.00	[0.63; 1.00]	1.7%
Wilisch−Neumann 2013	15	15	1.00	[0.78; 1.00]	1.7%
Pachow 2013	15	15	1.00	[0.78; 1.00]	1.7%
Haase 2010	13	15	0.87	[0.60; 0.98]	6.2%
Ge 2016	8	8	1.00	[0.63; 1.00]	1.7%
Kargiotis 2008	40	40	1.00	[0.91; 1.00]	1.8%
Gupta 2007	10	10	1.00	[0.69; 1.00]	1.7%
Kondraganti 2006	30	30	1.00	[0.88; 1.00]	1.8%
John 2021	15	15	1.00	[0.78; 1.00]	1.7%
Deng 2022	10	10	1.00	[0.69; 1.00]	1.7%
Jungwirth 2021	30	30	1.00	[0.88; 1.00]	1.8%
Saydam 2009	24	24	1.00	[0.86; 1.00]	1.8%
**Random effects model**		**368**	**0.94**	**[0.90; 0.97]**	**55.2%**
Heterogeneity: $I^2 = 14\%$, $\tau^2 = 0.2330$, $p = 0.28$					
**incubation_category = Unknown**					
Gal−Gonzalez 2018	6	6	1.00	[0.54; 1.00]	1.7%
Ding 2022b	48	48	1.00	[0.93; 1.00]	1.8%
Takeda 2017b	9	9	1.00	[0.66; 1.00]	1.7%
Lee 1990	10	10	1.00	[0.69; 1.00]	1.7%
**Random effects model**		**73**	**0.96**	**[0.87; 0.99]**	**6.9%**
Heterogeneity: $I^2 = 0\%$, $\tau^2 = 0$, $p = 0.77$					
**incubation_category = 31−99 days**					
Ragel 2008a	10	10	1.00	[0.69; 1.00]	1.7%
Ragel 2006	15	15	1.00	[0.78; 1.00]	1.7%
Ding 2022a	48	48	1.00	[0.93; 1.00]	1.8%
Kanno 2013	10	10	1.00	[0.69; 1.00]	1.7%
Ishiwata 2004	3	3	1.00	[0.29; 1.00]	1.6%
Wang 2018	9	9	1.00	[0.66; 1.00]	1.7%
Ragel 2008	19	20	0.95	[0.75; 1.00]	3.4%
Ragel 2006a	55	55	1.00	[0.94; 1.00]	1.8%
Ragel 2006b	30	30	1.00	[0.88; 1.00]	1.8%
Chen 2021	16	16	1.00	[0.79; 1.00]	1.7%
Bao 2021	35	35	1.00	[0.90; 1.00]	1.8%
Tanaka 1989	3	3	1.00	[0.29; 1.00]	1.6%
Jungwirth 2019	6	6	1.00	[0.54; 1.00]	1.7%
**Random effects model**		**260**	**0.96**	**[0.93; 0.98]**	**24.0%**
Heterogeneity: $I^2 = 0\%$, $\tau^2 = 0$, $p = 0.97$					
**incubation_category = 100−199 days**					
Takeda 2017a	6	6	1.00	[0.54; 1.00]	1.7%
**Random effects model**		**867**	**0.95**	**[0.93; 0.96]**	**100.0%**

Heterogeneity: $I^2 = 0\%$, $\tau^2 = 0$, $p = 0.84$

Test for subgroup differences: $\chi^2_4 = 2.31$, df = 4 ($p = 0.68$)

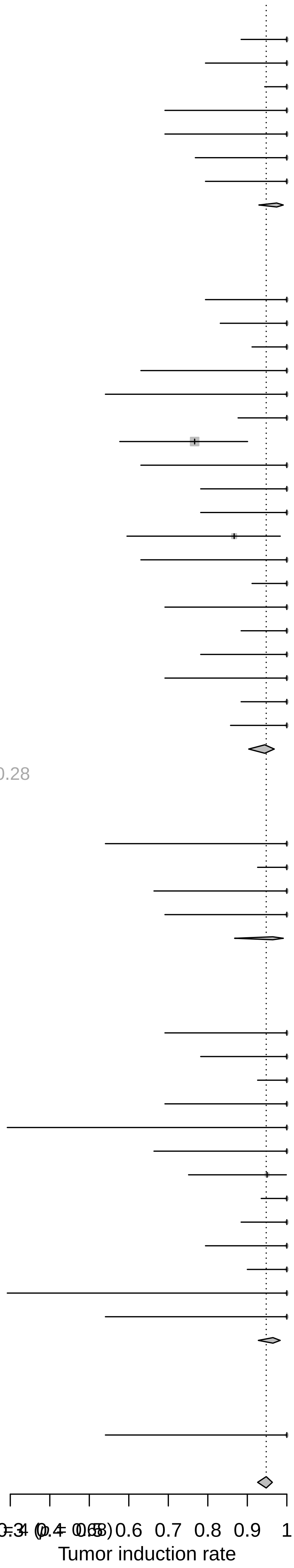

ECLM-Ortho-Duration of incubation

Study	Events	Total	Proportion	95%−CI	Weight
**incubation_category = Unknown**					
McCutcheon 2000a	15	15	1.00	[0.78; 1.00]	1.7%
McCutcheon 2000b	15	15	1.00	[0.78; 1.00]	1.7%
Ragel 2008a	40	45	0.89	[0.76; 0.96]	15.2%
Ragel 2008b	9	10	0.90	[0.55; 1.00]	3.1%
Yazaki 1995	15	16	0.94	[0.70; 1.00]	3.2%
Nakano 2022	24	24	1.00	[0.86; 1.00]	1.7%
Bao 2021	35	35	1.00	[0.90; 1.00]	1.7%
Ho 2018	40	40	1.00	[0.91; 1.00]	1.7%
Baia 2007a	5	5	1.00	[0.48; 1.00]	1.6%
Baia 2007b	5	5	1.00	[0.48; 1.00]	1.6%
Baia 2007c	4	4	1.00	[0.40; 1.00]	1.5%
Baia 2007d	5	5	1.00	[0.48; 1.00]	1.6%
Salhia 2002	75	75	1.00	[0.95; 1.00]	1.7%
Waldt 2021	12	12	1.00	[0.74; 1.00]	1.6%
Tummalapalli 2007	30	30	1.00	[0.88; 1.00]	1.7%
Nalla 2011	20	20	1.00	[0.83; 1.00]	1.7%
Nakano 2021	24	24	1.00	[0.86; 1.00]	1.7%
Nigim 2019a	14	14	1.00	[0.77; 1.00]	1.6%
Nigim 2016	12	12	1.00	[0.74; 1.00]	1.6%
Tummalapalli 2007	30	30	1.00	[0.88; 1.00]	1.7%
**Random effects model**		**436**	**0.95**	**[0.92; 0.97]**	**49.3%**
Heterogeneity: $I^2 = 0\%$, $\tau^2 = 0$, $p = 0.91$					
**incubation_category = 100−199 days**					
Püttmann 2005	5	5	1.00	[0.48; 1.00]	1.6%
Cargioli 2007a	6	6	1.00	[0.54; 1.00]	1.6%
Cargioli 2007b	6	6	1.00	[0.54; 1.00]	1.6%
**Random effects model**		**17**	**0.92**	**[0.70; 0.98]**	**4.7%**
Heterogeneity: $I^2 = 0\%$, $\tau^2 = 0$, $p = 1.00$					
**incubation_category = 31−99 days**					
Chang 2021	40	40	1.00	[0.91; 1.00]	1.7%
**incubation_category = 14−30 days**					
Chen 2021	12	12	1.00	[0.74; 1.00]	1.6%
Karsy 2016	54	54	1.00	[0.93; 1.00]	1.7%
La Cava 2018a	22	24	0.92	[0.73; 0.99]	6.3%
La Cava 2018b	18	23	0.78	[0.56; 0.93]	13.3%
van Furth 2003	15	15	1.00	[0.78; 1.00]	1.7%
Gogineni 2010	20	20	1.00	[0.83; 1.00]	1.7%
Wilisch−Neumann 2013	10	10	1.00	[0.69; 1.00]	1.6%
Pachow 2013	10	10	1.00	[0.69; 1.00]	1.6%
**Random effects model**		**168**	**0.93**	**[0.85; 0.97]**	**29.5%**
Heterogeneity: $I^2 = 26\%$, $\tau^2 = 0.3858$, $p = 0.22$					
**incubation_category = 0−14 days**					
Tuchen 2016	35	35	1.00	[0.90; 1.00]	1.7%
Iwami 2015	5	5	1.00	[0.48; 1.00]	1.6%
Wilisch−Neumann 2013	30	30	1.00	[0.88; 1.00]	1.7%
Iwami 2013	24	24	1.00	[0.86; 1.00]	1.7%
Pachow 2013	16	16	1.00	[0.79; 1.00]	1.7%
Kliese 2013	8	8	1.00	[0.63; 1.00]	1.6%
Ragel 2008c	15	15	1.00	[0.78; 1.00]	1.7%
van Furth 2003	15	15	1.00	[0.78; 1.00]	1.7%
P2011	10	10	1.00	[0.69; 1.00]	1.6%
**Random effects model**		**158**	**0.97**	**[0.92; 0.99]**	**14.8%**
Heterogeneity: $I^2 = 0\%$, $\tau^2 = 0$, $p = 0.99$					
**Random effects model**		**819**	**0.94**	**[0.92; 0.96]**	**100.0%**

Heterogeneity: $I^2 = 0\%$, $\tau^2 = 0$, $p = 0.92$

Test for subgroup differences: $\chi^2_4 = 2.98$, df = 4 ($p = 0.56$)

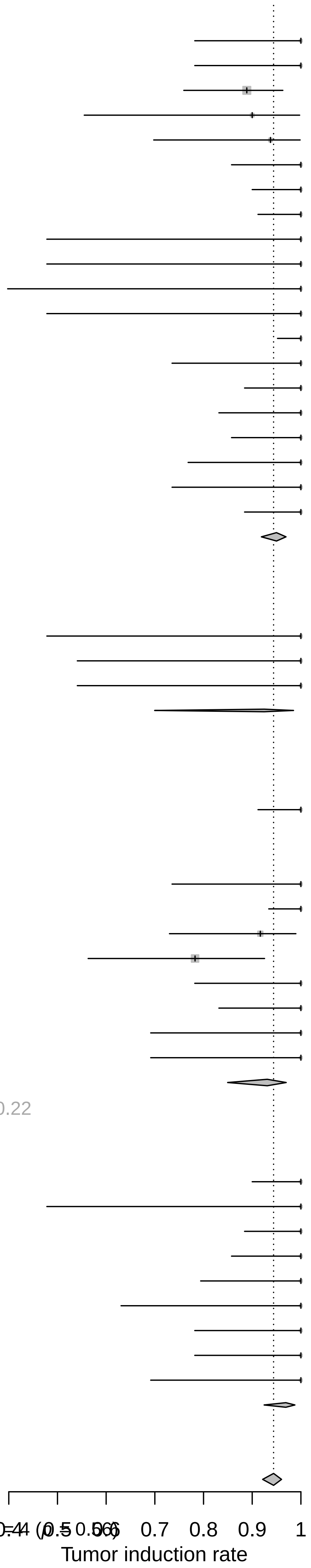

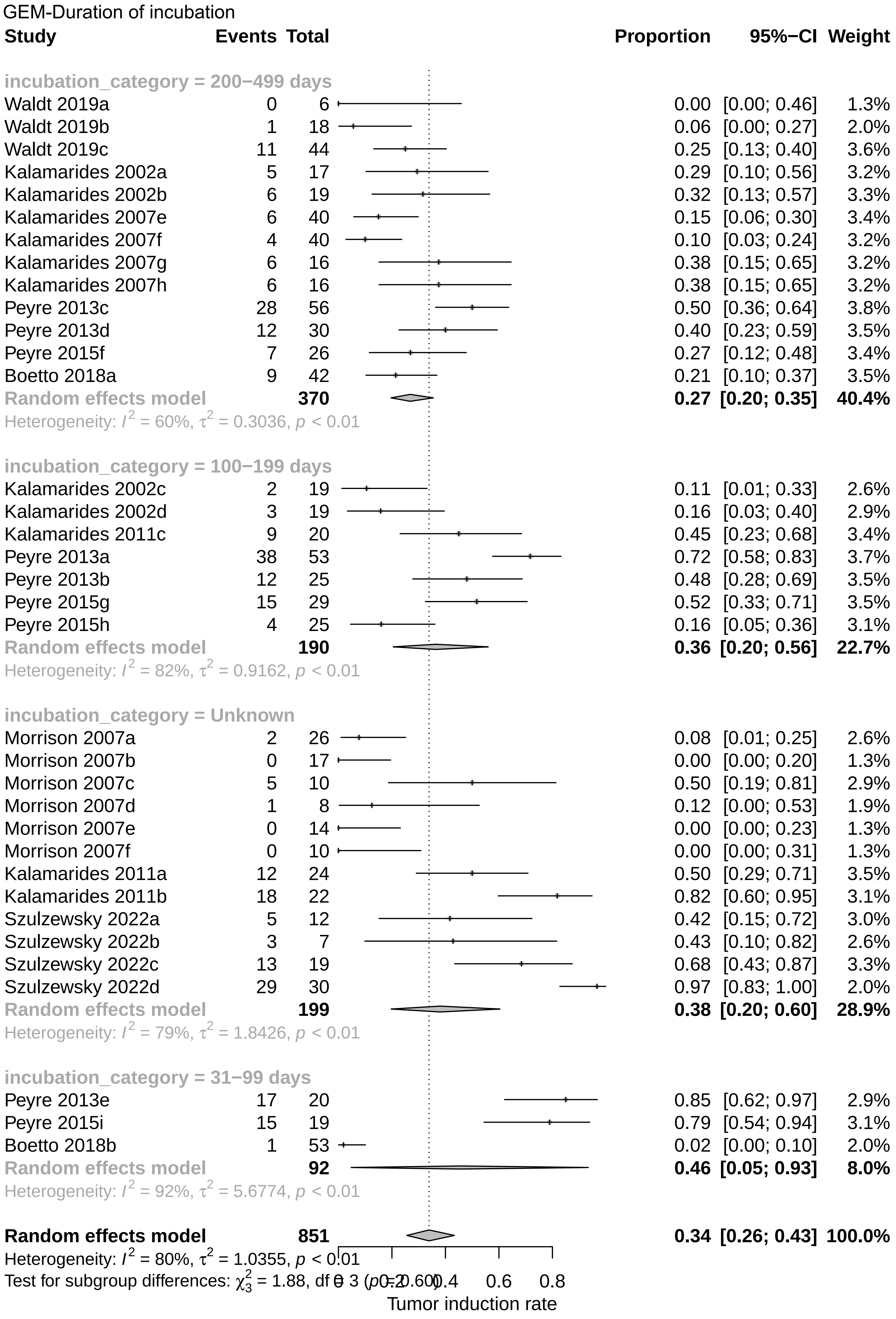

GEM-Duration of incubation
Study Events Total Proportion 95%−CI Weight
incubation_category = 200−499 days
Waldt 2019a 0 6 0.00 [0.00; 0.46] 1.3%
Waldt 2019b 1 18 0.06 [0.00; 0.27] 2.0%
Waldt 2019c 11 44 0.25 [0.13; 0.40] 3.6%
Kalamarides 2002a 5 17 0.29 [0.10; 0.56] 3.2%
Kalamarides 2002b 6 19 0.32 [0.13; 0.57] 3.3%
Kalamarides 2007e 6 40 0.15 [0.06; 0.30] 3.4%
Kalamarides 2007f 4 40 0.10 [0.03; 0.24] 3.2%
Kalamarides 2007g 6 16 0.38 [0.15; 0.65] 3.2%
Kalamarides 2007h 6 16 0.38 [0.15; 0.65] 3.2%
Peyre 2013c 28 56 0.50 [0.36; 0.64] 3.8%
Peyre 2013d 12 30 0.40 [0.23; 0.59] 3.5%
Peyre 2015f 7 26 0.27 [0.12; 0.48] 3.4%
Boetto 2018a 9 42 0.21 [0.10; 0.37] 3.5%
Random effects model 370 0.27 [0.20; 0.35] 40.4%
Heterogeneity: I^2 = 60%, τ^2 = 0.3036, p < 0.01
incubation_category = 100−199 days
Kalamarides 2002c 2 19 0.11 [0.01; 0.33] 2.6%
Kalamarides 2002d 3 19 0.16 [0.03; 0.40] 2.9%
Kalamarides 2011c 9 20 0.45 [0.23; 0.68] 3.4%
Peyre 2013a 38 53 0.72 [0.58; 0.83] 3.7%
Peyre 2013b 12 25 0.48 [0.28; 0.69] 3.5%
Peyre 2015g 15 29 0.52 [0.33; 0.71] 3.5%
Peyre 2015h 4 25 0.16 [0.05; 0.36] 3.1%
Random effects model 190 0.36 [0.20; 0.56] 22.7%
Heterogeneity: I^2 = 82%, τ^2 = 0.9162, p < 0.01
incubation_category = Unknown
Morrison 2007a 2 26 0.08 [0.01; 0.25] 2.6%
Morrison 2007b 0 17 0.00 [0.00; 0.20] 1.3%
Morrison 2007c 5 10 0.50 [0.19; 0.81] 2.9%
Morrison 2007d 1 8 0.12 [0.00; 0.53] 1.9%
Morrison 2007e 0 14 0.00 [0.00; 0.23] 1.3%
Morrison 2007f 0 10 0.00 [0.00; 0.31] 1.3%
Kalamarides 2011a 12 24 0.50 [0.29; 0.71] 3.5%
Kalamarides 2011b 18 22 0.82 [0.60; 0.95] 3.1%
Szulzewsky 2022a 5 12 0.42 [0.15; 0.72] 3.0%
Szulzewsky 2022b 3 7 0.43 [0.10; 0.82] 2.6%
Szulzewsky 2022c 13 19 0.68 [0.43; 0.87] 3.3%
Szulzewsky 2022d 29 30 0.97 [0.83; 1.00] 2.0%
Random effects model 199 0.38 [0.20; 0.60] 28.9%
Heterogeneity: I^2 = 79%, τ^2 = 1.8426, p < 0.01
incubation_category = 31−99 days
Peyre 2013e 17 20 0.85 [0.62; 0.97] 2.9%
Peyre 2015i 15 19 0.79 [0.54; 0.94] 3.1%
Boetto 2018b 1 53 0.02 [0.00; 0.10] 2.0%
Random effects model 92 0.46 [0.05; 0.93] 8.0%
Heterogeneity: I^2 = 92%, τ^2 = 5.6774, p < 0.01
Random effects model 851 0.34 [0.26; 0.43] 100.0%
Heterogeneity: I^2 = 80%, τ^2 = 1.0355, p < 0.01
Test for subgroup differences: χ^2_3 = 1.88, df = 3 (p = 0.60)
0 0.2 0.4 0.6 0.8
Tumor induction rate

PTM-hetero-duration of incubation

Study	Events	Total	Proportion	95%−CI	Weight
**incubation_category = 14−30 days**					
Jiang 2017a	4	4	1.00	[0.40; 1.00]	2.2%
Jiang 2017b	4	4	1.00	[0.40; 1.00]	2.2%
Jiang 2017c	4	4	1.00	[0.40; 1.00]	2.2%
Jiang 2017d	4	4	1.00	[0.40; 1.00]	2.2%
Jiang 2017e	4	4	1.00	[0.40; 1.00]	2.2%
Jiang 2017f	4	4	1.00	[0.40; 1.00]	2.2%
Jiang 2017g	4	4	1.00	[0.40; 1.00]	2.2%
Ding 2015	6	6	1.00	[0.54; 1.00]	2.2%
Schrell 1997	50	50	1.00	[0.93; 1.00]	2.3%
Matsuda 1994a	19	19	1.00	[0.82; 1.00]	2.3%
Matsuda 1994b	12	12	1.00	[0.74; 1.00]	2.3%
Matsuda 1994c	20	20	1.00	[0.83; 1.00]	2.3%
**Random effects model**		**135**	**0.94**	**[0.88; 0.97]**	**26.4%**
Heterogeneity: $I^2 = 0\%$, $\tau^2 = 0$, $p = 0.98$					
**incubation_category = 31−99 days**					
Ragel 2008a	5	5	1.00	[0.48; 1.00]	2.2%
Ragel 2008b	5	5	1.00	[0.48; 1.00]	2.2%
Ragel 2008c	0	5	0.00	[0.00; 0.52]	2.2%
Ragel 2008d	5	5	1.00	[0.48; 1.00]	2.2%
Chen 2007	17	20	0.85	[0.62; 0.97]	5.3%
Ragel 2006a	10	10	1.00	[0.69; 1.00]	2.2%
McCutcheon 2001a	20	20	1.00	[0.83; 1.00]	2.3%
McCutcheon 2001b	8	8	1.00	[0.63; 1.00]	2.2%
McCutcheon 2001c	2	2	1.00	[0.16; 1.00]	2.0%
Yazaki 1995a	36	36	1.00	[0.90; 1.00]	2.3%
Yazaki 1995b	20	20	1.00	[0.83; 1.00]	2.3%
Olson 1987	6	6	1.00	[0.54; 1.00]	2.2%
**Random effects model**		**142**	**0.91**	**[0.80; 0.96]**	**29.6%**
Heterogeneity: $I^2 = 29\%$, $\tau^2 = 0.6779$, $p = 0.16$					
**incubation_category = Unknown**					
Hu 2012a	3	6	0.50	[0.12; 0.88]	4.3%
Hu 2012b	3	5	0.60	[0.15; 0.95]	3.9%
Hu 2012c	4	6	0.67	[0.22; 0.96]	4.1%
**Random effects model**		**17**	**0.59**	**[0.35; 0.79]**	**12.4%**
Heterogeneity: $I^2 = 0\%$, $\tau^2 = 0$, $p = 0.84$					
**incubation_category = 200−499 days**					
Malham 2001a	2	4	0.50	[0.07; 0.93]	3.6%
Malham 2001b	3	3	1.00	[0.29; 1.00]	2.1%
Malham 2001c	4	6	0.67	[0.22; 0.96]	4.1%
**Random effects model**		**13**	**0.65**	**[0.37; 0.86]**	**9.8%**
Heterogeneity: $I^2 = 0\%$, $\tau^2 = 0$, $p = 0.56$					
**incubation_category = 100−199 days**					
Malham 2001d	5	5	1.00	[0.48; 1.00]	2.2%
Malham 2001e	4	6	0.67	[0.22; 0.96]	4.1%
Malham 2001f	0	6	0.00	[0.00; 0.46]	2.2%
Malham 2001g	7	10	0.70	[0.35; 0.93]	5.0%
Malham 2001h	14	20	0.70	[0.46; 0.88]	6.0%
Jensen 2001	76	76	1.00	[0.95; 1.00]	2.3%
**Random effects model**		**123**	**0.75**	**[0.45; 0.92]**	**21.8%**
Heterogeneity: $I^2 = 67\%$, $\tau^2 = 1.5949$, $p < 0.01$					
**Random effects model**		**430**	**0.85**	**[0.78; 0.91]**	**100.0%**

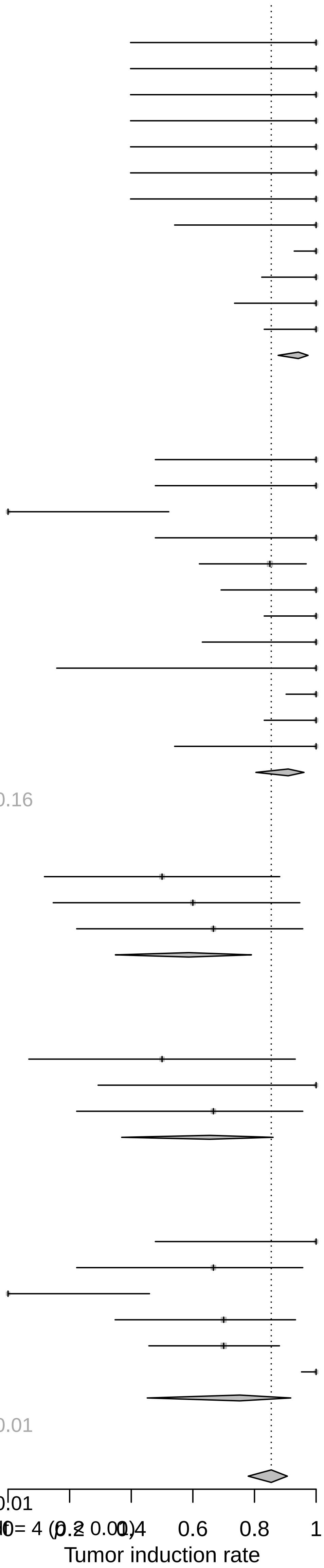

Heterogeneity: $I^2 = 41\%$, $\tau^2 = 0.8685$, $p < 0.01$

Test for subgroup differences: $\chi^2_4 = 19.65$, df = 4 ($p < 0.01$)

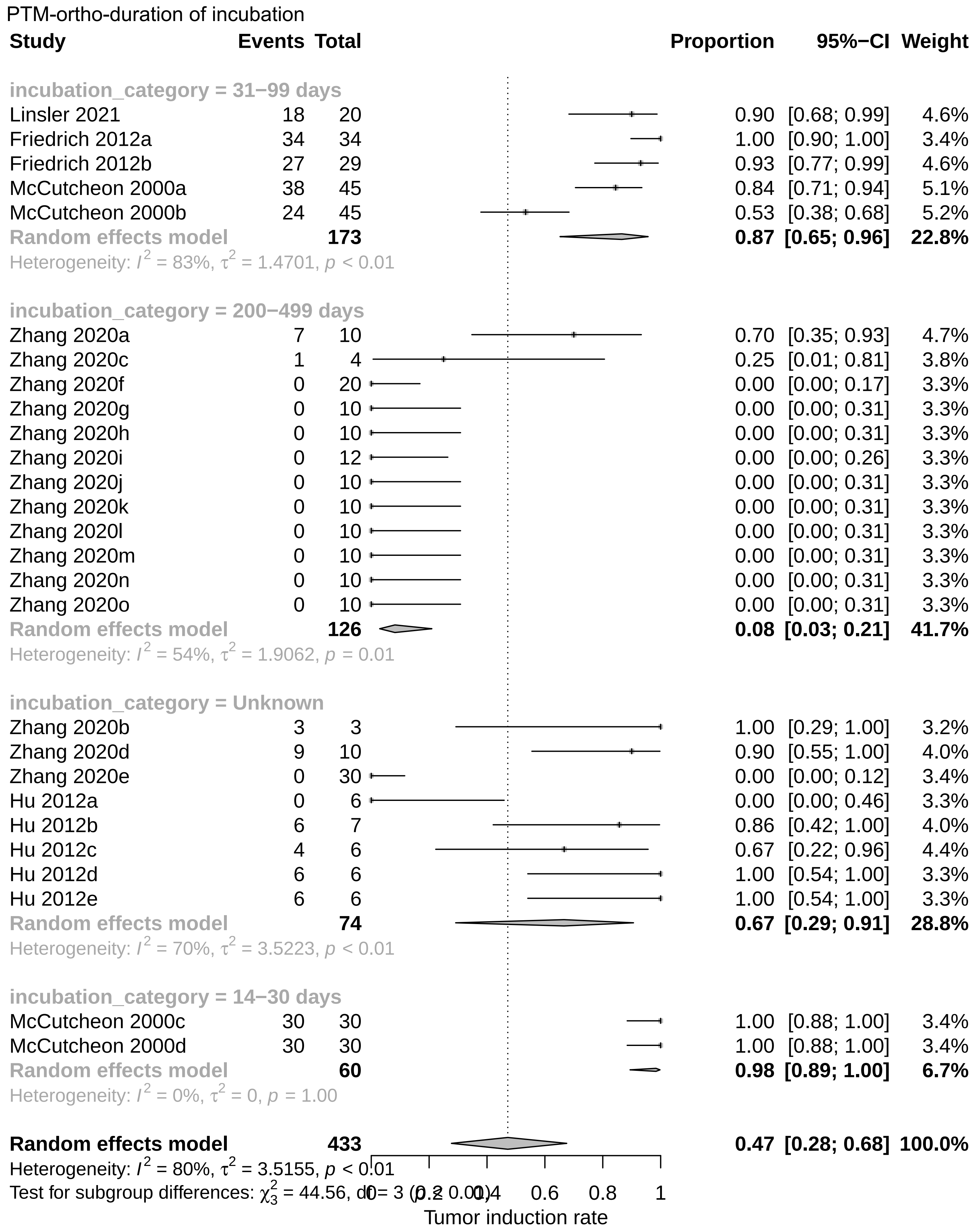
PTM-ortho-duration of incubation
Study Events Total Proportion 95%−CI Weight
incubation_category = 31−99 days
Linsler 2021 18 20 0.90 [0.68; 0.99] 4.6%
Friedrich 2012a 34 34 1.00 [0.90; 1.00] 3.4%
Friedrich 2012b 27 29 0.93 [0.77; 0.99] 4.6%
McCutcheon 2000a 38 45 0.84 [0.71; 0.94] 5.1%
McCutcheon 2000b 24 45 0.53 [0.38; 0.68] 5.2%
Random effects model 173 0.87 [0.65; 0.96] 22.8%
Heterogeneity: I^2 = 83%, τ^2 = 1.4701, p < 0.01
incubation_category = 200−499 days
Zhang 2020a 7 10 0.70 [0.35; 0.93] 4.7%
Zhang 2020c 1 4 0.25 [0.01; 0.81] 3.8%
Zhang 2020f 0 20 0.00 [0.00; 0.17] 3.3%
Zhang 2020g 0 10 0.00 [0.00; 0.31] 3.3%
Zhang 2020h 0 10 0.00 [0.00; 0.31] 3.3%
Zhang 2020i 0 12 0.00 [0.00; 0.26] 3.3%
Zhang 2020j 0 10 0.00 [0.00; 0.31] 3.3%
Zhang 2020k 0 10 0.00 [0.00; 0.31] 3.3%
Zhang 2020l 0 10 0.00 [0.00; 0.31] 3.3%
Zhang 2020m 0 10 0.00 [0.00; 0.31] 3.3%
Zhang 2020n 0 10 0.00 [0.00; 0.31] 3.3%
Zhang 2020o 0 10 0.00 [0.00; 0.31] 3.3%
Random effects model 126 0.08 [0.03; 0.21] 41.7%
Heterogeneity: I^2 = 54%, τ^2 = 1.9062, p = 0.01
incubation_category = Unknown
Zhang 2020b 3 3 1.00 [0.29; 1.00] 3.2%
Zhang 2020d 9 10 0.90 [0.55; 1.00] 4.0%
Zhang 2020e 0 30 0.00 [0.00; 0.12] 3.4%
Hu 2012a 0 6 0.00 [0.00; 0.46] 3.3%
Hu 2012b 6 7 0.86 [0.42; 1.00] 4.0%
Hu 2012c 4 6 0.67 [0.22; 0.96] 4.4%
Hu 2012d 6 6 1.00 [0.54; 1.00] 3.3%
Hu 2012e 6 6 1.00 [0.54; 1.00] 3.3%
Random effects model 74 0.67 [0.29; 0.91] 28.8%
Heterogeneity: I^2 = 70%, τ^2 = 3.5223, p < 0.01
incubation_category = 14−30 days
McCutcheon 2000c 30 30 1.00 [0.88; 1.00] 3.4%
McCutcheon 2000d 30 30 1.00 [0.88; 1.00] 3.4%
Random effects model 60 0.98 [0.89; 1.00] 6.7%
Heterogeneity: I^2 = 0%, τ^2 = 0, p = 1.00
Random effects model 433 0.47 [0.28; 0.68] 100.0%
Heterogeneity: I^2 = 80%, τ^2 = 3.5155, p < 0.01
Test for subgroup differences: χ^2_3 = 44.56, df = 3 (p < 0.01)
0 0.2 0.4 0.6 0.8 1
Tumor induction rate

ECLM-hetero-number of cells

Study	Events	Total	Proportion	95%−CI	Weight
**n_cells_category = 1,000,000−10,000,000**					
Senol 2015a	30	30	1.00	[0.88; 1.00]	1.8%
Soto−Montenegro 2014	16	16	1.00	[0.79; 1.00]	1.7%
Choudhury 2022	20	20	1.00	[0.83; 1.00]	1.8%
Kanno 2013	10	10	1.00	[0.69; 1.00]	1.7%
Guo 2021	28	28	1.00	[0.88; 1.00]	1.8%
Zhang 2020	64	64	1.00	[0.94; 1.00]	1.8%
Wilisch−Neumann 2013	15	15	1.00	[0.78; 1.00]	1.7%
Pachow 2013	15	15	1.00	[0.78; 1.00]	1.7%
Kliese 2013	14	14	1.00	[0.77; 1.00]	1.7%
Ge 2016	8	8	1.00	[0.63; 1.00]	1.7%
Kargiotis 2008	40	40	1.00	[0.91; 1.00]	1.8%
Kondraganti 2006	30	30	1.00	[0.88; 1.00]	1.8%
John 2021	15	15	1.00	[0.78; 1.00]	1.7%
Deng 2022	10	10	1.00	[0.69; 1.00]	1.7%
Bao 2021	35	35	1.00	[0.90; 1.00]	1.8%
Tanaka 1989	3	3	1.00	[0.29; 1.00]	1.6%
Jungwirth 2021	30	30	1.00	[0.88; 1.00]	1.8%
**Random effects model**		**383**	**0.97**	**[0.95; 0.99]**	**29.6%**
Heterogeneity: $I^2 = 0\%$, $\tau^2 = 0$, $p = 1.00$					
**n_cells_category = 100,000−500,000**					
Senol 2015b	16	16	1.00	[0.79; 1.00]	1.7%
Ragel 2006	15	15	1.00	[0.78; 1.00]	1.7%
Takeda 2017a	6	6	1.00	[0.54; 1.00]	1.7%
Kim 2020a	8	8	1.00	[0.63; 1.00]	1.7%
Iwami 2015a	10	10	1.00	[0.69; 1.00]	1.7%
Iwami 2015b	10	10	1.00	[0.69; 1.00]	1.7%
Ragel 2006a	55	55	1.00	[0.94; 1.00]	1.8%
Saydam 2009	24	24	1.00	[0.86; 1.00]	1.8%
**Random effects model**		**144**	**0.97**	**[0.92; 0.99]**	**13.9%**
Heterogeneity: $I^2 = 0\%$, $\tau^2 = 0$, $p = 0.98$					
**n_cells_category = Unknown**					
Gal−Gonzalez 2018	6	6	1.00	[0.54; 1.00]	1.7%
Ragel 2008a	10	10	1.00	[0.69; 1.00]	1.7%
Yazaki 1995	40	40	1.00	[0.91; 1.00]	1.8%
Ishiwata 2004	3	3	1.00	[0.29; 1.00]	1.6%
Wang 2016	23	30	0.77	[0.58; 0.90]	19.3%
Ragel 2008	19	20	0.95	[0.75; 1.00]	3.4%
Lee 1990	10	10	1.00	[0.69; 1.00]	1.7%
Chen 2021	16	16	1.00	[0.79; 1.00]	1.7%
**Random effects model**		**135**	**0.92**	**[0.82; 0.97]**	**33.0%**
Heterogeneity: $I^2 = 26\%$, $\tau^2 = 0.4290$, $p = 0.22$					
**n_cells_category = 10,001−100,000**					
Ding 2022a	48	48	1.00	[0.93; 1.00]	1.8%
Ding 2022b	48	48	1.00	[0.93; 1.00]	1.8%
Kim 2020b	6	6	1.00	[0.54; 1.00]	1.7%
**Random effects model**		**102**	**0.98**	**[0.91; 1.00]**	**5.2%**
Heterogeneity: $I^2 = 0\%$, $\tau^2 = 0$, $p = 0.53$					
**n_cells_category = 500,000−1,000,000**					
Takeda 2017b	9	9	1.00	[0.66; 1.00]	1.7%
Park 2015	8	8	1.00	[0.63; 1.00]	1.7%
Haase 2010	13	15	0.87	[0.60; 0.98]	6.2%
Gupta 2007	10	10	1.00	[0.69; 1.00]	1.7%
Ragel 2006b	30	30	1.00	[0.88; 1.00]	1.8%
Surace 2004	16	16	1.00	[0.79; 1.00]	1.7%
Jungwirth 2019	6	6	1.00	[0.54; 1.00]	1.7%
**Random effects model**		**94**	**0.94**	**[0.85; 0.97]**	**16.6%**
Heterogeneity: $I^2 = 0\%$, $\tau^2 = 0$, $p = 0.86$					
**n_cells_category = >10,000,000**					
Wang 2018	9	9	1.00	[0.66; 1.00]	1.7%
**Random effects model**		**867**	**0.95**	**[0.93; 0.96]**	**100.0%**

Heterogeneity: $I^2 = 0\%$, $\tau^2 = 0$, $p = 0.84$

Test for subgroup differences: $\chi^2_5 = 6.65$, df = 5 ($p = 0.25$)

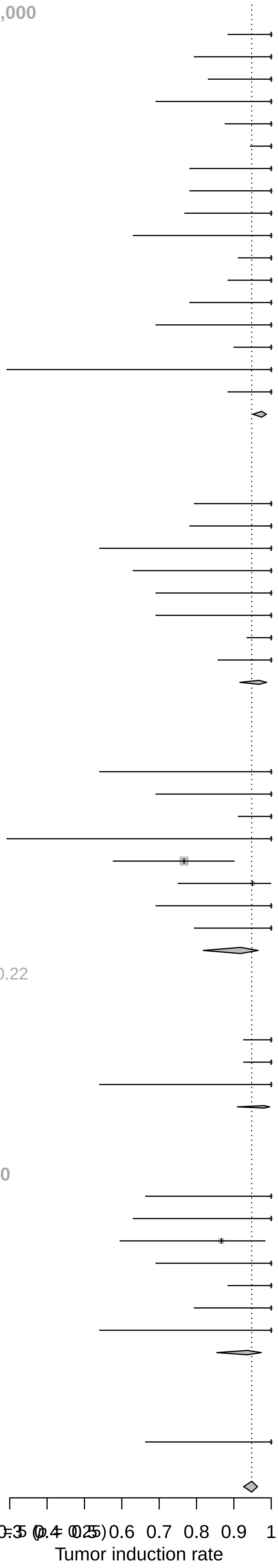

ECLM-ortho-number of cells

Study	Events	Total	Proportion	95%−CI	Weight
**n_cells_category = 500,000−1,000,000**					
McCutcheon 2000a	15	15	1.00	[0.78; 1.00]	1.7%
McCutcheon 2000b	15	15	1.00	[0.78; 1.00]	1.7%
Püttmann 2005	5	5	1.00	[0.48; 1.00]	1.6%
van Furth 2003	15	15	1.00	[0.78; 1.00]	1.7%
Salhia 2002	75	75	1.00	[0.95; 1.00]	1.7%
van Furth 2003	15	15	1.00	[0.78; 1.00]	1.7%
P2011	10	10	1.00	[0.69; 1.00]	1.6%
Cargioli 2007a	6	6	1.00	[0.54; 1.00]	1.6%
Cargioli 2007b	6	6	1.00	[0.54; 1.00]	1.6%
**Random effects model**		**162**	**0.96**	**[0.91; 0.99]**	**14.7%**
Heterogeneity: $I^2 = 0\%$, $\tau^2 = 0$, $p = 0.97$					
**n_cells_category = Unknown**					
Chang 2021	40	40	1.00	[0.91; 1.00]	1.7%
Ragel 2008a	40	45	0.89	[0.76; 0.96]	15.2%
Ragel 2008b	9	10	0.90	[0.55; 1.00]	3.1%
La Cava 2018a	22	24	0.92	[0.73; 0.99]	6.3%
La Cava 2018b	18	23	0.78	[0.56; 0.93]	13.3%
Yazaki 1995	15	16	0.94	[0.70; 1.00]	3.2%
Ragel 2008c	15	15	1.00	[0.78; 1.00]	1.7%
**Random effects model**		**173**	**0.89**	**[0.82; 0.94]**	**44.4%**
Heterogeneity: $I^2 = 9\%$, $\tau^2 = 0.0606$, $p = 0.36$					
**n_cells_category = 10,001−100,000**					
Chen 2021	12	12	1.00	[0.74; 1.00]	1.6%
Karsy 2016	54	54	1.00	[0.93; 1.00]	1.7%
Ho 2018	40	40	1.00	[0.91; 1.00]	1.7%
Iwami 2013	24	24	1.00	[0.86; 1.00]	1.7%
Kliese 2013	8	8	1.00	[0.63; 1.00]	1.6%
Baia 2007c	4	4	1.00	[0.40; 1.00]	1.5%
Baia 2007d	5	5	1.00	[0.48; 1.00]	1.6%
Gogineni 2010	20	20	1.00	[0.83; 1.00]	1.7%
Nalla 2011	20	20	1.00	[0.83; 1.00]	1.7%
Nigim 2019a	14	14	1.00	[0.77; 1.00]	1.6%
Nigim 2016	12	12	1.00	[0.74; 1.00]	1.6%
**Random effects model**		**213**	**0.97**	**[0.93; 0.99]**	**18.0%**
Heterogeneity: $I^2 = 0\%$, $\tau^2 = 0$, $p = 0.99$					
**n_cells_category = 100,000−500,000**					
Nakano 2022	24	24	1.00	[0.86; 1.00]	1.7%
Bao 2021	35	35	1.00	[0.90; 1.00]	1.7%
Tuchen 2016	35	35	1.00	[0.90; 1.00]	1.7%
Iwami 2015	5	5	1.00	[0.48; 1.00]	1.6%
Wilisch−Neumann 2013	30	30	1.00	[0.88; 1.00]	1.7%
Pachow 2013	16	16	1.00	[0.79; 1.00]	1.7%
Baia 2007b	5	5	1.00	[0.48; 1.00]	1.6%
Waldt 2021	12	12	1.00	[0.74; 1.00]	1.6%
Tummalapalli 2007	30	30	1.00	[0.88; 1.00]	1.7%
Nakano 2021	24	24	1.00	[0.86; 1.00]	1.7%
Wilisch−Neumann 2013	10	10	1.00	[0.69; 1.00]	1.6%
Pachow 2013	10	10	1.00	[0.69; 1.00]	1.6%
Tummalapalli 2007	30	30	1.00	[0.88; 1.00]	1.7%
**Random effects model**		**266**	**0.97**	**[0.94; 0.99]**	**21.4%**
Heterogeneity: $I^2 = 0\%$, $\tau^2 = 0$, $p = 1.00$					
**n_cells_category = 1,000,000−10,000,000**					
Baia 2007a	5	5	1.00	[0.48; 1.00]	1.6%
**Random effects model**		**819**	**0.94**	**[0.92; 0.96]**	**100.0%**

Heterogeneity: $I^2 = 0\%$, $\tau^2 = 0$, $p = 0.92$

Test for subgroup differences: $\chi^2_4 = 11.87$, df = 4 ($p = 0.02$)

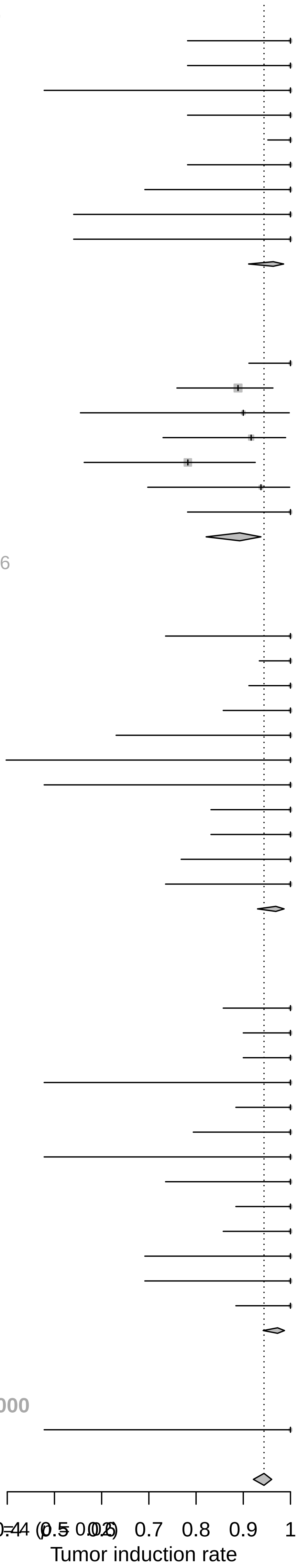

PTM-hetero-number of cells

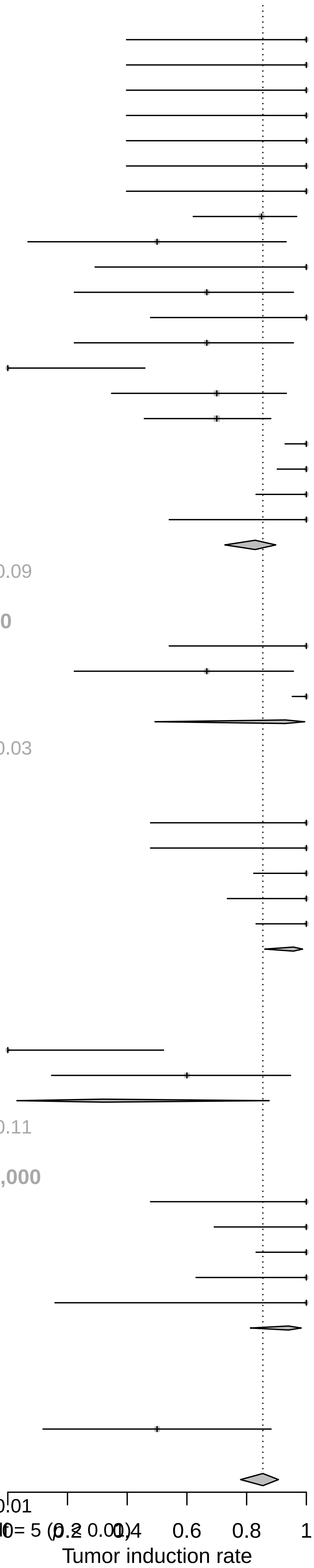

Study	Events	Total	Proportion	95%−CI	Weight
**n_cells_category = Unknown**					
Jiang 2017a	4	4	1.00	[0.40; 1.00]	2.2%
Jiang 2017b	4	4	1.00	[0.40; 1.00]	2.2%
Jiang 2017c	4	4	1.00	[0.40; 1.00]	2.2%
Jiang 2017d	4	4	1.00	[0.40; 1.00]	2.2%
Jiang 2017e	4	4	1.00	[0.40; 1.00]	2.2%
Jiang 2017f	4	4	1.00	[0.40; 1.00]	2.2%
Jiang 2017g	4	4	1.00	[0.40; 1.00]	2.2%
Chen 2007	17	20	0.85	[0.62; 0.97]	5.3%
Malham 2001a	2	4	0.50	[0.07; 0.93]	3.6%
Malham 2001b	3	3	1.00	[0.29; 1.00]	2.1%
Malham 2001c	4	6	0.67	[0.22; 0.96]	4.1%
Malham 2001d	5	5	1.00	[0.48; 1.00]	2.2%
Malham 2001e	4	6	0.67	[0.22; 0.96]	4.1%
Malham 2001f	0	6	0.00	[0.00; 0.46]	2.2%
Malham 2001g	7	10	0.70	[0.35; 0.93]	5.0%
Malham 2001h	14	20	0.70	[0.46; 0.88]	6.0%
Schrell 1997	50	50	1.00	[0.93; 1.00]	2.3%
Yazaki 1995a	36	36	1.00	[0.90; 1.00]	2.3%
Yazaki 1995b	20	20	1.00	[0.83; 1.00]	2.3%
Olson 1987	6	6	1.00	[0.54; 1.00]	2.2%
**Random effects model**		**220**	**0.83**	**[0.73; 0.90]**	**58.7%**
Heterogeneity: $I^2 = 32\%$, $\tau^2 = 0.5198$, $p = 0.09$					
**n_cells_category = 500,000−1,000,000**					
Ding 2015	6	6	1.00	[0.54; 1.00]	2.2%
Hu 2012c	4	6	0.67	[0.22; 0.96]	4.1%
Jensen 2001	76	76	1.00	[0.95; 1.00]	2.3%
**Random effects model**		**88**	**0.93**	**[0.49; 0.99]**	**8.6%**
Heterogeneity: $I^2 = 71\%$, $\tau^2 = 3.7837$, $p = 0.03$					
**n_cells_category = >10,000,000**					
Ragel 2008a	5	5	1.00	[0.48; 1.00]	2.2%
Ragel 2008b	5	5	1.00	[0.48; 1.00]	2.2%
Matsuda 1994a	19	19	1.00	[0.82; 1.00]	2.3%
Matsuda 1994b	12	12	1.00	[0.74; 1.00]	2.3%
Matsuda 1994c	20	20	1.00	[0.83; 1.00]	2.3%
**Random effects model**		**61**	**0.96**	**[0.86; 0.99]**	**11.2%**
Heterogeneity: $I^2 = 0\%$, $\tau^2 = 0$, $p = 0.94$					
**n_cells_category = 10,001−100,000**					
Ragel 2008c	0	5	0.00	[0.00; 0.52]	2.2%
Hu 2012b	3	5	0.60	[0.15; 0.95]	3.9%
**Random effects model**		**10**	**0.32**	**[0.03; 0.88]**	**6.1%**
Heterogeneity: $I^2 = 62\%$, $\tau^2 = 2.4218$, $p = 0.11$					
**n_cells_category = 1,000,000−10,000,000**					
Ragel 2008d	5	5	1.00	[0.48; 1.00]	2.2%
Ragel 2006a	10	10	1.00	[0.69; 1.00]	2.2%
McCutcheon 2001a	20	20	1.00	[0.83; 1.00]	2.3%
McCutcheon 2001b	8	8	1.00	[0.63; 1.00]	2.2%
McCutcheon 2001c	2	2	1.00	[0.16; 1.00]	2.0%
**Random effects model**		**45**	**0.94**	**[0.81; 0.98]**	**11.0%**
Heterogeneity: $I^2 = 0\%$, $\tau^2 = 0$, $p = 0.89$					
**n_cells_category = 1,001−10,000**					
Hu 2012a	3	6	0.50	[0.12; 0.88]	4.3%
**Random effects model**		**430**	**0.85**	**[0.78; 0.91]**	**100.0%**

Heterogeneity: $I^2 = 41\%$, $\tau^2 = 0.8685$, $p < 0.01$

Test for subgroup differences: $\chi^2_5 = 15.24$, df = 5 ($p < 0.01$)

PTM-ortho-number of cells

Study	Events	Total	Proportion	95%−CI	Weight
**n_cells_category = 500,000−1,000,000**					
Linsler 2021	18	20	0.90	[0.68; 0.99]	4.6%
Friedrich 2012a	34	34	1.00	[0.90; 1.00]	3.4%
Friedrich 2012b	27	29	0.93	[0.77; 0.99]	4.6%
Hu 2012e	6	6	1.00	[0.54; 1.00]	3.3%
McCutcheon 2000a	38	45	0.84	[0.71; 0.94]	5.1%
McCutcheon 2000b	24	45	0.53	[0.38; 0.68]	5.2%
McCutcheon 2000c	30	30	1.00	[0.88; 1.00]	3.4%
McCutcheon 2000d	30	30	1.00	[0.88; 1.00]	3.4%
**Random effects model**		**239**	**0.91**	**[0.77; 0.97]**	**32.8%**
Heterogeneity: $I^2 = 79\%$, $\tau^2 = 1.7206$, $p < 0.01$					
**n_cells_category = 10,001−100,000**					
Zhang 2020a	7	10	0.70	[0.35; 0.93]	4.7%
Zhang 2020b	3	3	1.00	[0.29; 1.00]	3.2%
Zhang 2020c	1	4	0.25	[0.01; 0.81]	3.8%
Zhang 2020d	9	10	0.90	[0.55; 1.00]	4.0%
Zhang 2020e	0	30	0.00	[0.00; 0.12]	3.4%
Zhang 2020f	0	20	0.00	[0.00; 0.17]	3.3%
Zhang 2020g	0	10	0.00	[0.00; 0.31]	3.3%
Zhang 2020h	0	10	0.00	[0.00; 0.31]	3.3%
Zhang 2020i	0	12	0.00	[0.00; 0.26]	3.3%
Zhang 2020j	0	10	0.00	[0.00; 0.31]	3.3%
Zhang 2020k	0	10	0.00	[0.00; 0.31]	3.3%
Zhang 2020l	0	10	0.00	[0.00; 0.31]	3.3%
Zhang 2020m	0	10	0.00	[0.00; 0.31]	3.3%
Zhang 2020n	0	10	0.00	[0.00; 0.31]	3.3%
Zhang 2020o	0	10	0.00	[0.00; 0.31]	3.3%
Hu 2012d	6	6	1.00	[0.54; 1.00]	3.3%
**Random effects model**		**175**	**0.15**	**[0.05; 0.37]**	**55.6%**
Heterogeneity: $I^2 = 71\%$, $\tau^2 = 4.1214$, $p < 0.01$					
**n_cells_category = 0−100**					
Hu 2012a	0	6	0.00	[0.00; 0.46]	3.3%
**n_cells_category = 101−1,000**					
Hu 2012b	6	7	0.86	[0.42; 1.00]	4.0%
**n_cells_category = 1,001−10,000**					
Hu 2012c	4	6	0.67	[0.22; 0.96]	4.4%
**Random effects model**		**433**	**0.47**	**[0.28; 0.68]**	**100.0%**

Heterogeneity: $I^2 = 80\%$, $\tau^2 = 3.5155$, $p < 0.01$

Test for subgroup differences: $\chi^2_4 = 29.17$, df = 4 ($p < 0.01$)

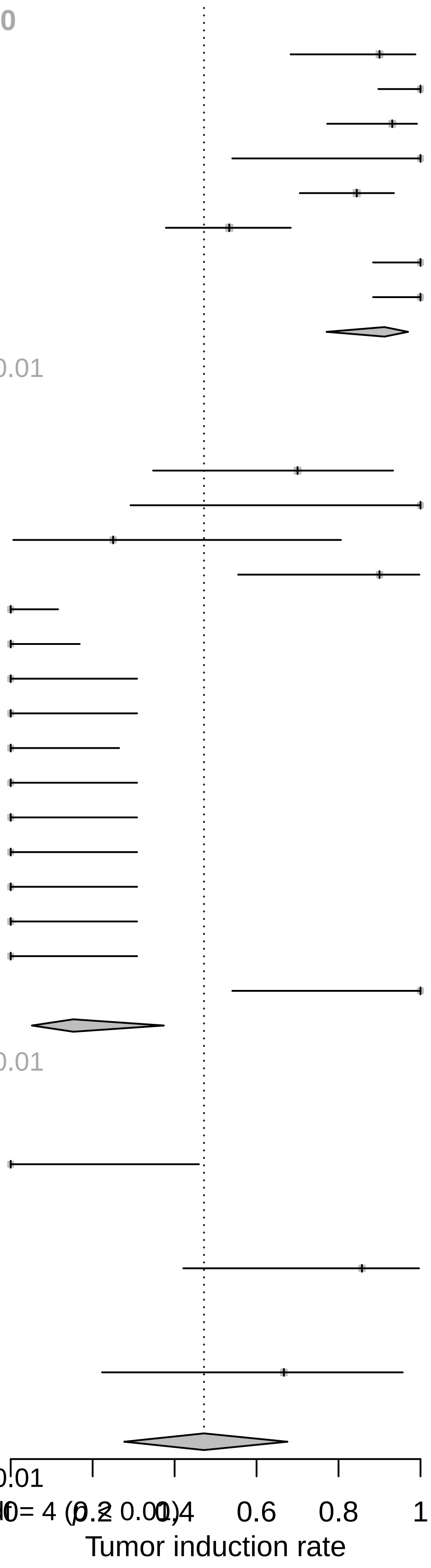

ECLM-hetero-injection volume

Study	Events	Total	Proportion	95%−CI	Weight
**injection_category = 100−250 µl**					
Senol 2015a	30	30	1.00	[0.88; 1.00]	1.8%
Senol 2015b	16	16	1.00	[0.79; 1.00]	1.7%
Soto−Montenegro 2014	16	16	1.00	[0.79; 1.00]	1.7%
Ding 2022a	48	48	1.00	[0.93; 1.00]	1.8%
Ding 2022b	48	48	1.00	[0.93; 1.00]	1.8%
Takeda 2017a	6	6	1.00	[0.54; 1.00]	1.7%
Takeda 2017b	9	9	1.00	[0.66; 1.00]	1.7%
Kanno 2013	10	10	1.00	[0.69; 1.00]	1.7%
Kim 2020a	8	8	1.00	[0.63; 1.00]	1.7%
Kim 2020b	6	6	1.00	[0.54; 1.00]	1.7%
Guo 2021	28	28	1.00	[0.88; 1.00]	1.8%
Wang 2018	9	9	1.00	[0.66; 1.00]	1.7%
Iwami 2015b	10	10	1.00	[0.69; 1.00]	1.7%
Wilisch−Neumann 2013	15	15	1.00	[0.78; 1.00]	1.7%
Pachow 2013	15	15	1.00	[0.78; 1.00]	1.7%
Haase 2010	13	15	0.87	[0.60; 0.98]	6.2%
Kondraganti 2006	30	30	1.00	[0.88; 1.00]	1.8%
Surace 2004	16	16	1.00	[0.79; 1.00]	1.7%
Lee 1990	10	10	1.00	[0.69; 1.00]	1.7%
John 2021	15	15	1.00	[0.78; 1.00]	1.7%
Deng 2022	10	10	1.00	[0.69; 1.00]	1.7%
Chen 2021	16	16	1.00	[0.79; 1.00]	1.7%
Jungwirth 2021	30	30	1.00	[0.88; 1.00]	1.8%
Jungwirth 2019	6	6	1.00	[0.54; 1.00]	1.7%
Saydam 2009	24	24	1.00	[0.86; 1.00]	1.8%
**Random effects model**		**446**	**0.96**	**[0.94; 0.98]**	**47.9%**
Heterogeneity: $I^2 = 0\%$, $\tau^2 = 0$, $p = 1.00$					
**injection_category = Unknown**					
Gal−Gonzalez 2018	6	6	1.00	[0.54; 1.00]	1.7%
Ragel 2006	15	15	1.00	[0.78; 1.00]	1.7%
Choudhury 2022	20	20	1.00	[0.83; 1.00]	1.8%
Yazaki 1995	40	40	1.00	[0.91; 1.00]	1.8%
Ishiwata 2004	3	3	1.00	[0.29; 1.00]	1.6%
Zhang 2020	64	64	1.00	[0.94; 1.00]	1.8%
Wang 2016	23	30	0.77	[0.58; 0.90]	19.3%
Park 2015	8	8	1.00	[0.63; 1.00]	1.7%
Kliese 2013	14	14	1.00	[0.77; 1.00]	1.7%
Ge 2016	8	8	1.00	[0.63; 1.00]	1.7%
Kargiotis 2008	40	40	1.00	[0.91; 1.00]	1.8%
Gupta 2007	10	10	1.00	[0.69; 1.00]	1.7%
Ragel 2006a	55	55	1.00	[0.94; 1.00]	1.8%
Ragel 2006b	30	30	1.00	[0.88; 1.00]	1.8%
Bao 2021	35	35	1.00	[0.90; 1.00]	1.8%
Tanaka 1989	3	3	1.00	[0.29; 1.00]	1.6%
**Random effects model**		**381**	**0.96**	**[0.91; 0.98]**	**45.2%**
Heterogeneity: $I^2 = 34\%$, $\tau^2 = 0.7780$, $p = 0.09$					
**injection_category = 251−500 µl**					
Ragel 2008a	10	10	1.00	[0.69; 1.00]	1.7%
Ragel 2008	19	20	0.95	[0.75; 1.00]	3.4%
**Random effects model**		**30**	**0.95**	**[0.79; 0.99]**	**5.1%**
Heterogeneity: $I^2 = 0\%$, $\tau^2 = 0$, $p = 0.96$					
**injection_category = 0−99 µl**					
Iwami 2015a	10	10	1.00	[0.69; 1.00]	1.7%
**Random effects model**		**867**	**0.95**	**[0.93; 0.96]**	**100.0%**
Heterogeneity: $I^2 = 0\%$, $\tau^2 = 0$, $p = 0.84$					
Test for subgroup differences: $\chi^2_3 = 0.12$, df = 3 ($p = 0.99$)					

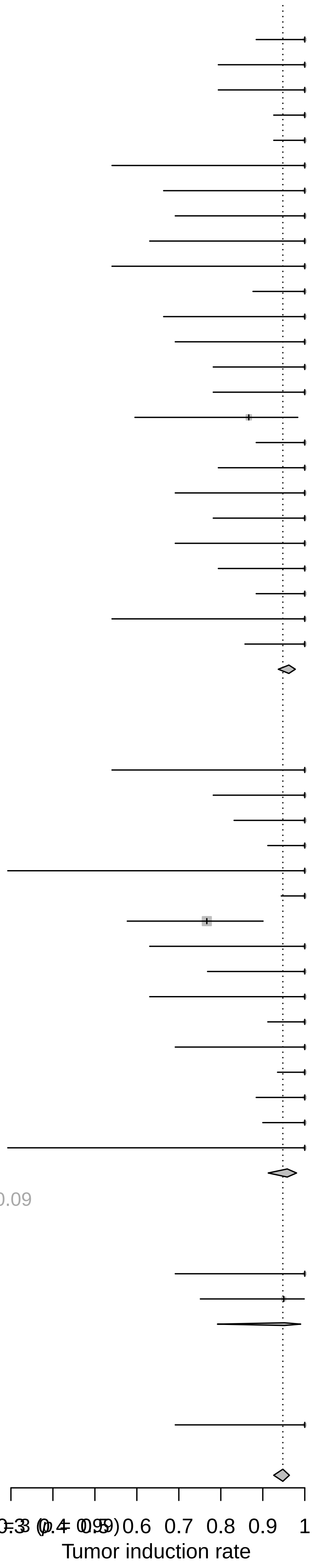

ECLM-ortho-injection volume

Study	Events	Total	Proportion	95%−CI	Weight
**injection_category = 6−10 µl**					
McCutcheon 2000a	15	15	1.00	[0.78; 1.00]	1.7%
McCutcheon 2000b	15	15	1.00	[0.78; 1.00]	1.7%
Karsy 2016	54	54	1.00	[0.93; 1.00]	1.7%
Gogineni 2010	20	20	1.00	[0.83; 1.00]	1.7%
Tummalapalli 2007	30	30	1.00	[0.88; 1.00]	1.7%
Nalla 2011	20	20	1.00	[0.83; 1.00]	1.7%
**Random effects model**		**154**	**0.98**	**[0.94; 0.99]**	**10.0%**
Heterogeneity: $I^2 = 0\%$, $\tau^2 = 0$, $p = 0.99$					
**injection_category = 2−5 µl**					
Püttmann 2005	5	5	1.00	[0.48; 1.00]	1.6%
Chang 2021	40	40	1.00	[0.91; 1.00]	1.7%
Chen 2021	12	12	1.00	[0.74; 1.00]	1.6%
Ragel 2008a	40	45	0.89	[0.76; 0.96]	15.2%
Ragel 2008b	9	10	0.90	[0.55; 1.00]	3.1%
van Furth 2003	15	15	1.00	[0.78; 1.00]	1.7%
Nakano 2022	24	24	1.00	[0.86; 1.00]	1.7%
Ho 2018	40	40	1.00	[0.91; 1.00]	1.7%
Tuchen 2016	35	35	1.00	[0.90; 1.00]	1.7%
Iwami 2015	5	5	1.00	[0.48; 1.00]	1.6%
Wilisch−Neumann 2013	30	30	1.00	[0.88; 1.00]	1.7%
Iwami 2013	24	24	1.00	[0.86; 1.00]	1.7%
Pachow 2013	16	16	1.00	[0.79; 1.00]	1.7%
Kliese 2013	8	8	1.00	[0.63; 1.00]	1.6%
Ragel 2008c	15	15	1.00	[0.78; 1.00]	1.7%
Baia 2007a	5	5	1.00	[0.48; 1.00]	1.6%
Baia 2007b	5	5	1.00	[0.48; 1.00]	1.6%
Salhia 2002	75	75	1.00	[0.95; 1.00]	1.7%
Waldt 2021	12	12	1.00	[0.74; 1.00]	1.6%
van Furth 2003	15	15	1.00	[0.78; 1.00]	1.7%
Nakano 2021	24	24	1.00	[0.86; 1.00]	1.7%
Wilisch−Neumann 2013	10	10	1.00	[0.69; 1.00]	1.6%
Pachow 2013	10	10	1.00	[0.69; 1.00]	1.6%
Cargioli 2007a	6	6	1.00	[0.54; 1.00]	1.6%
Cargioli 2007b	6	6	1.00	[0.54; 1.00]	1.6%
Nigim 2019a	14	14	1.00	[0.77; 1.00]	1.6%
Nigim 2016	12	12	1.00	[0.74; 1.00]	1.6%
**Random effects model**		**518**	**0.95**	**[0.93; 0.97]**	**59.1%**
Heterogeneity: $I^2 = 0\%$, $\tau^2 = 0$, $p = 0.99$					
**injection_category = Unknown**					
La Cava 2018a	22	24	0.92	[0.73; 0.99]	6.3%
La Cava 2018b	18	23	0.78	[0.56; 0.93]	13.3%
Yazaki 1995	15	16	0.94	[0.70; 1.00]	3.2%
Bao 2021	35	35	1.00	[0.90; 1.00]	1.7%
P2011	10	10	1.00	[0.69; 1.00]	1.6%
Tummalapalli 2007	30	30	1.00	[0.88; 1.00]	1.7%
**Random effects model**		**138**	**0.92**	**[0.82; 0.97]**	**27.8%**
Heterogeneity: $I^2 = 36\%$, $\tau^2 = 0.5009$, $p = 0.17$					
**injection_category = 0−1 µl**					
Baia 2007c	4	4	1.00	[0.40; 1.00]	1.5%
Baia 2007d	5	5	1.00	[0.48; 1.00]	1.6%
**Random effects model**		**9**	**0.91**	**[0.56; 0.99]**	**3.1%**
Heterogeneity: $I^2 = 0\%$, $\tau^2 = 0$, $p = 0.92$					
**Random effects model**		**819**	**0.94**	**[0.92; 0.96]**	**100.0%**
Heterogeneity: $I^2 = 0\%$, $\tau^2 = 0$, $p = 0.92$					
Test for subgroup differences: $\chi^2_3 = 3.63$, df = 3 ($p = 0.30$)					

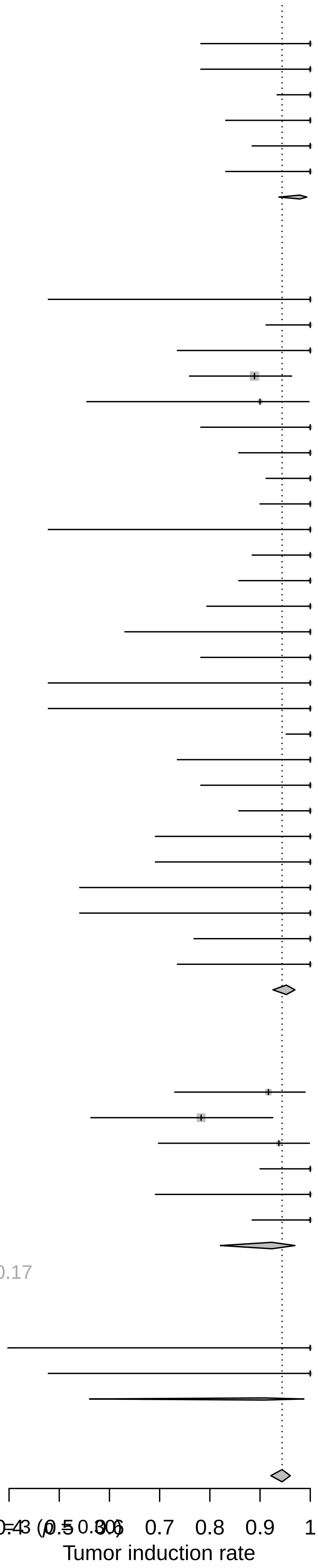

PTM-hetero-injection volume

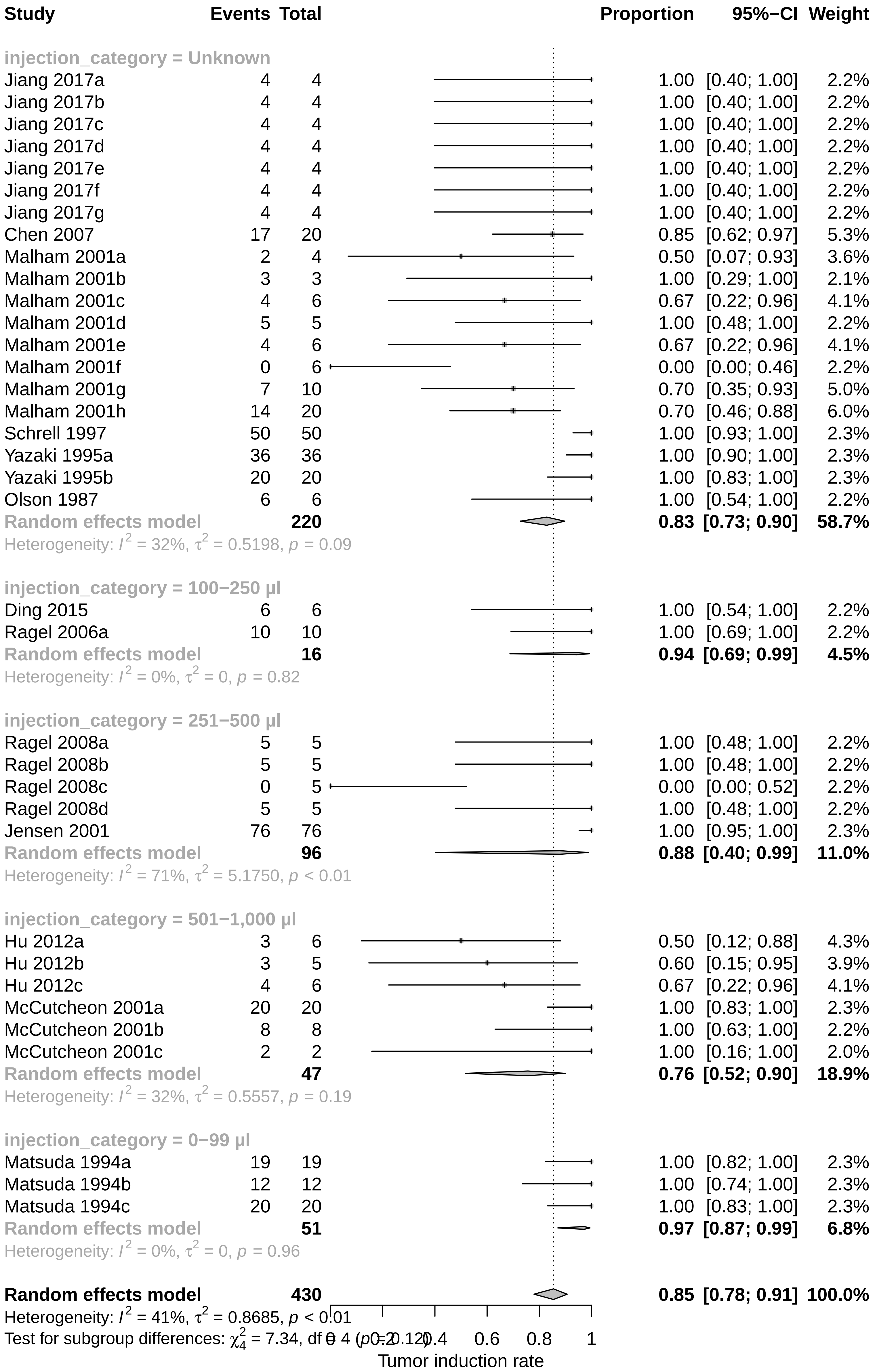

PTM-ortho-injection volume

Study	Events	Total	Proportion	95%−CI	Weight
**injection_category = 2−5 µl**					
Linsler 2021	18	20	0.90	[0.68; 0.99]	4.6%
Zhang 2020a	7	10	0.70	[0.35; 0.93]	4.7%
Zhang 2020b	3	3	1.00	[0.29; 1.00]	3.2%
Zhang 2020c	1	4	0.25	[0.01; 0.81]	3.8%
Zhang 2020d	9	10	0.90	[0.55; 1.00]	4.0%
Zhang 2020e	0	30	0.00	[0.00; 0.12]	3.4%
Zhang 2020f	0	20	0.00	[0.00; 0.17]	3.3%
Zhang 2020g	0	10	0.00	[0.00; 0.31]	3.3%
Zhang 2020h	0	10	0.00	[0.00; 0.31]	3.3%
Zhang 2020i	0	12	0.00	[0.00; 0.26]	3.3%
Zhang 2020j	0	10	0.00	[0.00; 0.31]	3.3%
Zhang 2020k	0	10	0.00	[0.00; 0.31]	3.3%
Zhang 2020l	0	10	0.00	[0.00; 0.31]	3.3%
Zhang 2020m	0	10	0.00	[0.00; 0.31]	3.3%
Zhang 2020n	0	10	0.00	[0.00; 0.31]	3.3%
Zhang 2020o	0	10	0.00	[0.00; 0.31]	3.3%
**Random effects model**		**189**	**0.16**	**[0.05; 0.40]**	**56.9%**
Heterogeneity: $I^2 = 77\%$, $\tau^2 = 4.8806$, $p < 0.01$					
**injection_category = 6−10 µl**					
Friedrich 2012a	34	34	1.00	[0.90; 1.00]	3.4%
Friedrich 2012b	27	29	0.93	[0.77; 0.99]	4.6%
Hu 2012a	0	6	0.00	[0.00; 0.46]	3.3%
Hu 2012b	6	7	0.86	[0.42; 1.00]	4.0%
Hu 2012c	4	6	0.67	[0.22; 0.96]	4.4%
Hu 2012d	6	6	1.00	[0.54; 1.00]	3.3%
Hu 2012e	6	6	1.00	[0.54; 1.00]	3.3%
McCutcheon 2000a	38	45	0.84	[0.71; 0.94]	5.1%
McCutcheon 2000b	24	45	0.53	[0.38; 0.68]	5.2%
McCutcheon 2000c	30	30	1.00	[0.88; 1.00]	3.4%
McCutcheon 2000d	30	30	1.00	[0.88; 1.00]	3.4%
**Random effects model**		**244**	**0.86**	**[0.69; 0.94]**	**43.1%**
Heterogeneity: $I^2 = 75\%$, $\tau^2 = 1.7211$, $p < 0.01$					
**Random effects model**		**433**	**0.47**	**[0.28; 0.68]**	**100.0%**

Heterogeneity: $I^2 = 80\%$, $\tau^2 = 3.5155$, $p < 0.01$

Test for subgroup differences: $\chi^2_1 = 18.18$, df = 1 ($p < 0.01$)

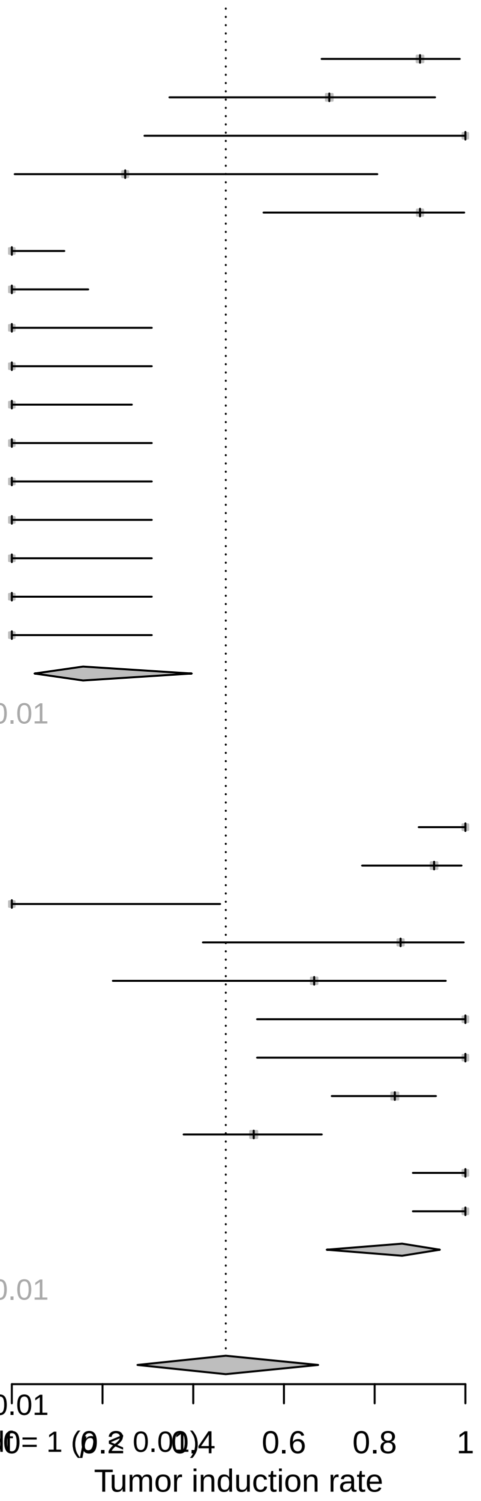

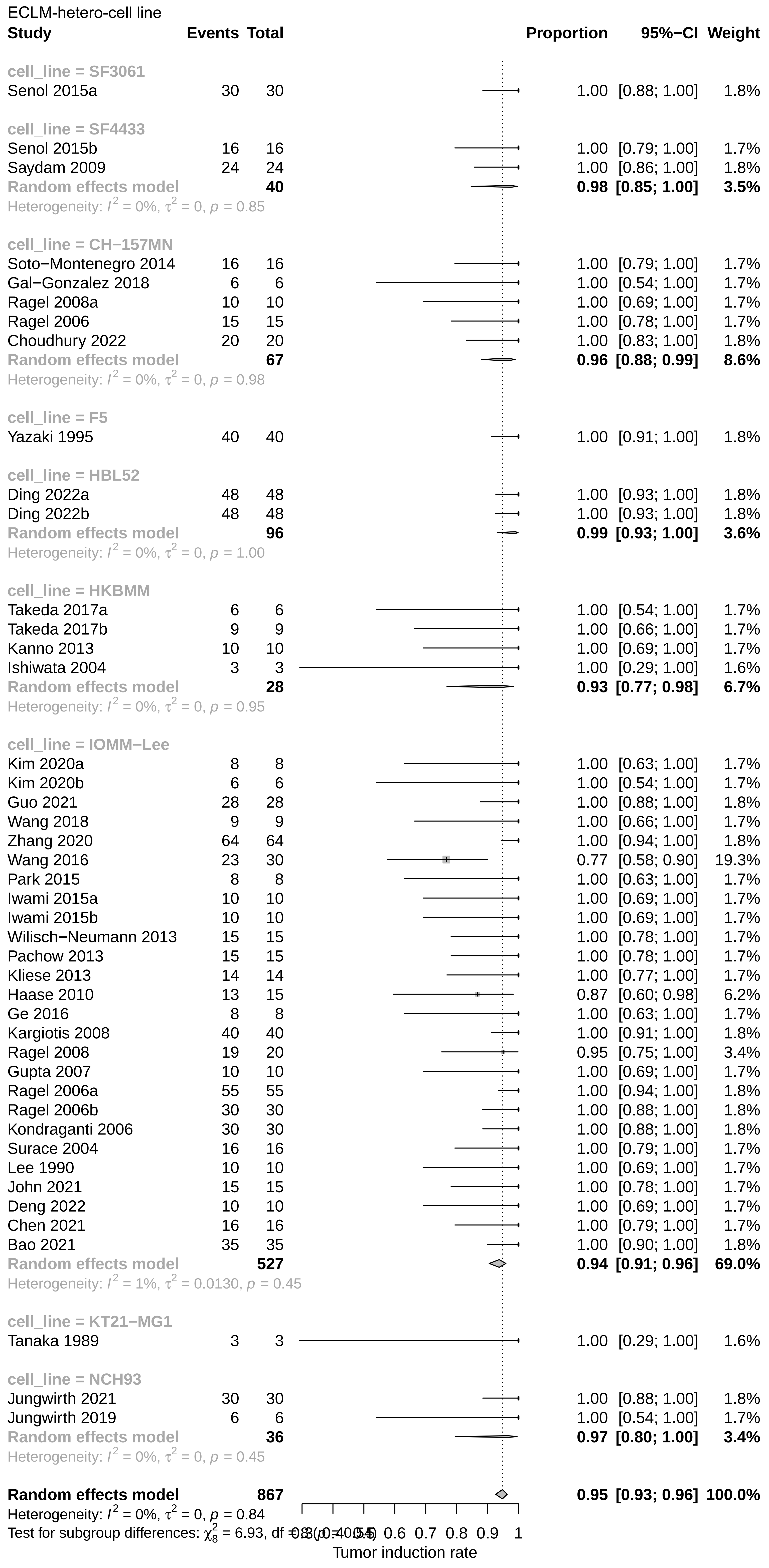
ECLM-hetero-cell line
Study Events Total Proportion 95%−CI Weight
cell_line = SF3061
Senol 2015a 30 30 1.00 [0.88; 1.00] 1.8%
cell_line = SF4433
Senol 2015b 16 16 1.00 [0.79; 1.00] 1.7%
Saydam 2009 24 24 1.00 [0.86; 1.00] 1.8%
Random effects model 40 0.98 [0.85; 1.00] 3.5%
Heterogeneity: I2 = 0%, τ2 = 0, p = 0.85
cell_line = CH−157MN
Soto−Montenegro 2014 16 16 1.00 [0.79; 1.00] 1.7%
Gal−Gonzalez 2018 6 6 1.00 [0.54; 1.00] 1.7%
Ragel 2008a 10 10 1.00 [0.69; 1.00] 1.7%
Ragel 2006 15 15 1.00 [0.78; 1.00] 1.7%
Choudhury 2022 20 20 1.00 [0.83; 1.00] 1.8%
Random effects model 67 0.96 [0.88; 0.99] 8.6%
Heterogeneity: I2 = 0%, τ2 = 0, p = 0.98
cell_line = F5
Yazaki 1995 40 40 1.00 [0.91; 1.00] 1.8%
cell_line = HBL52
Ding 2022a 48 48 1.00 [0.93; 1.00] 1.8%
Ding 2022b 48 48 1.00 [0.93; 1.00] 1.8%
Random effects model 96 0.99 [0.93; 1.00] 3.6%
Heterogeneity: I2 = 0%, τ2 = 0, p = 1.00
cell_line = HKBMM
Takeda 2017a 6 6 1.00 [0.54; 1.00] 1.7%
Takeda 2017b 9 9 1.00 [0.66; 1.00] 1.7%
Kanno 2013 10 10 1.00 [0.69; 1.00] 1.7%
Ishiwata 2004 3 3 1.00 [0.29; 1.00] 1.6%
Random effects model 28 0.93 [0.77; 0.98] 6.7%
Heterogeneity: I2 = 0%, τ2 = 0, p = 0.95
cell_line = IOMM−Lee
Kim 2020a 8 8 1.00 [0.63; 1.00] 1.7%
Kim 2020b 6 6 1.00 [0.54; 1.00] 1.7%
Guo 2021 28 28 1.00 [0.88; 1.00] 1.8%
Wang 2018 9 9 1.00 [0.66; 1.00] 1.7%
Zhang 2020 64 64 1.00 [0.94; 1.00] 1.8%
Wang 2016 23 30 0.77 [0.58; 0.90] 19.3%
Park 2015 8 8 1.00 [0.63; 1.00] 1.7%
Iwami 2015a 10 10 1.00 [0.69; 1.00] 1.7%
Iwami 2015b 10 10 1.00 [0.69; 1.00] 1.7%
Wilisch−Neumann 2013 15 15 1.00 [0.78; 1.00] 1.7%
Pachow 2013 15 15 1.00 [0.78; 1.00] 1.7%
Kliese 2013 14 14 1.00 [0.77; 1.00] 1.7%
Haase 2010 13 15 0.87 [0.60; 0.98] 6.2%
Ge 2016 8 8 1.00 [0.63; 1.00] 1.7%
Kargiotis 2008 40 40 1.00 [0.91; 1.00] 1.8%
Ragel 2008 19 20 0.95 [0.75; 1.00] 3.4%
Gupta 2007 10 10 1.00 [0.69; 1.00] 1.7%
Ragel 2006a 55 55 1.00 [0.94; 1.00] 1.8%
Ragel 2006b 30 30 1.00 [0.88; 1.00] 1.8%
Kondraganti 2006 30 30 1.00 [0.88; 1.00] 1.8%
Surace 2004 16 16 1.00 [0.79; 1.00] 1.7%
Lee 1990 10 10 1.00 [0.69; 1.00] 1.7%
John 2021 15 15 1.00 [0.78; 1.00] 1.7%
Deng 2022 10 10 1.00 [0.69; 1.00] 1.7%
Chen 2021 16 16 1.00 [0.79; 1.00] 1.7%
Bao 2021 35 35 1.00 [0.90; 1.00] 1.8%
Random effects model 527 0.94 [0.91; 0.96] 69.0%
Heterogeneity: I2 = 1%, τ2 = 0.0130, p = 0.45
cell_line = KT21−MG1
Tanaka 1989 3 3 1.00 [0.29; 1.00] 1.6%
cell_line = NCH93
Jungwirth 2021 30 30 1.00 [0.88; 1.00] 1.8%
Jungwirth 2019 6 6 1.00 [0.54; 1.00] 1.7%
Random effects model 36 0.97 [0.80; 1.00] 3.4%
Heterogeneity: I2 = 0%, τ2 = 0, p = 0.45
Random effects model 867 0.95 [0.93; 0.96] 100.0%
Heterogeneity: I2 = 0%, τ2 = 0, p = 0.84
Test for subgroup differences: χ2 8 = 6.93, df = 8 (p = 0.54)
0.3 0.4 0.5 0.6 0.7 0.8 0.9 1
Tumor induction rate

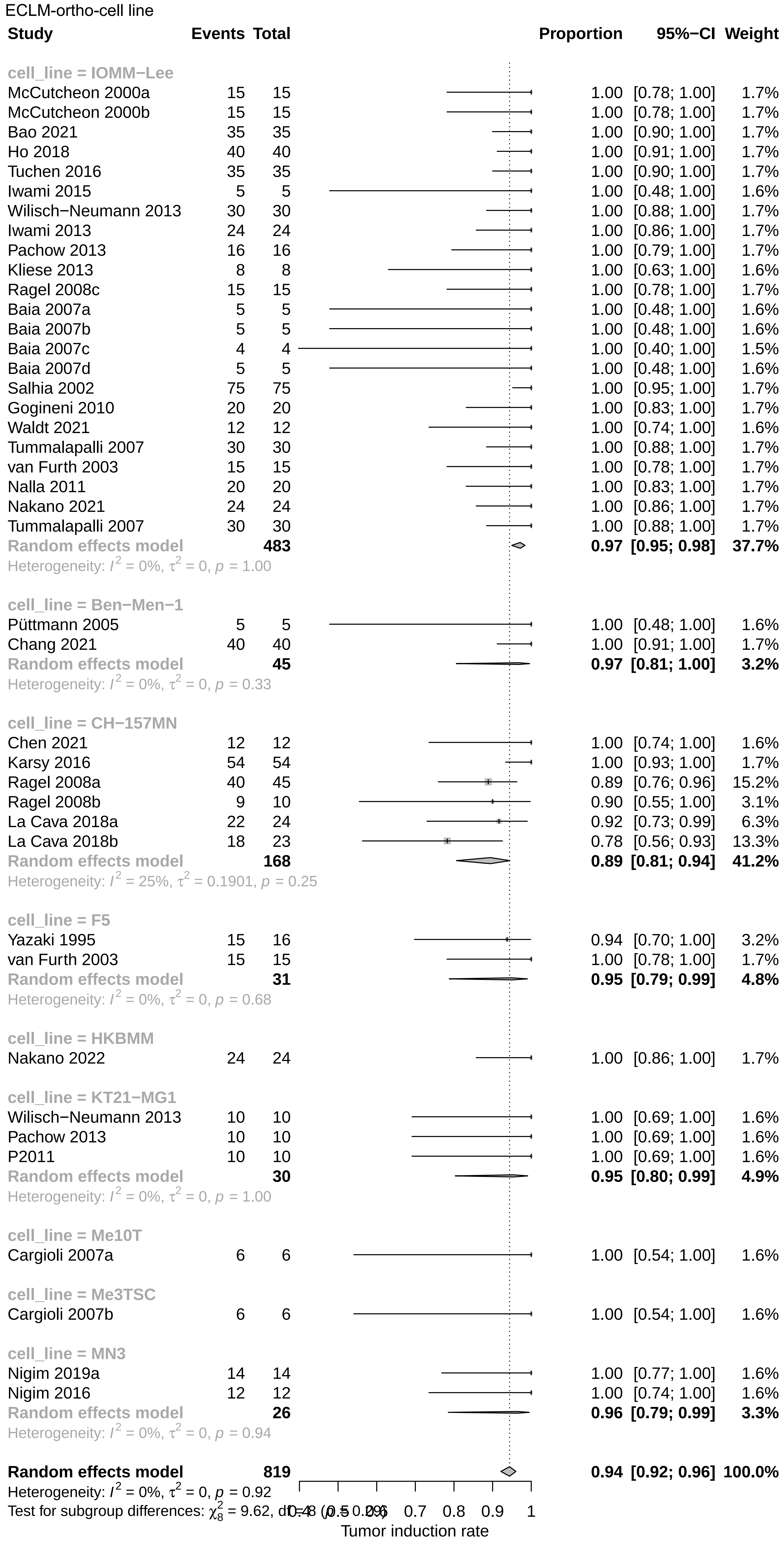
ECLM-ortho-cell line
Study Events Total Proportion 95%−CI Weight
cell_line = IOMM−Lee
McCutcheon 2000a 15 15 1.00 [0.78; 1.00] 1.7%
McCutcheon 2000b 15 15 1.00 [0.78; 1.00] 1.7%
Bao 2021 35 35 1.00 [0.90; 1.00] 1.7%
Ho 2018 40 40 1.00 [0.91; 1.00] 1.7%
Tuchen 2016 35 35 1.00 [0.90; 1.00] 1.7%
Iwami 2015 5 5 1.00 [0.48; 1.00] 1.6%
Wilisch−Neumann 2013 30 30 1.00 [0.88; 1.00] 1.7%
Iwami 2013 24 24 1.00 [0.86; 1.00] 1.7%
Pachow 2013 16 16 1.00 [0.79; 1.00] 1.7%
Kliese 2013 8 8 1.00 [0.63; 1.00] 1.6%
Ragel 2008c 15 15 1.00 [0.78; 1.00] 1.7%
Baia 2007a 5 5 1.00 [0.48; 1.00] 1.6%
Baia 2007b 5 5 1.00 [0.48; 1.00] 1.6%
Baia 2007c 4 4 1.00 [0.40; 1.00] 1.5%
Baia 2007d 5 5 1.00 [0.48; 1.00] 1.6%
Salhia 2002 75 75 1.00 [0.95; 1.00] 1.7%
Gogineni 2010 20 20 1.00 [0.83; 1.00] 1.7%
Waldt 2021 12 12 1.00 [0.74; 1.00] 1.6%
Tummalapalli 2007 30 30 1.00 [0.88; 1.00] 1.7%
van Furth 2003 15 15 1.00 [0.78; 1.00] 1.7%
Nalla 2011 20 20 1.00 [0.83; 1.00] 1.7%
Nakano 2021 24 24 1.00 [0.86; 1.00] 1.7%
Tummalapalli 2007 30 30 1.00 [0.88; 1.00] 1.7%
Random effects model 483 0.97 [0.95; 0.98] 37.7%
Heterogeneity: I^2 = 0%, τ^2 = 0, p = 1.00
cell_line = Ben−Men−1
Püttmann 2005 5 5 1.00 [0.48; 1.00] 1.6%
Chang 2021 40 40 1.00 [0.91; 1.00] 1.7%
Random effects model 45 0.97 [0.81; 1.00] 3.2%
Heterogeneity: I^2 = 0%, τ^2 = 0, p = 0.33
cell_line = CH−157MN
Chen 2021 12 12 1.00 [0.74; 1.00] 1.6%
Karsy 2016 54 54 1.00 [0.93; 1.00] 1.7%
Ragel 2008a 40 45 0.89 [0.76; 0.96] 15.2%
Ragel 2008b 9 10 0.90 [0.55; 1.00] 3.1%
La Cava 2018a 22 24 0.92 [0.73; 0.99] 6.3%
La Cava 2018b 18 23 0.78 [0.56; 0.93] 13.3%
Random effects model 168 0.89 [0.81; 0.94] 41.2%
Heterogeneity: I^2 = 25%, τ^2 = 0.1901, p = 0.25
cell_line = F5
Yazaki 1995 15 16 0.94 [0.70; 1.00] 3.2%
van Furth 2003 15 15 1.00 [0.78; 1.00] 1.7%
Random effects model 31 0.95 [0.79; 0.99] 4.8%
Heterogeneity: I^2 = 0%, τ^2 = 0, p = 0.68
cell_line = HKBMM
Nakano 2022 24 24 1.00 [0.86; 1.00] 1.7%
cell_line = KT21−MG1
Wilisch−Neumann 2013 10 10 1.00 [0.69; 1.00] 1.6%
Pachow 2013 10 10 1.00 [0.69; 1.00] 1.6%
P2011 10 10 1.00 [0.69; 1.00] 1.6%
Random effects model 30 0.95 [0.80; 0.99] 4.9%
Heterogeneity: I^2 = 0%, τ^2 = 0, p = 1.00
cell_line = Me10T
Cargioli 2007a 6 6 1.00 [0.54; 1.00] 1.6%
cell_line = Me3TSC
Cargioli 2007b 6 6 1.00 [0.54; 1.00] 1.6%
cell_line = MN3
Nigim 2019a 14 14 1.00 [0.77; 1.00] 1.6%
Nigim 2016 12 12 1.00 [0.74; 1.00] 1.6%
Random effects model 26 0.96 [0.79; 0.99] 3.3%
Heterogeneity: I^2 = 0%, τ^2 = 0, p = 0.94
Random effects model 819 0.94 [0.92; 0.96] 100.0%
Heterogeneity: I^2 = 0%, τ^2 = 0, p = 0.92
Test for subgroup differences: χ^2_8 = 9.62, df = 8 (p = 0.29)
0.4 0.5 0.6 0.7 0.8 0.9 1
Tumor induction rate

PTM-hetero-WHO-grade

Study	Events	Total	Proportion	95%−CI	Weight
**who_grade = Unknown**					
Jiang 2017a	4	4	1.00	[0.40; 1.00]	2.2%
Jiang 2017b	4	4	1.00	[0.40; 1.00]	2.2%
Jiang 2017c	4	4	1.00	[0.40; 1.00]	2.2%
Jiang 2017d	4	4	1.00	[0.40; 1.00]	2.2%
Jiang 2017e	4	4	1.00	[0.40; 1.00]	2.2%
Jiang 2017f	4	4	1.00	[0.40; 1.00]	2.2%
Jiang 2017g	4	4	1.00	[0.40; 1.00]	2.2%
Schrell 1997	50	50	1.00	[0.93; 1.00]	2.3%
**Random effects model**		**78**	**0.93**	**[0.82; 0.97]**	**17.4%**
Heterogeneity: $I^2 = 0\%$, $\tau^2 = 0$, $p = 0.93$					
**who_grade = 3**					
Ding 2015	6	6	1.00	[0.54; 1.00]	2.2%
Hu 2012a	3	6	0.50	[0.12; 0.88]	4.3%
Hu 2012b	3	5	0.60	[0.15; 0.95]	3.9%
Hu 2012c	4	6	0.67	[0.22; 0.96]	4.1%
McCutcheon 2001c	2	2	1.00	[0.16; 1.00]	2.0%
Yazaki 1995b	20	20	1.00	[0.83; 1.00]	2.3%
**Random effects model**		**45**	**0.75**	**[0.51; 0.89]**	**18.9%**
Heterogeneity: $I^2 = 28\%$, $\tau^2 = 0.4485$, $p = 0.23$					
**who_grade = 1**					
Ragel 2008a	5	5	1.00	[0.48; 1.00]	2.2%
Ragel 2008b	5	5	1.00	[0.48; 1.00]	2.2%
Ragel 2008c	0	5	0.00	[0.00; 0.52]	2.2%
Ragel 2008d	5	5	1.00	[0.48; 1.00]	2.2%
Chen 2007	17	20	0.85	[0.62; 0.97]	5.3%
Ragel 2006a	10	10	1.00	[0.69; 1.00]	2.2%
Malham 2001a	2	4	0.50	[0.07; 0.93]	3.6%
Malham 2001b	3	3	1.00	[0.29; 1.00]	2.1%
Malham 2001c	4	6	0.67	[0.22; 0.96]	4.1%
Malham 2001d	5	5	1.00	[0.48; 1.00]	2.2%
Malham 2001g	7	10	0.70	[0.35; 0.93]	5.0%
Malham 2001h	14	20	0.70	[0.46; 0.88]	6.0%
Jensen 2001	76	76	1.00	[0.95; 1.00]	2.3%
McCutcheon 2001a	20	20	1.00	[0.83; 1.00]	2.3%
Yazaki 1995a	36	36	1.00	[0.90; 1.00]	2.3%
Matsuda 1994a	19	19	1.00	[0.82; 1.00]	2.3%
Matsuda 1994b	12	12	1.00	[0.74; 1.00]	2.3%
Matsuda 1994c	20	20	1.00	[0.83; 1.00]	2.3%
Olson 1987	6	6	1.00	[0.54; 1.00]	2.2%
**Random effects model**		**287**	**0.88**	**[0.78; 0.94]**	**55.2%**
Heterogeneity: $I^2 = 48\%$, $\tau^2 = 1.0307$, $p = 0.01$					
**who_grade = 2**					
Malham 2001e	4	6	0.67	[0.22; 0.96]	4.1%
Malham 2001f	0	6	0.00	[0.00; 0.46]	2.2%
McCutcheon 2001b	8	8	1.00	[0.63; 1.00]	2.2%
**Random effects model**		**20**	**0.59**	**[0.09; 0.95]**	**8.6%**
Heterogeneity: $I^2 = 71\%$, $\tau^2 = 3.8504$, $p = 0.03$					
**Random effects model**		**430**	**0.85**	**[0.78; 0.91]**	**100.0%**

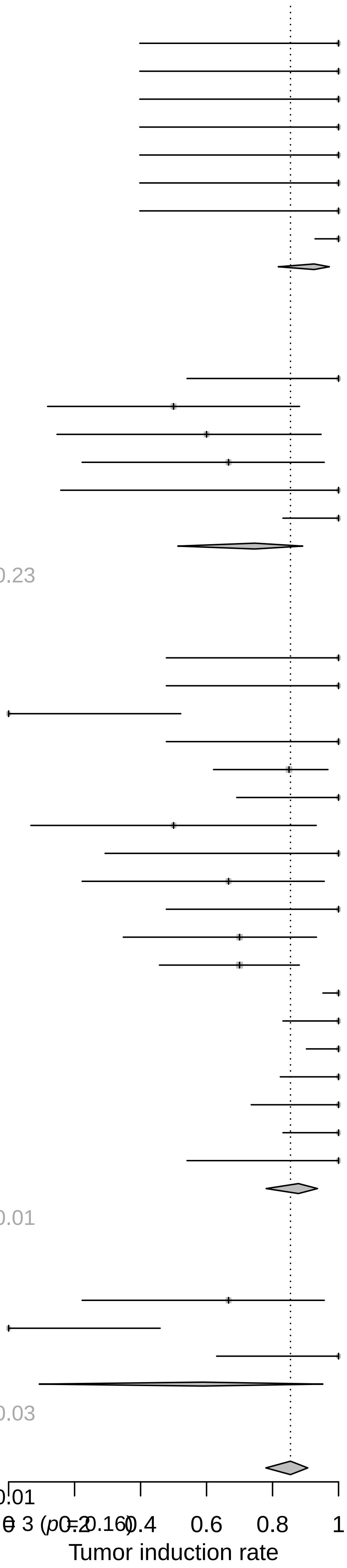

Heterogeneity: $I^2 = 41\%$, $\tau^2 = 0.8685$, $p < 0.01$

Test for subgroup differences: $\chi^2_3 = 5.18$, df = 3 ($p = 0.16$)

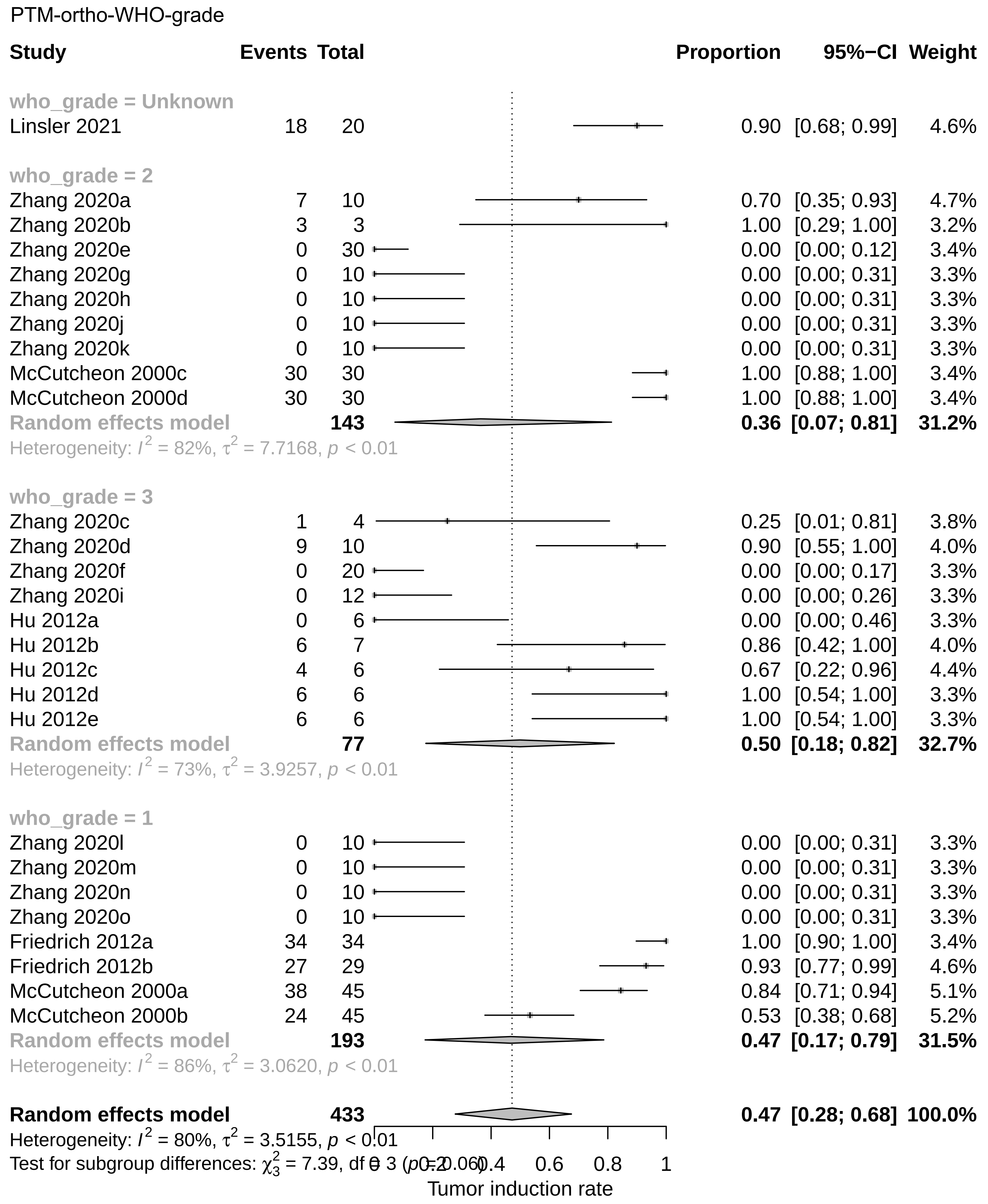
PTM-ortho-WHO-grade
Study Events Total Proportion 95%−CI Weight
who_grade = Unknown
Linsler 2021 18 20 0.90 [0.68; 0.99] 4.6%
who_grade = 2
Zhang 2020a 7 10 0.70 [0.35; 0.93] 4.7%
Zhang 2020b 3 3 1.00 [0.29; 1.00] 3.2%
Zhang 2020e 0 30 0.00 [0.00; 0.12] 3.4%
Zhang 2020g 0 10 0.00 [0.00; 0.31] 3.3%
Zhang 2020h 0 10 0.00 [0.00; 0.31] 3.3%
Zhang 2020j 0 10 0.00 [0.00; 0.31] 3.3%
Zhang 2020k 0 10 0.00 [0.00; 0.31] 3.3%
McCutcheon 2000c 30 30 1.00 [0.88; 1.00] 3.4%
McCutcheon 2000d 30 30 1.00 [0.88; 1.00] 3.4%
Random effects model 143 0.36 [0.07; 0.81] 31.2%
Heterogeneity: I^2 = 82%, τ^2 = 7.7168, p < 0.01
who_grade = 3
Zhang 2020c 1 4 0.25 [0.01; 0.81] 3.8%
Zhang 2020d 9 10 0.90 [0.55; 1.00] 4.0%
Zhang 2020f 0 20 0.00 [0.00; 0.17] 3.3%
Zhang 2020i 0 12 0.00 [0.00; 0.26] 3.3%
Hu 2012a 0 6 0.00 [0.00; 0.46] 3.3%
Hu 2012b 6 7 0.86 [0.42; 1.00] 4.0%
Hu 2012c 4 6 0.67 [0.22; 0.96] 4.4%
Hu 2012d 6 6 1.00 [0.54; 1.00] 3.3%
Hu 2012e 6 6 1.00 [0.54; 1.00] 3.3%
Random effects model 77 0.50 [0.18; 0.82] 32.7%
Heterogeneity: I^2 = 73%, τ^2 = 3.9257, p < 0.01
who_grade = 1
Zhang 2020l 0 10 0.00 [0.00; 0.31] 3.3%
Zhang 2020m 0 10 0.00 [0.00; 0.31] 3.3%
Zhang 2020n 0 10 0.00 [0.00; 0.31] 3.3%
Zhang 2020o 0 10 0.00 [0.00; 0.31] 3.3%
Friedrich 2012a 34 34 1.00 [0.90; 1.00] 3.4%
Friedrich 2012b 27 29 0.93 [0.77; 0.99] 4.6%
McCutcheon 2000a 38 45 0.84 [0.71; 0.94] 5.1%
McCutcheon 2000b 24 45 0.53 [0.38; 0.68] 5.2%
Random effects model 193 0.47 [0.17; 0.79] 31.5%
Heterogeneity: I^2 = 86%, τ^2 = 3.0620, p < 0.01
Random effects model 433 0.47 [0.28; 0.68] 100.0%
Heterogeneity: I^2 = 80%, τ^2 = 3.5155, p < 0.01
Test for subgroup differences: χ^2_3 = 7.39, df = 3 (p = 0.06)
0 0.2 0.4 0.6 0.8 1
Tumor induction rate
